# Interfacial Chemistry in the Electrocatalytic Hydrogenation of CO_2_ over C-Supported Cu-Based Systems

**DOI:** 10.1021/acscatal.3c01288

**Published:** 2023-04-14

**Authors:** Diego Gianolio, Michael D. Higham, Matthew G. Quesne, Matteo Aramini, Ruoyu Xu, Alex I. Large, Georg Held, Juan-Jesús Velasco-Vélez, Michael Haevecker, Axel Knop-Gericke, Chiara Genovese, Claudio Ampelli, Manfred Erwin Schuster, Siglinda Perathoner, Gabriele Centi, C. Richard A. Catlow, Rosa Arrigo

**Affiliations:** † Diamond Light Source Ltd., Harwell Science & Innovation Campus, Didcot, Oxfordshire OX11 0DE, U.K.; ‡ Cardiff Catalysis Institute, School of Chemistry, Cardiff University, Main Building, Park Place, Cardiff, Wales CF10 3AT, U.K.; § UK Catalysis Hub, Research Complex at Harwell, Rutherford Appleton Laboratory, R92, Harwell, Oxfordshire OX11 0FA, U.K.; ∥ Department of Chemistry, University College London, 20 Gordon Street, London WC1H 0AJ, U.K.; ⊥ Department of Chemical Engineering, University College London, 20 Gordon Street, London WC1H 0AJ, U.K.; # Max-Planck-Institut für Chemische Energiekonversion, Stiftstrasse 34-36, 45470 Mülheim an der Ruhr, Germany; ∇ Department of Inorganic Chemistry, Fritz-Haber-Institut der Max-Planck Gesellschaft, Faradayweg 4-6, 14195 Berlin, Germany; ○ Department of ChiBioFarAm, ERIC aisbl and CASPE/INSTM, University of Messina, V. le F.Stagno D’ Alcontres 31, 98166 Messina, Italy; ◆ Johnson Matthey Technology Centre, Reading RG4 9NH, U.K.; ¶ School of Science, Engineering and Environment, University of Salford, Cockcroft Building, Salford, Greater Manchester M5 4WT, U.K.

**Keywords:** operando spectroscopy, CO_2_RR, Cu, Zn, Fe electrocatalysts, DFT, XAFS

## Abstract

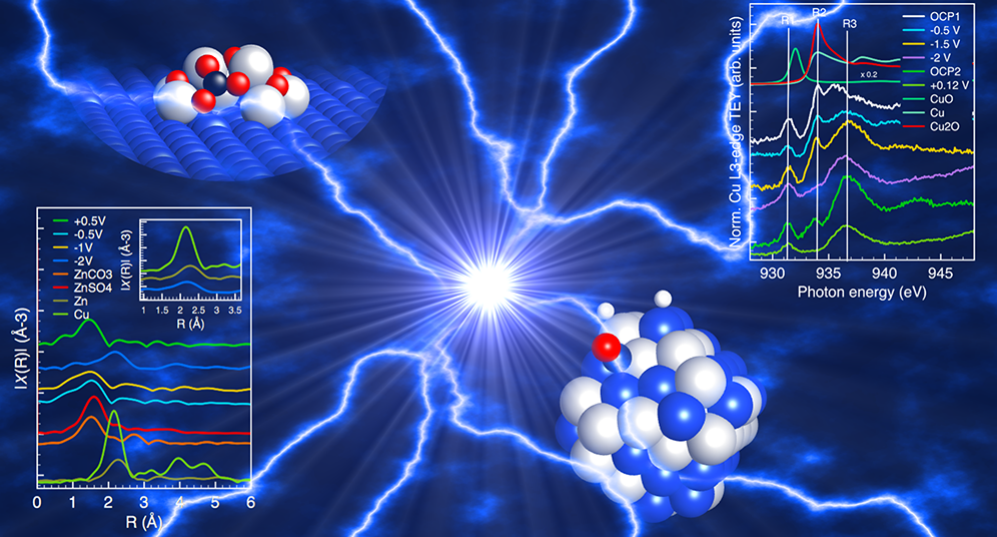

Operando soft and hard X-ray spectroscopic techniques were used in combination with plane-wave density functional theory (DFT) simulations to rationalize the enhanced activities of Zn-containing Cu nanostructured electrocatalysts in the electrocatalytic CO_2_ hydrogenation reaction. We show that at a potential for CO_2_ hydrogenation, Zn is alloyed with Cu in the bulk of the nanoparticles with no metallic Zn segregated; at the interface, low reducible Cu(I)–O species are consumed. Additional spectroscopic features are observed, which are identified as various surface Cu(I) ligated species; these respond to the potential, revealing characteristic interfacial dynamics. Similar behavior was observed for the Fe–Cu system in its active state, confirming the general validity of this mechanism; however, the performance of this system deteriorates after successive applied cathodic potentials, as the hydrogen evolution reaction then becomes the main reaction pathway. In contrast to an active system, Cu(I)–O is now consumed at cathodic potentials and not reversibly reformed when the voltage is allowed to equilibrate at the open-circuit voltage; rather, only the oxidation to Cu(II) is observed. We show that the Cu–Zn system represents the optimal active ensembles with stabilized Cu(I)–O; DFT simulations rationalize this observation by indicating that Cu–Zn–O neighboring atoms are able to activate CO_2_, whereas Cu–Cu sites provide the supply of H atoms for the hydrogenation reaction. Our results demonstrate an electronic effect exerted by the heterometal, which depends on its intimate distribution within the Cu phase and confirms the general validity of these mechanistic insights for future electrocatalyst design strategies.

## 1 Introduction

The direct electrocatalytic hydrogenation of CO_2_ using renewable energy offers several advantages with respect to the multistep thermocatalytic routes from CO_2_ and H_2_ (power-to-gas technologies).^1−4^ These advantages include improved efficiencies, reduction of capital/operative costs, improved process intensification, and more compact design for distributed uses.^4−7^ Cu is a component of many electrocatalysts for the CO_2_ reduction reaction (CO_2_RR) due to its higher efficiency and selectivity toward CO_2_ reduction products over the parasitic hydrogen evolution reaction (HER).^8−14^ Yet, the electrocatalyst performances reported so far, in terms of activity and stability, are not sufficient to warrant further development. As such, superior electrocatalysts must be designed if they are to be developed into a commercial technology.

Many factors have been identified in determining electrocatalysts’ performance, including surface nanostructure and composition,^8,15−17^ mesoscale structural and textural characteristics,^18^ reaction conditions (pH, buffer strength, ion effects),^19^ and mass transport-related effects.^20−22^ Various attempts at nanostructural and morphological control of the Cu electrocatalysts have unveiled important insights into the structure sensitivity of this reaction;^16,17,23^ however, these design strategies have proved ineffective for attaining the required electrode selectivity and stability. An in-depth understanding of the mechanistic aspects of the electrocatalytic reaction is key for improved material design.^24,25^ Bulk-sensitive, operando spectroscopy studies for CO_2_ electroconversion in the hard X-ray regime (energy range, 5–35 keV) are widely reported^26^ with the aim of aiding electrocatalyst design by providing insights into the underlying reaction and electrode degradation mechanisms. Interestingly, the bulk electronic structure of various nanostructured Cu electrodes obtained via different synthetic methods and with different morphologies consistently showed a metallic bulk under operando conditions for liquid phase CO_2_ reduction.^27−29^ Consequently, these bulk-sensitive spectroscopy studies appear to be insufficient to explain in full the reactivity of Cu in CO_2_RR, and the underlying reaction mechanism driving the selectivity toward CO_2_ reduction products remains under debate.

In terms of morphological changes, the results from the literature are less consistent, and various observations are discussed in the context of the reaction mechanism: (i) Cu electrodes of different nanostructures have shown transformation into rounder morphologies or dendrites, strongly depending on the applied potential rather than the initial characteristic of the Cu electrode;^30^ (ii) a transformation of the nanoparticles (NPs) edges from rounder into more ordered steps under CO_2_ reduction potential was attributed to the formation of CO chemisorption-induced (100) steps active in C–C coupling;^31^ (iii) the high stability of several planes of Cu nanoparticles under cathodic potentials undergoes morphological changes solely due to the presence of O_2_ impurities.^32^

On account of this third factor, a few dissolved oxygen species on Cu electrodes were identified by some authors^33,34^ and suggested as a prerequisite species for CO_2_ activation; their loss under reducing potentials led to deactivation.

Capturing dynamics at the thin electroactive interface formed over Cu is challenging by bulk-sensitive techniques, especially under cathodic conditions in which the oxygen chemical potential is very low. A surface/interface-sensitive characterization of the reactive interface allows a more robust structure–function relationship and brings our understanding of the CO_2_RR mechanism beyond the state-of-the-art. Specifically, electron detection-based soft X-ray spectroscopic methods (energy range, 50–1500 eV) are not only sensitive toward the liquid/solid interface but also allow the monitoring of the dynamics of light elements such as O and C during the electrocatalytic reaction, providing an unprecedented description of the reactive interface. However, electron detection carries experimental challenges for the characterization of the liquid/solid interface due to the requirement of operating in ultra-high vacuum (UHV) conditions, which constrain the measurement of thin films of liquid on an electrode surface.^35^ Consequently, in situ surface-sensitive X-ray spectroscopy studies of electrified interfaces remain niche applications in electrocatalysis.^26^ To overcome this challenge, in this study, we use a cell configuration approach that enables the trapping of a thin film of electrolyte within the pores of the electrode by using a graphene overlayer as a sealing and electron transparent membrane.^35^

For the mechanistic study, we have turned our attention to Cu-bimetallic systems because, from the literature,^36−39^ the additional metal, generally less noble than Cu (e. g. Zn, Si, etc.), is shown to induce specific chemisorption sites for key intermediates, opening up specific reaction channels.^40^ Particularly, the Cu–Zn system has been a subject of much interest due to a general observation of a beneficial effect in CO_2_RR;^41−43^ however, a detailed mechanism that is required to underpin the precise role of Zn remains undisclosed. By modulating the activity/selectivity of Cu in bimetallic systems, we expect to reveal specific dynamics related to the observed electrocatalytic performances. We have selected here gas diffusion layer-based Cu and bimetallic Cu–Zn and Cu–Fe electrodes prepared via wet chemistry, showing a different reactivity and selectivity consistent with literature data,^40^ with the Cu–Zn system being more CO_2_RR active and selective toward hydrogenated products. Our aim is to understand the role of Zn in suppressing the HER while at the same time directing the selectivity toward CO_2_ hydrogenation beyond CO formation.

By means of Cu and Zn K-edge hard X-ray absorption fine structure (XAFS) spectroscopy and Cu L-, O K-, and C K-edges soft near-edge X-ray absorption fine structure (NEXAFS) spectroscopy, we are able to reveal the origin of the activity-enhancing role of Zn, which is intimately dispersed into the Cu(II/I) phase at the surface and is found to exert a doping effect initially, later becoming alloyed with Cu under CO_2_RR potentials in bulk, while stabilizing Cu(I)–O species at the interphase against reduction. Density functional theory (DFT) calculations on specifically modeled surfaces were carried out to rationalize these findings in terms of the adsorption energy of reactants, products, and intermediate species. The present work provides valuable insights into the reactivity of this class of electrocatalysts in CO_2_RR and their dynamical structural transformation and corroborates the importance of combined in situ methods to extract relevant information for the tailored design of electrocatalysts. We show the presence of an electrified interface populated by various adsorbed species both relevant for the CO_2_RR as well as the HER. By a comparative analysis with Cu–Fe systems, we can show that the mechanistic aspects presented for the Cu–Zn systems are of general relevance and can be used as a model for achieving high activity and selectivity toward methanation reaction or even methanol synthesis.

## 2 Results and Discussion

### 2.1 Electronic Structure and Morphology of the As-Prepared Electrocatalysts

**Figure 1:**
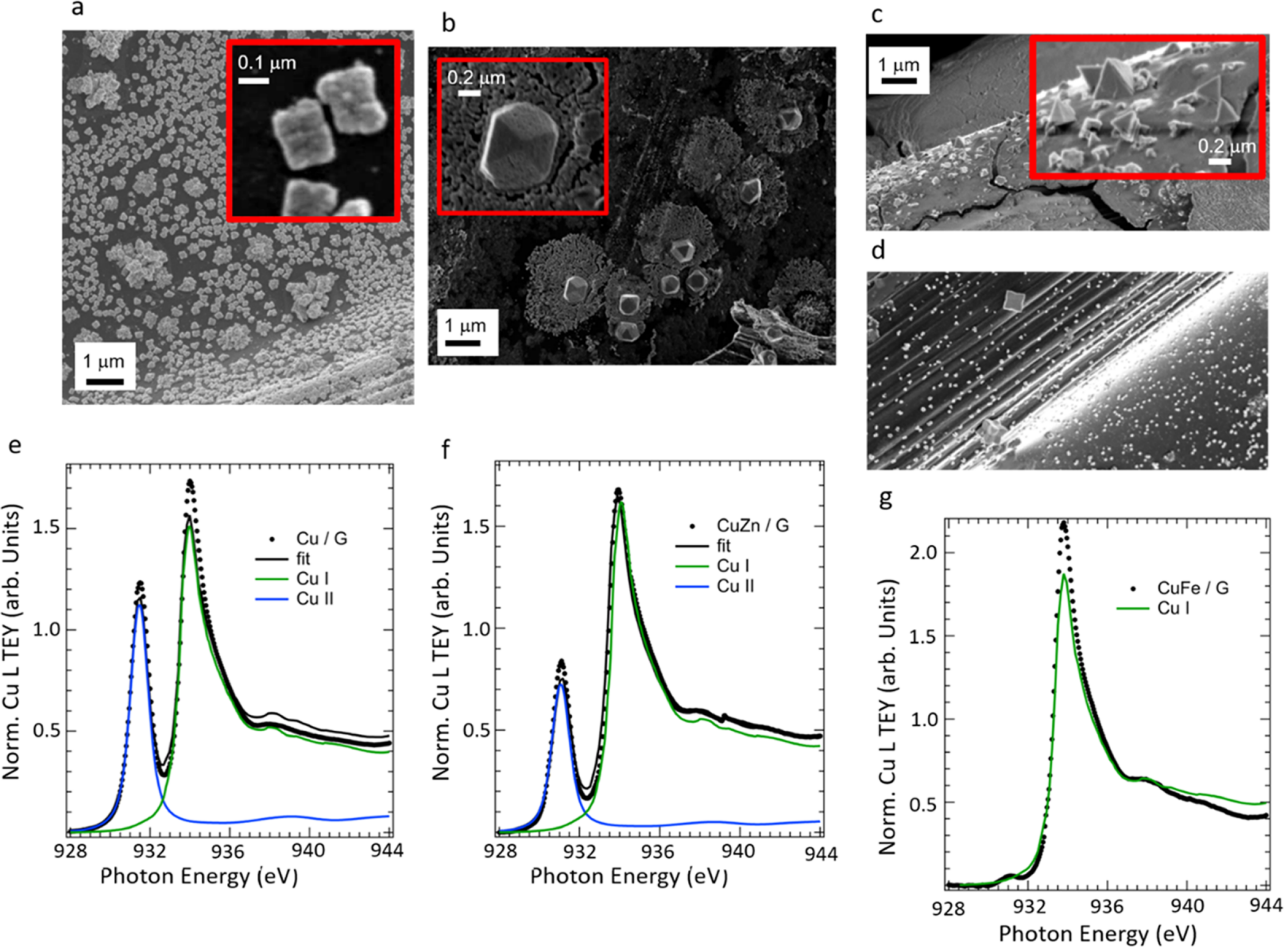
SEM micrographs of as-prepared samples and region of interest of the same micrograph with higher magnification. Inset: Cu/G (a), Cu–Zn/G (b), and Cu–Fe/G (c, d). Cu L-edge NEXAFS of the as-prepared samples: Cu/G (e), Cu–Zn/G (f), and Cu–Fe/G (g).

Raman spectra in Figure S1, taken over regions without and with electrodeposited Cu, show the highly graphitic character of the bulk of the gas diffusion layer carbon support, which remains unchanged after electrodeposition. The morphology and surface electronic structure of Cu/G, CuZn/G, and CuFe/G are characterized by means of scanning electron microscopy (SEM) and Cu L_2,3_-edge near-edge absorption spectroscopy (NEXAFS) and reported in Figure 1a–d,e–g, respectively. The elemental mappings determined by energy-dispersive X-ray (EDX) spectroscopy during SEM investigations are reported in Figure S2 of the Supporting Information.

The electrodeposited Cu/G is characterized by cube-shaped nanoparticles with rounded edges (Figure 1a), agglomerated or loosely sparse on the carbon fibers’ surface. The inset in Figure 1a shows the characteristic cauliflower-like morphology of the cube-shaped nanoparticle agglomerates. The Cu elemental mapping (Figure S2a) confirms a rather homogeneous distribution of Cu on the support, although the density of the particles varies. Additionally, the Cu EDX mapping (Figure S2a) shows Cu signals in some areas in which particles are not clearly visible in the corresponding SEM image measured in secondary electron (SE) mode at 15 kV, suggesting that these nanostructures are rather thin.

The morphology of the Cu nanostructures in the CuZn/G system (Figure 1b) is very different from the starting electrodeposited Cu. Here, a fraction of the Cu nanoparticles (NPs) has transformed into larger cuboctahedral Cu particles. These cuboctahedrons expose both (111) and (100) planes.^44,45^

The higher magnification SEM images (Figure 1b, inset) for CuZn/G show that the cuboctahedrons have pores and are embedded within islands of a highly porous film. The EDX elemental mapping (Figure S2b) shows that both Cu and Zn are homogeneously distributed within the particles and in the surrounding structures. In contrast, the CuFe/G system is highly heterogeneous in terms of the particles’ morphology. Figure 1c shows a region of the carbon fibers with a thick overlayer partially detached from the fibers, which is composed of both Fe and Cu as determined by EDX elemental mapping (Figure S2c). On top of the thick layer, prismatic particles are observed (inset in Figure 1c), exposing (111) facets. Figure 1d exemplifies other areas found on this sample, in which small cuboid particles of different sizes are deposited on the C support. In cubic particles, the (100) facets are exposed.^44,45^ The elemental mapping of this region (Figure S2d) shows that the larger particles contain both Cu and Fe, but Cu is predominant; the carbon support also presents smaller nanostructures homogeneously distributed, where Fe is also homogeneously dispersed, more abundantly than Cu.

The NEXAFS spectra measured for all of the samples in UHV and in total electron yield (TEY) mode at the Cu L_2,3_-edges are reported in Figure 1e–g. Cu(I) is the dominant oxidation state in all of the samples, as indicated by the intense Cu L_3_-edge resonance at approximately 934 eV, which was assigned to the 2p → 4p transition.^46^ The Cu L-edge NEXAFS spectra of the Cu/G and CuZn/G samples also present a strong resonance related to the 2p → 3d transition at approximately 931 eV, which is characteristic of Cu(II) species.^46^ This resonance is only minimal for the CuFe/G sample suggesting that the immobilization of Fe produces a reduction of Cu(II) to Cu(I) in the pristine electrodeposited Cu/G. The spectra were fitted using a linear combination analysis (LCA) of reference spectra for metallic Cu, cuprous oxide, and cupric oxide, which shows that the initial structure of the particles is predominantly a cuprous oxide phase.

A closer inspection of the Cu L_3,2_-NEXAFS spectra of these samples (Figure S3a) reveals subtle changes in their electronic structure, attributable to a distinctive electronic effect exerted by the second metal on Cu. Particularly, when compared to Cu/G, the resonances due to the 2p → 3d and 2p → 4p transitions shift to lower photon energy for CuFe/G and CuZn/G, more markedly so for the Fe-containing system. A similar shift of the 2p → 4p was previously observed upon annealing of electrodeposited Cu_2_O nanoparticles and attributed to changes in Cu–O bond length due to the formation of isolated reduced sites in this structure.^47,48^ Moreover, the higher intensity of the Cu(I) resonance for Cu/G is consistent with lower long-range order or more molecular character of Cu–O species in this sample (more terminal OH, less bridged O species). The spectroscopic structural characterization of the as-prepared sample is reported and discussed more extensively in the supporting information (Figures S3 and S4). Therein, the Zn L-, Fe L-, and O K-edges indicate the presence of Zn(II) in CuZn/G and a mixture of Fe(II) and Fe(III) species in CuFe/G. The quantitative elemental analysis by X-ray photoelectron spectroscopy (XPS) (Table S1) also shows Cl impurities in the fresh Cu/G and S impurities in CuZn/G and CuFe/G.

### 2.2 Electrocatalytic Performances in Flow Electrocatalytic Cells

**Figure 2:**
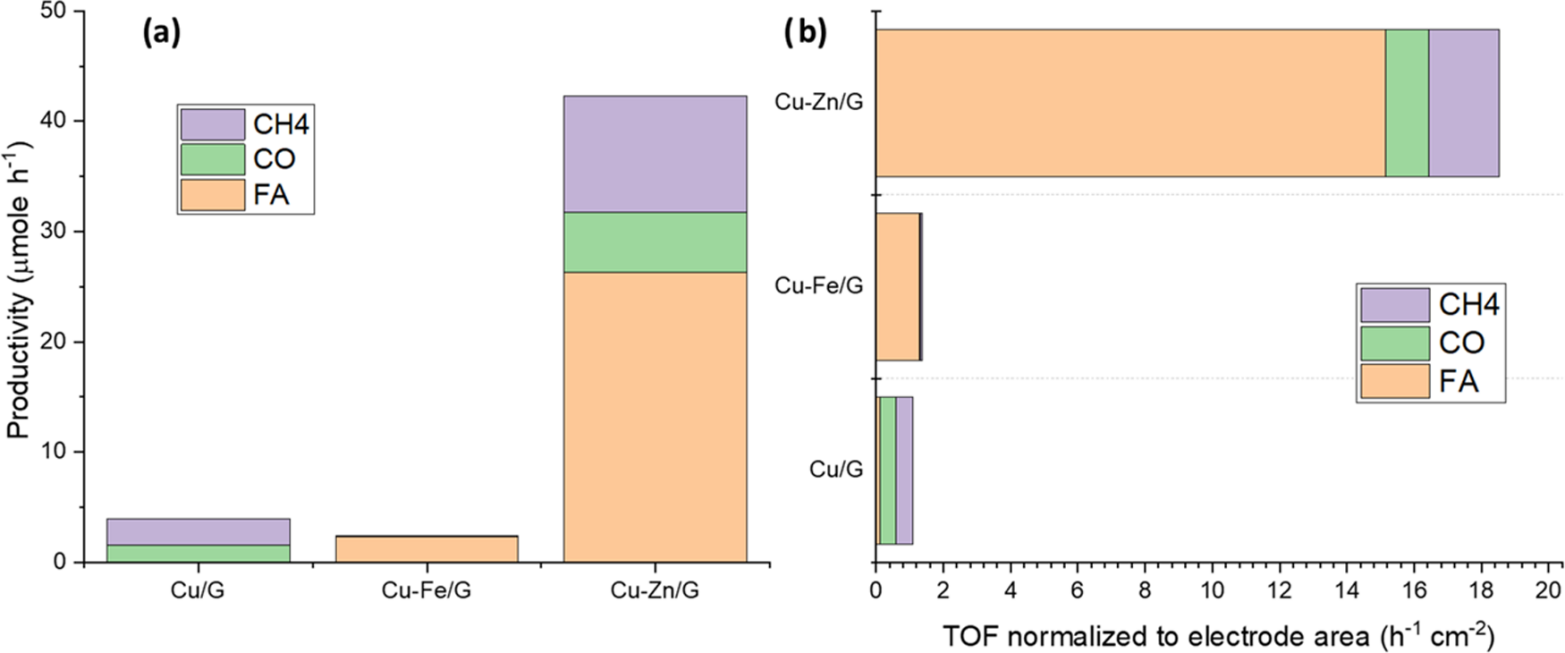
CO_2_RR performances in CO_2_-saturated, 0.1 M KHCO_3_ aqueous solution using the electrochemical device described in the Section 4: (a) production rates for HCOOH, CO, and CH_4_ with Cu/G, CuFe/G, and CuZn/G as electrocatalysts at −2 V vs Ag/AgCl) (−1.38 V vs RHE). (b) Turnover Frequency for HCOOH, CO, and CH_4_ by using Cu/G, CuFe/G, and CuZn/G catalysts at −2 V vs Ag/AgCl (−1.38 vs RHE).

Turning now to the electrocatalytic performance results, Figure 2a shows the production rates (μmol·h^–1^) obtained for Cu/G, CuZn/G, and CuFe/G catalysts at −1.38 V vs reversible hydrogen electrode (RHE) in 0.1 M CO_2_-saturated KHCO_3_. Formic acid (FA) is the main liquid product formed in CO_2_RR tests, with a maximum production rate of 26.5 μmol·h^–1^ for CuZn/G. Other minor liquid CO_2_RR products were methanol, ethanol, isopropanol, and acetic acid, but they were obtained only in low concentrations for all of the electrodeposited Cu catalysts. The main gaseous carbon products are CO and CH_4_, with CuZn/G giving the highest production rates of 5.4 and 10.5 μmol·h^–1^, respectively. CuZn/G gave the best performances in CO_2_RR efficiency, even though the current density was not the highest (15 mA·cm^–2^). The maximum current density was observed for CuFe/G, which produced the largest amount of hydrogen instead. Figure 1b shows the calculated turnover frequency, TOF (g_product_·g_metal_^–1^·h^–1^·cm^–2^), for the reaction products such as formic acid, CO, and CH_4_. Specifically, the TOFs for formic acid, CH_4_, and CO for CuZn/G are higher than for Cu/G, with the TOF for formic acid being 5 orders of magnitude higher than other products.

**Figure 3:**
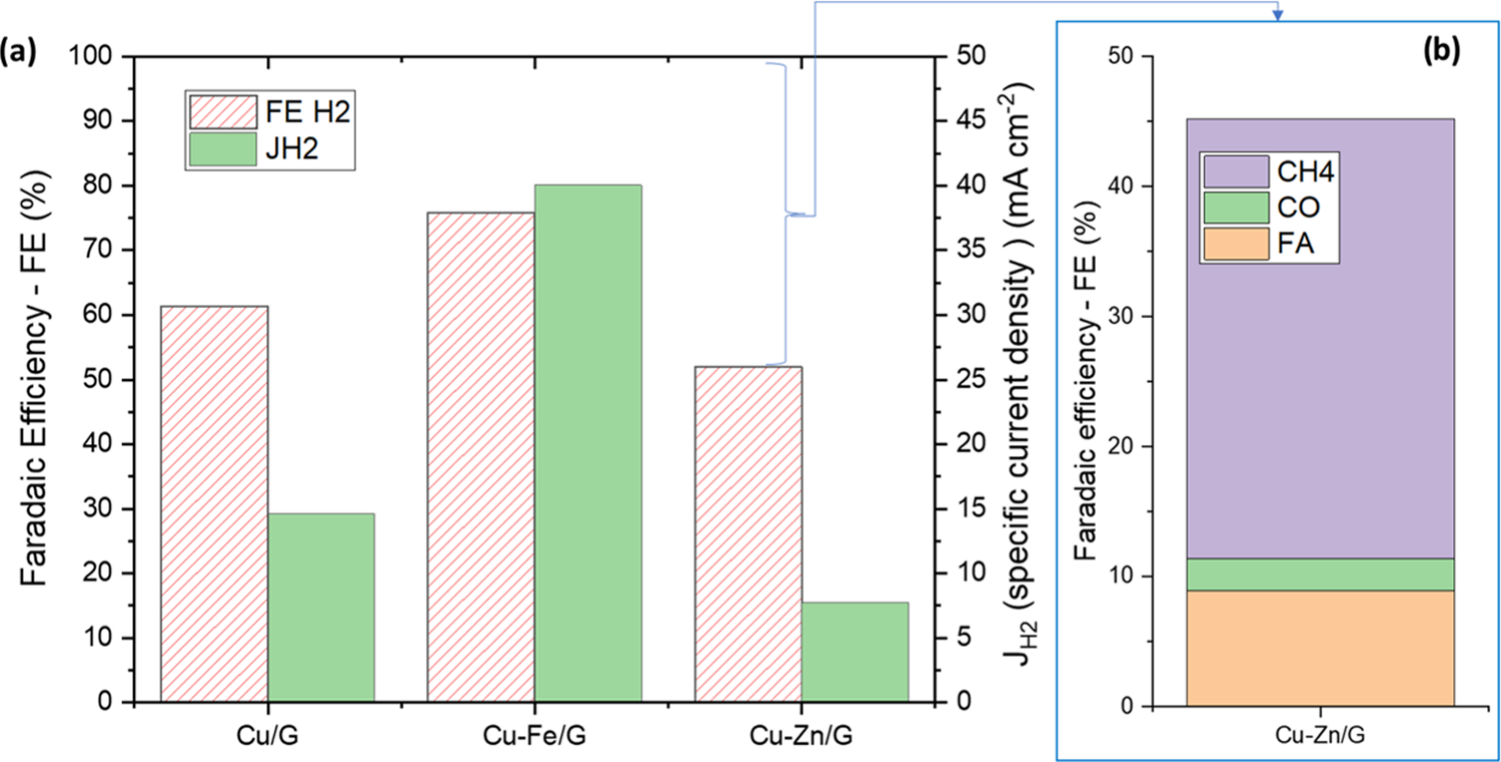
CO_2_RR performances in CO_2_-saturated, 0.1 M KHCO_3_ aqueous solution using the electrochemical device described in the Section 4: (a) Faradaic efficiency (FE H_2_, %) and specific current density (*J*_H_2__, mA·cm^–2^) to hydrogen for Cu/G, Cu–Fe/G, and Cu–Zn/G catalysts at −1.38 V vs RHE. (b) Faradaic efficiency to formic acid (FA), CO, and methane of Cu–Zn/G electrocatalysts.

Figure 3a shows the Faradaic efficiency (FE H_2_) and current density to hydrogen (*J*_H_2__) obtained for Cu/G, CuFe/G, and CuZn/G electrocatalysts at −1.38 V (vs RHE). In Figure 3b, the Faradaic efficiencies for the main products of CO_2_ reduction (formic acid, CO, and methane) are also shown for Cu–Zn/G, which provided a total carbon-based FE of 46.8% against 4.1% and 1.2% for Cu/G and Cu–Fe/G, respectively. The specific current density toward the main reduction products is also reported in Figure S5 for all of the electrocatalysts. The electrocatalytic testing shows that these Cu/G electrocatalysts are only weakly active and selective toward CO_2_RR, while the hydrogen evolution reaction (HER) is the dominant reaction; but they nonetheless demonstrate that it is indeed possible to tune the selectivity toward CO_2_RR by modifying the Cu electronic structure.

CuZn/G shows a largely improved productivity toward CO_2_ electroreduction compared to Cu/G, consistent with the recent literature showing the beneficial effect of Zn.^36,41−43^ The particle morphology in CuZn/G, allowing both (100) and (111) exposed facets, might also be a factor^44^ in the improved performances reported. In terms of product distribution, Cu/G and CuZn/G behave similarly to the Cu nanocubes on C systems previously investigated by Grosse et al.^12^ In contrast, the parasitic HER is favored over CuFe/G; this is expected if an Fe-exposed surface is predominant in this sample, consistent with recent RIXS work on FeCu systems, showing the surface segregation of Fe at cathodic potentials, which is responsible for the HER selectivity.^49^ The formation of formic acid (FA) for this sample could also be attributed to the reactivity of Fe(III)–OH species.^50,51^

The addition of Zn not only enhances the activity but also changes the selectivity by over-quadruplicating the productivity to methane, in addition to forming formic acid (FA) (compare Cu/G and CuZn/G in Figure 2). A similar effect was observed for metallic Cu particles exposing (111) surfaces^52^ as well as due to Zn–Cu alloy formation.^41^ Here, the two aspects cannot be separated since after the Zn impregnation, the formation of (111) surfaces is also observed, and these manifest through a specific Cu electronic structure, which will be described in the following section.

### 2.3 Operando Study under Polarized Conditions

#### 2.3.1 Hard X-ray Spectroscopy Techniques

The bulk structure of the electrode as a function of the applied potential is obtained from the hard X-ray spectroscopic data at the Cu and Zn K-edges.

**Figure 4:**
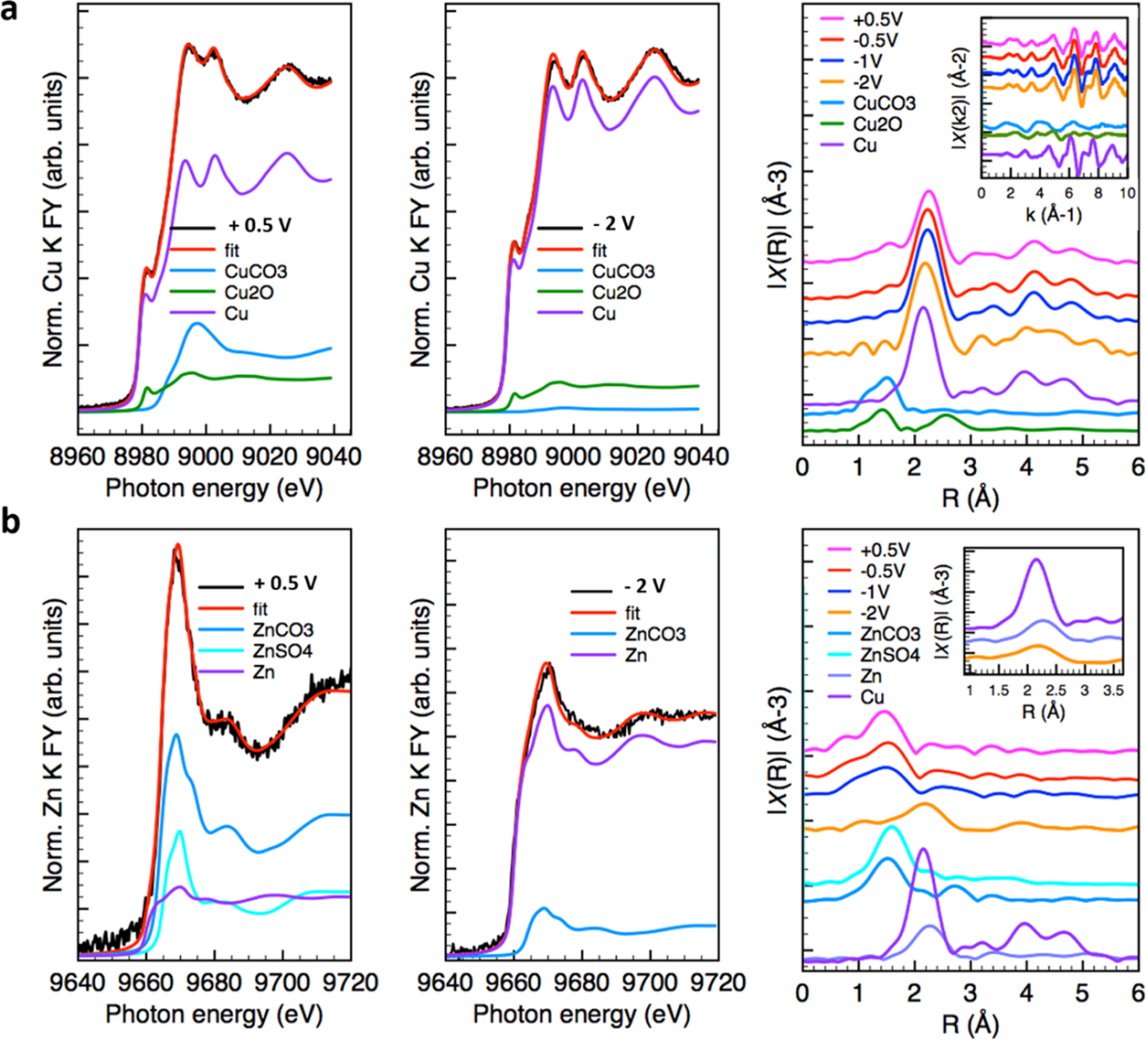
Operando spectroscopic data in 0.1 M CO_2_-saturated KHCO_3_ as a function of potential under potentiostatic control for CuZn/G: (a) Cu K-edge FY XANES and EXAFS FT; (b) Zn K-edge FY XANES and FT EXAFS. The inset in (b) shows the zoom of the second shell signal assigned to metal–metal distances. The order of the experiments is as follows: +0.5, −0.5, −1, and −2 V vs Ag/AgCl.

The Cu K-edge XAFS data for CuZn/G in 0.1 M CO_2_-saturated KHCO_3_ aqueous solution and at open-circuit potential (OCP) in Figure S6 and Table S2 show that the Cu phase undergoes a carbonatation reaction consistently with literature work on Cu single crystals.^52^ The samples differ largely in terms of phase composition. CuCO_3_ is the prevalent phase for Cu/G (ca. 67%), with the remaining fraction being Cu_2_O; in contrast, CuZn/G is mainly composed of a Cu_2_O phase (ca. 90%) and in a minimal part of CuCO_3_ (Table S2). No metallic component was identified in the bulk of the sample under equilibrium conditions with the KHCO_3_ solution. Moreover, at the Zn K-edge, we observe that the Zn(II) sulfate species that are initially present on the fresh CuZn/G sample are converted to carbonates under open-circuit potential. The results of the operando Cu K-edge XAFS study on CuZn/G at selected potentials in potentiostatic control are reported in Figure 4. The results of the potentiodynamic experiment in Table S3 are consistent with the constant potential experiments (Figure 4 and Table 4) and show that at the highest anodic potential investigated (+0.5 V vs Ag/AgCl), the fraction of Cu(II) species in the form of carbonate is substantially increased while at the lowest potential (−2 V), the Cu(0) fraction is the dominant phase, consistent with previous works.^26−29^ As the potential is changed from positive to negative and vice versa (Table S4), a fraction of the metallic copper as high as ca. 70% appears to be unaffected by the applied voltages, at least within the time scale investigated in these experiments. This can be attributed to differing particle morphologies and sizes, with the atoms in the bulk of the NPs remaining metallic, whereas only the interfacial species undergo chemical transformation triggered by the interaction with the liquid electrolyte and the potential. We can also observe that the CuZn/G electrocatalyst still contains Cu(I) species at any voltages. Cu(II) carbonate is also present at cathodic voltages consistent with previous work indicating the formation of a passivating carbonate layer for a high local concentration of bicarbonate species at the interface.^27−29^ Concerning the Zn speciation, the spectrum collected at −0.5 V vs Ag/AgCl (Figure 4b) shows Zn metal as well as Zn sulfate, but the dominant phase is Zn carbonate. At more negative potentials, a progressive reduction of the Zn sulfate to metallic Zn occurs first, followed by the reduction of Zn carbonate only at −2 V vs Ag/AgCl. This implies that S species are not stable under cathodic potential and are replaced very rapidly, already after a few stabilization cyclic voltammetry (CV) cycles (Table S4), whereas Zn carbonate is only reduced at the potential with the highest CO_2_RR rate. An important finding is obtained from the Zn K-edge EXAFS analysis (Figure 4b). In particular, it was observed that at very negative potentials, Zn is alloyed into the Cu structure as seen by the reduction of the metal–metal bond length at −2 V vs Ag/AgCl, when compared to the Zn–Zn distance for a reference Zn metal EXAFS spectrum. No segregated metallic Zn phase is observed. The Cu K-edge operando study on the Cu/G sample under potentiostatic control (Figure S7 and Table S5) consistently shows that at −2 V vs Ag/AgCl, besides the main metallic Cu phase, part of the electrocatalyst is still present as Cu_2_O and Cu carbonate. The effect of Zn can be clearly seen by comparing the results of linear combination fitting (LCF) analysis on both X-ray absorption near-edge spectroscopy (XANES) and EXAFS (0–13.5 Å^–1^) for CuZn/G (Table S3) and Cu/G (Table S5). Accordingly, the oxidation of Cu happens for Cu/G at more positive potentials reaching a higher fraction of Cu(II) in the form of carbonate compared to CuZn/G. The progressive reduction to Cu^0^ is observed at more negative potentials for Cu/G through the intermediate formation of Cu_2_O species.

The SEM images in Figure S8 for CuZn/G (a) and Cu/G (b) after the operando Cu K-edge study show crystallites with spike-like morphologies characterized by a potassium accumulation. These can be attributed to the electrocatalytically inactive mixed carbonate phase, consistent with the Cu K-edge analysis. In general, throughout the reaction, Cu(I)–O species are more stable and present in a significantly higher fraction at any conditions for the CuZn/G sample with respect to Cu/G, suggesting that Zn stabilizes Cu(I) species against both oxidation and reduction.

#### 2.3.2 Soft X-ray Spectroscopy

Turning to the catalytic performances obtained with the in situ electrochemical cell for soft X-ray spectroscopic studies, the average currents exchanged during the chronoamperometry experiment under steady-state conditions at each voltage applied are summarized in Table S6. Accordingly, the activity is higher for CuZn/G than for Cu/G, qualitatively consistent with the experiments in Figures 2 and 3; CuFe/G appears to be the most active when the potential from OCP is set directly to CO_2_RR conditions (CuFe/G-1 in Table S6); in contrast, when the potential is lowered stepwise (CuFe/G-2 in Table S6), the performances of CuFe/G at potentials of relevance for CO_2_RR worsen and become unselective, similar to the flow cell measurements in Section 2.2. The online gas analysis by mass spectrometry in Figure S9 clearly indicates that for all of the electrocatalysts, the production of H_2_ occurs at potentials below −1.5 V vs Ag/AgCl, while at the same time, CO_2_ is consumed. The identification of CO_2_ reduction products from the mass spectra during the in situ experiments is not always unambiguous. It must also be noted that the gaseous products escape from the reaction environment (the compartment containing the stagnant liquid electrolyte, see Figure 6 in the experimental part) through holes in the graphene layer; it is expected that the graphene layer and the dimension of the holes will certainly have an influence on the lateral interactions between adsorbed molecules and thus on the gases produced and their diffusion from the reaction environment to the evacuated chamber where they are detected. Thus, any analysis is limited to a qualitative assessment. It is possible to infer that the increase of the *m*/*z* = 31 mass fragment below −1.8 V vs Ag/AgCl is unequivocally due to methanol formation, which is favored when the water partial pressure increases, consistent with previous work;^47^ the *m*/*z* = 15 signal could be attributed to the formation of methane, which is expected for these electrocatalysts (Figures 2 and 3). Figure S9d reported the mass spectrum measured in the experiment on CuFe/G in which, from OCP, the potential for CO_2_RR was directly applied without the sequential lowering (FeCu/G-1 in Table S6). In this case, we also observe the production of the mass fragment *m*/*z* = 29, which is the most intense fragment for formic acid, consistent with the data reported in Figures 2 and 3; this fragment could also be related to C–C containing hydrocarbons, consistent with previous work on Fe carbon systems.^50,51^ In general, the mass fragments observed are similar for the three samples indicating that the significant product differentiation observed on the bench scale experiments occurs at a longer reaction time than those explored during this in situ study, while in a CO_2_RR-activated electrode state, methanol is one of the main products observed in situ for all electrocatalysts due to the more hydrophilic surface of the systems within the time scale and conditions of the in situ experiments. It must also be pointed out that the CuFe/G system showed the poorest stability, with performances deteriorating rapidly even in the time frame of the in situ experiment and particularly when the potential is lowered stepwise from OCP to −2 V vs Ag/AgCl (Table S6). The MS under this condition shows only H_2_ formation, although CO_2_ is consumed (Figure S10a). It will be discussed later that the spectroscopic results of this sample in a CO_2_ reduction active state and inactive state are significantly different.

**Figure 5:**
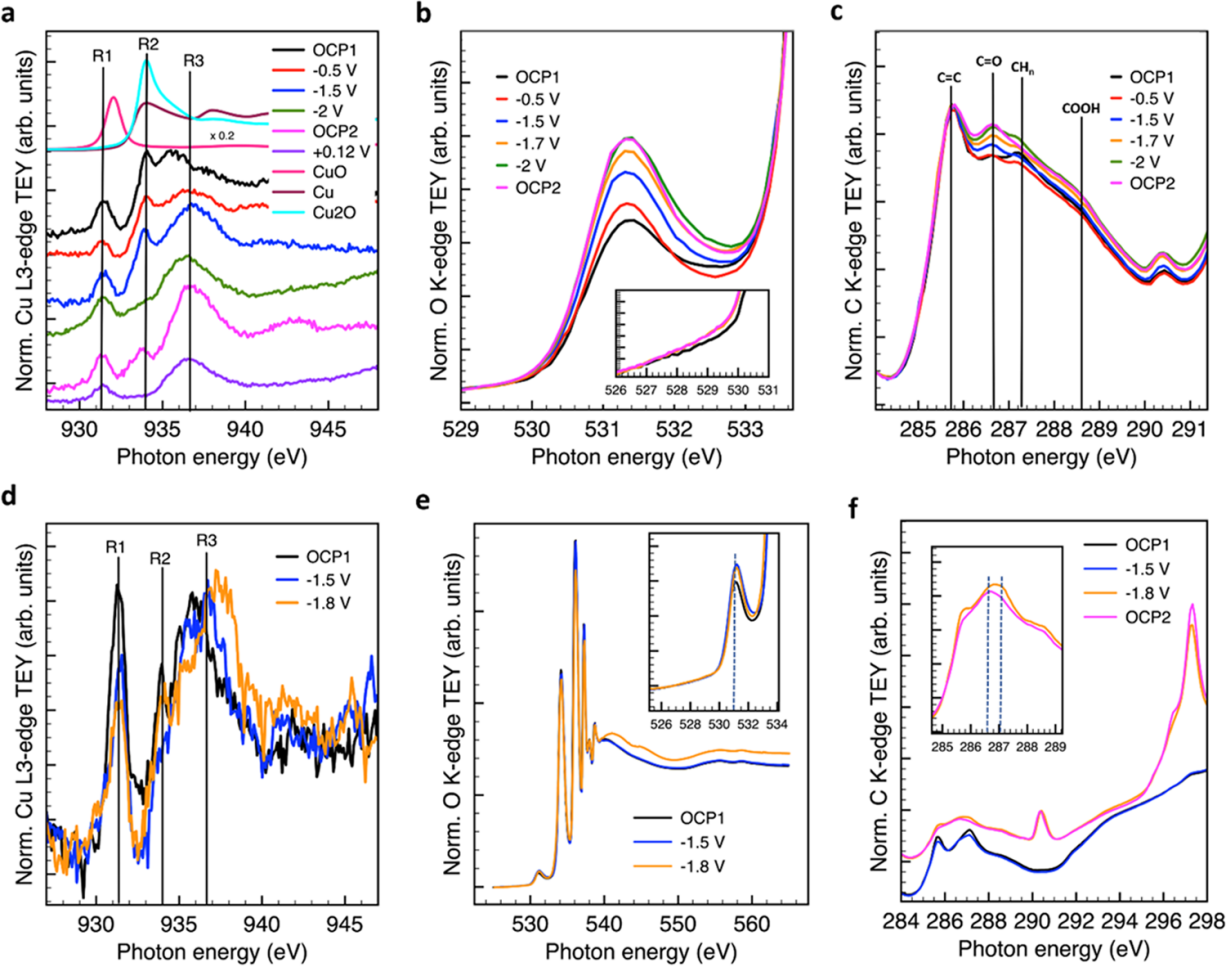
Soft X-ray in situ NEXAFS data under a stagnant KHCO_3_ electrolyte at different voltages as indicated. Chronoamperometry measurements for CuZn/G were performed in the following order: open-circuit potential (OCP), −0.5, −1, −1.5, −1.7, −2 V, OCP2: Cu L_3_-edge (a), O K-edge (b), and C K-edge (c); chronoamperometry measurements for CuFe/G performed in the following order: OCP1; −1.5, −1.7, −1.8 V, OCP2: Cu L_3_-edge (d), O K-edge (e), and C K-edge (f).

Figure 5a–c shows the TEY Cu L_3_-edge, O K-edge, and C K-edge NEXAFS spectra measured in situ for CuZn/G at different voltages vs Ag/AgCl. For comparison, Figure 5d–f reports the in situ data during constant potential measurements on CuFe/G in its most active state. The same investigation was performed on Cu/G and is reported in Figure S11.

The CuZn/G sample under OCP is characterized by a distribution of species identified by three resonances, namely, R1, R2, and R3. As mentioned earlier, the resonance R1 at ca. 931. eV is due to a Cu 2p → 3d transition typical for Cu(II) species.^46^ The sharp R2 resonance at approximately 934 eV is due to a Cu 2p → 4s transition and indicates the presence of Cu(I)–O species consistently with the reference spectrum for cuprous oxide. Moreover, in agreement with the Cu L_3_-edge reference spectrum for metallic Cu, face-centered cubic (fcc) metallic Cu is characterized by a three-peak pattern above the fermi energy explained by a p → d dominant contribution (although the Cu d band is full, s–p–d rehybridization results in unoccupied densities of states of d-character above the Fermi level).^53^ When comparing the spectrum of CuZn/G (Figure 5a) at OCP with the spectra of the Cu_2_O and Cu^0^ references in the same figure or with the fresh sample in UHV in Figure 1, it appears evident in the region above the edge that a mixture of metallic Cu (resonance at 943 eV) and Cu(I) is present at this condition together with Cu(II). The metallic Cu is expected to be formed during the CV cycles preceding the constant potential investigation reported in Figure S9a and represents the more bulk component of the Cu electrode. However, in contrast to the fresh samples, a meaningful LCA was not possible here using the reference spectra, which suggest a different chemical environment of the adsorbed Cu atoms compared to standard samples in the fresh state.

At −0.5 V vs Ag/AgCl, only minor changes in the Cu L-edge feature are visible for CuZn/G in Figure 5, indicating an ongoing reduction. The presence of Cu(II) at this potential was observed for Cu systems in high-concentration bicarbonate solutions due to the formation of Cu carbonate essentially representing a loss of electroactive Cu.^27−29^ Cu(II) species could also result from a potential-driven dissolution and redeposition to form roughened surfaces as shown in a previous transmission electron microscopy (TEM) study.^54^

At −1.5 V vs Ag/AgCl (Figure 5a), another resonance R3 becomes more intense; note that at this potential, H_2_ evolution and CO_2_ consumption are observed to occur simultaneously, as shown in Figure S9c. At −2 V, the CO_2_RR rate is higher, and the involved surface dynamics consist of consumption of the Cu(I) species (R2) and a change in the shape of the R3 resonance with a slight shift toward lower energy, suggesting that many components contribute to the broad R3 feature. On returning to OCP (OCP2), R2 reforms (Cu(I) species). Under this condition, not only is the fine structure of the metallic component visible (resonance at 943 eV), but the R3 resonance becomes very intense and slightly shifts to higher energy. Under a more positive potential, the Cu(I) species is completely converted into Cu(II), as expected from thermodynamic considerations, thus confirming the well-functioning of the cell; the spectrum is now dominated by the R1 and the R3 resonance.

Figure 5b,c shows the O K-edge and C K-edge spectra measured subsequently to the Cu L-edges at the different voltages, respectively. Considering the method used to detect the signal, the C and O K-edges are a convolution of species from the graphitic support, the graphene overlayer, and the CO_2_-adsorbed species; thus, precise analysis is complicated. Nevertheless, we present herein a comparative analysis. We focus on the pre-edge regions because, above the edge, the spectra are dominated by the signal of CO_2_ and water in the gas phase: note that the broad resonance in the region above 539 eV in the O K-edge confirms the formation of a liquid film (shown in Figure 5e for the CuFe/G, as an example). With lowering the potential, the intensity of resonance at 531.4 eV increases, which is attributed to π* resonances for OH or methoxy species.^55^ Subsequently, by allowing the electrode to equilibrate at OCP2, it was possible to discover that the pre-edge π* resonance is broader at −2 V, suggesting the presence of another species at around 532 eV formed under CO_2_RR conditions. At this photon energy, formate species were found,^55−57^ consistent with the reactivity data for this sample (Figures 2 and 3). No resonances are seen below 530 eV, indicating the absence of atomic oxygen or anionic molecular oxygen species. The C K-edge NEXAFS spectrum, normalized to the C 1s → π* resonance of the C=C bond for graphitic carbon at 285.7 eV, shows the increase of the resonance at 286.7 and 288.6 eV with lowering the potential, attributed to C=O bonds and COOH bonds, respectively.^56^ These species are also observed upon the interaction of CO_2_ on Fe–OOH surfaces in 0.1–0.25 mbar CO_2_.^50^ Similar reference experiments of the adsorption of CO_2_ on the set of samples investigated here confirm that indeed these species form (Figure S10b) the former one (C=O bonds) in very small amounts compared to the case of the polarized surface at any voltages as shown in Figure 5. The opposite is true for COOH species.

An additional resonance at 287.3 eV is also observed at −2 V, which could be assigned to C–H bonds.^56^ This resonance increases with lowering the potential, similar to the other resonances. However, in contrast to the other resonances, it decreases again when the voltage from −2 V is allowed to equilibrate to the open-circuit value, suggesting a voltage-dependent formation and, thus, a direct correlation with the reduction reaction and the products evolved. The average current measured under steady-state conditions at each potential (Table S6), as well as the mass spectrum (Figure S9c), shows that CO_2_RR is mostly favored at −2 V under these conditions, whereas at higher (less negative) voltages, CO_2_ is consumed. These dynamics can be rationalized on the basis of an accumulation of adsorbates between OCP and −1.5 V, whose nature is described by specific resonances in the absorption spectra as discussed above (here mostly C=O species, whereas COOH is the main species under nonpolarized conditions, see Figure S10).

At OCP, the Cu L_3_-edge of Cu/G in Figure S11a is now predominantly characterized by Cu(II) species; for this reason, any changes in the edge resonances during the cathodic polarization are difficult to extrapolate from these measurements. Nevertheless, we note that the reduction of the Cu(II) and Cu(I) intensities and the increase of the intensity above the edge at −2 V vs Ag/AgCl is consistent with a small fraction of the sample undergoing reduction to Cu(0). The excessive Cu(II) formation might indicate an interaction with the liquid electrolyte leading to the formation of a mixed Cu/K carbonate and thus to a progressive loss of the electroactive surface. The extent of this process depends on the local concentration of the bicarbonate solution^27−29^ that in this cell arrangement of the stagnant liquid film could be favored as well as on the higher propensity of this sample to undergo dissolution due to the more roughened particles’ morphology and its higher molecular character and reduced long-range order (Figure 1a) when compared with CuZn/G. Consistently, we also note at the Cu K-edge that the carbonate formation is more extended for Cu/G than CuZn/G already at OCP (Figure S7 and Table S2). In the case of CuZn/G, we assume that the larger cuboctahedrons allow a lower Cu exposure, and consequently, the dominant Cu_2_O phase in the Cu K-edge remains mostly unaltered under OCP or becomes metallic at lower voltages. Due to the higher surface sensitivity of the Cu L-edge spectra, compared with the Cu K-edge spectra, it could be expected that Cu(II) dominates the signal. A beam-induced oxidation of Cu in an alkaline solution was observed with higher brilliance sources than the one used in our study, which overwhelmed the signal of the electrochemical reduction for 7 nm Cu nanoparticles, and to a much smaller extent for 18 nm Cu nanoparticles.^58^

The O K-edge in Figure S11b shows a much narrower resonance at 531.2 eV (characteristic of OH or methoxy species^55^). Interestingly, a component at 528.5 eV either indicates the formation of anionic molecular or atomic O species on Cu or is related to alkali metal oxides; this resonance is found at the same energy as the pre-edge peak in doped cuprates and observed under anodic conditions over Ir oxides.^59^ This raises the question as to whether these oxygen species are related to the Cu(II) formation and might well be the manifestation of a dissolution process leading to the particle surface roughening,^54^ possibly also caused by the beam. Indeed, beam-induced oxidation is considered to be caused by the generation of O-radicals due to water photolysis. However, under irradiation, we do not observe consistently periodic current spikes due to the competitive cathodic reduction and oxidative beam effect,^58^ but current fluctuations are more pronounced at higher currents when gas evolution disturbs the potential control (Figure S11e). Moreover, the resonance associated with these O species forms under cathodic polarization (ca. −1.5 to 1.7 V) when the part of the Cu(II) species is actually reduced, and it becomes sharper (more molecular) at OCP2. When going from OCP to −0.5 V, the C K-edge spectrum in Figure S11c shows a more pronounced increase of the resonances above the C=C peak (285.75 eV) for Cu/G than in the case of CuZn/G (Figure 5c), which suggests a larger population of adsorbed species as for a thicker liquid film or higher electrolyte-exposed surface area. In particular, the C K-edge in Figure S11c is characterized by an increase in the intensity of the resonance at 286.7 eV (characteristic of the C=O double bond) with further lowering the potential, whereas the intensities of the resonances at higher energies are not changing significantly with the potential; if one considers that the resonance at 286.7 eV is associated with the C=O double bond of the carbonyl group in adsorbed species, whereas the resonance at higher energy (above 287 eV) is related to single C–H bonds,^56^ it suggests that the interfacial speciation reflects the different selectivity to CH_4_ observed for these systems (Figures 2 and 3), with CuZn/G showing much higher productivity toward hydrogenated molecules whereas Cu/G is more selective toward CO. Thus, while the beam-induced oxygen species radicals cannot be excluded, the formation of the oxygen species associated with the resonance at 528.5 eV seems to be a manifestation of a different mechanism operating on the Cu/G system compared to that on the bimetallic systems, leading to the formation of CO. This postulation requires further studies.

Formate species are generally not changing much for CuZn/G and Cu/G.

The comparison with the dynamics occurring over the CuFe/G system is very useful to highlight the distinctive role of the heteroatom in modifying the Cu electronic structure, hence tailoring the product selectivity and enabling a more active CO_2_RR state. We first discuss the results obtained during the experiment in which the electrocatalyst from OCP was directly polarized at sufficiently low voltages for the CO_2_RR to occur. Under OCP (Figure 5d), CuFe/G is characterized in the Cu L-edge spectrum by the R1 resonance attributed to Cu(II) species whose intensity decreases with lowering the potential. At −1.5 V, the R1 component is partially consumed. Lowering the potential further to −1.8 V leads to a relatively higher cathodic current density and production of H_2_ in the mass spectrum (Figure S9d). Interestingly, Cu(I) species are stable under CO_2_RR, whereas the R3 resonance shift to higher energy indicates a different ligand environment than that in the case of CuZn/G as shown in Figure 5a. The higher abundance of Cu(I) under this condition is correlated with the good performances of these electrocatalysts similar to CuZn/G. The O K-edge spectra at the potentials investigated show the formation of the same species at 531.2 eV (OH^–^ and methoxy group^55^) as for CuZn/G. We note that the water transport through the membrane significantly increases at −1.8 V, as shown in Figure 5e, by the pronounced resonance above 539 eV due to liquid water^59^ consistent with an increased wetting of the electrocatalyst that explains the high current recorded under this condition. The C K-edge also indicates that the electrolyte transfer is favored at −1.8 V with the consequent increase of the K L-edges signal (resonance at approx. 297.5 eV in Figure 5f). The C K-edge also shows the formation of a component at ca. 287 eV; we suggest that this is still indicative of the presence of a C=O-containing species. The SEM image of this sample (Figure S12) after the in situ experiment of Figure 5d–f shows that the bimodal particle dimensionality is preserved with smaller CuFe particles sparse on the C support as well as larger predominantly Fe agglomerates.

A prolonged potential controlled experiment on a chemically identical sample and stepwise lowering of the voltage shows very different dynamics (Figure S10b) as well as a lower current transferred while only H_2_ is observed (Figure S10a).

The R2 resonance is not as evident for this sample at OCP as in the case of CuZn/G reported in Figure 5a. The formation of metallic Cu during the preceding CV explains the spectral features observed above the edge. In fact, any change in the d electron count at the Cu site due to the interaction with the Fe or Zn atoms (e.g., alloying) would have a noticeable effect on the Cu L_3_-edge NEXAFS resonances.^46^ For example, it has been reported that alloying of Cu with Au causes the first resonance to shift to lower energy and increase in intensity, with these changes becoming more pronounced for higher Cu dilution.^48^ Also, a reduction of the intensity of the second and the third resonance was observed upon alloying and tentatively explained as a localization of the d-electrons. In the case of alloying with a less noble metal than Cu, such as Ni, the Cu L-edge shifts progressively to higher photon energy as Cu becomes more diluted.^60^ We postulate a similar effect should be exerted by Fe on Cu. Moreover, for Cu/Fe-multilayers,^61^ it was shown that when Fe is the abundant element, the alloy adopts a body-centered cubic (bcc) structure, which is characterized by two-peak features above the Fermi level. A similar spectrum was also observed for a two-dimensional (2-D) oxide in weak coupling to the underlying metal phase.^62^ A metallic phase is indeed expected at negative potentials, consistent with bulk-sensitive operando studies.^27−29,63^ At more negative potentials, the CuFe/G system does not show the resonance typical for Cu(I) species; it also differs from the CuZn/G in that the R3 resonance is not formed under CO_2_RR conditions. It follows that no Cu–CO_2_-adsorbate species are present in this sample under these conditions. We suggest that this is a consequence of the distribution of the two elements at the interface, with only the CuZn system enabling stable electrolyte-exposed atomically dispersed Cu(I) species available for chemisorption and more exposed Cu domains under reducing potentials and at longer reaction times. In fact, the homogeneous distribution of both metals in CuZn/G with no segregated metallic Zn phase was confirmed on the sample under OCP by EXAFS analysis at the Cu K- and Zn K-edges (Figure S6 and Table S2), which led to the alloy formation under CO_2_RR conditions (Figure 4).

In contrast, Fe would tend to segregate to the surface, preventing the exposure of Cu sites, as recently evidenced by a RIXS study.^49^ This description would explain the reactivity observed for this sample which produces predominantly H_2_ and HCOOH (Figure S10), both produced on Fe surfaces.

##### 2.3.2.1 Simulation of Cu L-Edge NEXAFS Spectra

For a comprehensive description of the spectroscopic data, it is important to understand the origin of the R3 resonance at ca. 935.5–936.7 eV.

The nature of the R3 resonance is poorly documented in the literature. In this region, 2p → 4s transitions are usually reported.^43,64^ A model involving the localization of an electron–hole on the supporting ligands was presented to describe the observed reduction of the 2p → 3d intensity and the increase of the 2p → 4s and shake-up satellites at 937 eV for Cu(II) and Cu(I) complexes.^65^ This is consistent with the inverted bonding description of coordination complexes suggested by Sarangi et al.,^66^ in which the lowest unoccupied molecular orbital (LUMO) ismainly dominated by holes in the ligands. Changes in ligand field due to different coordination geometry and bonding distance of the ligands might also have a similar effect on the edge position.^67^ A shift of the Cu(I) resonances to similar ranges was observed for the absorption of various molecules on Cu(I)-MFU metal–organic framework^68^ and for CO on high configurational entropy oxides Mg_0.2_Co_0.2_Ni_0.2_Cu_0.2_Zn_0.2_O.^69^ Such a shift was discussed in terms of π-backbonding from metal orbitals with Cu d-character to unoccupied ligand orbitals for various Cu(I)-ligated species such as CO and N_2_, including ligands with predominantly ligand-to-metal σ-donation such as H_2_ and NH_3_. The energies of the transitions were found to correlate well with the energy levels of the molecule adsorbed, whereas the transition intensities were proportional to the binding energies of the guest molecule. Accordingly, the resonance emerges at 935.3, 935.9, and 936.3 eV for CO-, N_2_-, and H_2_-dosed samples, respectively. The resonance was shifted at an even higher photon energy for NH_3_.^68^

Based on all of these considerations, we assume that the R3 resonance could be due to a ligated surface Cu(I) species derived from the adsorption of anions or CO_2_ reduction intermediates in the electrolyte, exerting an electron-withdrawing effect on the metal center resulting from the electron transfer process that leads to the formation of the CO_2_-reduction product.

We used computational methods to understand the nature of the spectral features of the Cu L-edges and the influence of the additional elements on the electronic state of Cu. Method validation against reference materials Cu, Cu_2_O, and CuO is reported in Figure S13.

We created a 3 × 3 × 3 supercell of Cu_2_O and Cu(OH)_2_ and introduced substitutional impurities of Zn or Fe in randomly selected Cu positions, then relaxing the structures up to an energy change of 10^–6^ eV·atom^–1^ using the CASTEP code as discussed above. The Mulliken population analysis, shown in Tables S7 and S8, was performed on the simulated electronic ground state.

These structures were used for the simulation of Cu–L absorption edges. Figure S14 shows the intensity in the transition from 2p to an admixture of 3d and 4s states above the Fermi energy. Such a trend clarifies the electron-donor nature of both Zn and Fe dopants, providing electrons that are redistributed to either the Cu 3d or 4s orbitals, thus decreasing the transition matrix elements for the lowest unoccupied orbitals and consequently the transition probability and spectral intensity. Following the suggested electronic effect exerted by the ligand on the metal center,^70^ we consider a simplified ionic model in which charge separation occurs between the metal and the ligand during the electron transfer process and explore the effect of such ionization on the L-edge spectra. Such a model would well explain the first coupling of ligands and their desorption due to the cathodic current and as a result of the filling up of the unoccupied orbitals weakening the Me–L bond.

The Cu_2_O model system used here exploits a plane-wave simulation in the solid state; thereby, it is subjected to the rearrangement of charges in the self-consistent calculation. We progressively positively charged the structure leading to a change in the Mulliken charge of Cu (Figure S15).

For positively charged Cu, our study shows that the XANES features due to 4s states, originally localized 2 eV above the Fermi energy, migrate to higher energies. Simultaneously, Cu d-states become increasingly available, leading to a sharp resonance localized near the Fermi energy. The evolution of the spectra is consistent with the effect observed in the operando experiments.

#### 2.3.3 Summary of the Operando Spectroscopic Data

Tables 1 and 2 summarize the spectral features observed at the different voltages by operando spectroscopies and their structural assignment. In Table 1, we compare CuZn/G with Cu/G, which is characterized by a relatively higher current density (Table S6); in Table 2, we compare CuFe/G under CO_2_RR selective conditions with the same system under H_2_ evolution selective conditions.

**Table 1:** Summary of Operando Data for CuZn/G and Cu/G^a^

	interface			bulk	
*E*/V vs Ag/AgCl	Cu L (eV)	C K (eV)	O K (eV)	Cu K (eV)	Zn K (eV)
CuZn/G					
OCP1	931.3, Cu(II)	285.75, C=C	531.3, OH		
	934, Cu(I)–O/Cu(0)	286.65, C=O			
	935.5, Cu(I)–L	287.25, CH*_n_*			
–0.5	931.3, Cu(II) ↓	287.25, CH*_n_* ↓	531.3, OH ↑	Cu (84.4)	Zn (12%)
	934, Cu(0) ↑			Cu_2_O (11%)	ZnCO_3_ (72%)
	936, Cu(I)–L ↑			CuCO_3_ (4.6%)	ZnSO_4_ (16%)
–1.5^b^	936.7, Cu(I)–L ↑	286.65, C=O ↑	531.3, OH ↑	Cu (89%)	Zn (28%)
		287.25, CH*_n_* ↑		Cu_2_O (10%)	ZnCO_3_ (67%)
				CuCO_3_ (1%)	ZnSO_4_ (5%)
–2	934, Cu(I)–O ↓	286.65, C=O ↑	531.3, OH ↑	Cu (88.5%)	Zn (86%)
	935.5, Cu(I)–L ↑	287.25, CH*_n_* ↑	532.3, OH ↑	Cu_2_O (11%)	ZnCO_3_ (14%)
				CuCO_3_ (0.5%)	
OCP2	934, Cu(I)–O ↑	287.25, CH*_n_* ↓	532.3, OH ↓		
	936.7, Cu(I)–L ↑				
Cu/G					
OCP1	931.3, Cu(II)	285.75, C=C	531.3, OH		
	934, Cu(I)–O/Cu(0)	286.65, C=O			
		287.25, CH*_n_*			
–0.5	931.3, Cu(II) ↓	286.6, C=O ↑	531.3, OH ↑		
	934, Cu(0) ↑	287.25, CH*_n_* ↑			
–1.5	931.3, Cu(II) ↓	286.6, C=O ↑	531.3, OH ↑		
	934, Cu(0) ↑		528–529, ↑		
–2	931.3, Cu(II) ↓	286.6, C=O ↑	531.3, OH ↑	Cu (73.2%)	
	934, Cu(0) ↑			Cu_2_O (13.6%)	
				CuCO_3_ (13.1%)	
OCP2	931.3, Cu(II) ↑	286.6, C=O ↑	531.3, OH ↑		
	934, Cu(I)–O ↑	287.25, CH*_n_* ↓			

a At the interfacial region, all of the relevant spectral features are listed at the OCP, whereas only the species undergoing changes are listed at the other potentials, following the order of the experiments. The arrow indicates the qualitative trend for each species, which is oriented upward for an intensity increase and downward for an intensity decrease. In bulk, the quantitative results of the linear combination analysis are reported.

b The Cu K- and Zn K-edges for this data set were measured at −1 V.

**Table 2:** Summary of Operando Data for CuFe/G in the CO_2_RR Selective and HER Selective States^a^

CuFe/G _CO_2_RR selective state			
OCP1	931.3, Cu(II)	285.75, C=C	531, OH
	934, Cu(I)–O/Cu(0)	286.65, C=O	
	935.5, Cu(I)–L	287.25, CH*_n_*	
–1.5	931.3, Cu(II) ↓	286.6, C=O ↑	531, OH ↑
	934, Cu(I)–O ↓		
	936.7, Cu(I)–L ↑		
–1.8	931.3, Cu(II) ↓	286.6, C=O ↑	
	934, Cu(I)–O ↓	287.25, CH*_n_* ↑	
	935.5, Cu(I)–L ↓		
	937.5, Cu(I)–L ↑		

CuFe/G _HER selective state			
OCP1	931.3, Cu(II)	n.d.	n.d.
	934, Cu(I)–O/Cu(0)		
	936.7, Cu(I)–L		
–1.5	931.3, Cu(II) ↑	n.d.	n.d.
	934, Cu(I)–O ↓		
	935.3, Cu(I)–L ↑		
–2	no changes	n.d.	n.d.
+0.15	935.3, Cu(I)–L ↓	n.d.	n.d.
	936.5, Cu(I)–L ↑		

a At the interfacial region, all of the relevant spectral features are listed at the OCP, whereas only the species undergoing changes are listed at the other corresponding potential. The arrow indicates the qualitative trend for each species, which is oriented upward for an intensity increase and downward for an intensity decrease.

We can assume that under OCP, apart from the Cu(I)–O feature at 934 eV in the near-surface, the active CuZn/G surface is populated by ligands coordinated to the Cu(I) sites and appearing at ca. 935.5 eV. The resonance broadens and its maximum shifts to even higher energy (936.7 eV) at more negative voltages. In analogy to previous works,^68^ this could be due to the overlap of ligands of different natures, including CO and H_2_ molecules; the latter one is calculated at relatively higher energy than CO. Species with a higher withdrawing effect on Cu(I) will lead to an upshift of the resonance (described in Section 2.3.2.1), as effectively inducing a higher positive charge on Cu, as expected during a pseudo-capacitive inner sphere ligand reduction step. In the condition of observed evolution of CO_2_RR products for CuZn/G (−2 V), we observe the consumption of the Cu(I)–O species, whereas the broad R3 resonance shift back slightly to a lower value. We can assume that this is due to a large population of these species described by overlapping resonances with a maximum peak at 936.7 eV, which is being reduced due to the CO_2_RR (to form methanol and methane) or the parasitic HER; the reduction of one of the species contributing to the R3 resonance leads to an apparent shift. Adsorbed CO species are still present together with C–H containing species that are formed under these conditions, as seen in the C K-edge at 287.3 eV; therefore, these must be contributing to the remaining part of R3. These interfacial transformations occur on a bulk structure characterized by a dominant CuZn alloy, with a small percentage of the Cu_2_O phase and Cu and Zn carbonates. The reformation of the R3 at 936.7 eV at OCP2 suggests that this resonance might be due also to species such as a poorly reduced CO_2_-derived ligand, including carbonate species.

In the case of Cu/G, we see a predominantly Cu(II) exposed surface ineffective for CO_2_ reduction, consistent with a generally lower activity observed for this sample. This is also consistent with the larger fraction of carbonate species seen at any condition for this system. While under in situ conditions, the formation of methanol is also observed for this system, the predominant CO productivity under the extended experiments in Figures 2 and 3 indicates a poorer availability of H/e^–^ couples under the latter conditions. This is consistent with the dominant C=O resonance in the C K-edge NEXAFS spectra for this sample, indicating a large population of chemisorbed CO (Figure S11). In the CuFe/G system under active conditions, the shift of the R3 resonance to higher values and the reformation of Cu(I) under the condition of the evolution of CO_2_RR products would be consistent with a higher turnover of desorption of reaction products triggered by H species. The relevance of our simulation stems from the fact that these adsorbed species on Cu are found with a similar shift as Cu ligands containing a localized electron–hole in the valence state, as our simulations have proved, which suggests the high reactivity of these toward coupling reaction and the formation of reaction products as a consequence of a M–L σ-bond weakening triggered by the cathodic current on the electrode. Specifically, the activated C–O ligand must be in a chemical environment that supplies H^+^/e^–^ couples for the formation of methane and methanol. These Cu(I) species are especially stabilized on CuZn/G. In the case of CuFe/G, we initially observe irreducible Cu(I) species under CO_2_RR and CO- and H-derived adsorbates on Cu (Cu L-edge in Figure 5d), whereas C species are found at a lower energy in the C K-edge in Figure 5f, compared to CuZn/G. The peak in the C K-edge is shifted to lower energy indicating a chemical configuration closer to C=O than to C–H species, such as a HC=O species (consistently with the evolution of the fragment *m*/*z* 29) or more strongly chemisorbed CO on Fe sites. However, our results are consistent with an earlier study that Fe will segregate on the surface, which is responsible for the large H_2_ production of this sample in both the in situ experiments and under flow conditions, leaving the Cu underneath in a metallic state or the exposed Cu in inactive Cu(II).

### 2.4 Theoretical Modeling of the Reaction Mechanism

In situ spectroscopy data have revealed the presence of both cationic Zn species under CO_2_ reduction reaction as well as a Zn–Cu alloy. For the first scenario, calculations were performed using a model system consisting of a small ZnO cluster as the Zn(II) species placed on a Cu(111) slab. We investigate the Zn–Cu alloy as a second scenario, whereby a slab model was created from the X-ray crystal structure of the lowest energy polymorph of ZnCu.^71^

#### 2.4.1 Case of Cu/ZnO

Surface energy calculations show that Cu(111) is the most stable Cu facet and thus is the most expressed low-index Cu surface found in typical Cu nanoparticles as determined via the Wulff method.^72−74^ The analysis of the morphology of the particles in CuZn/G presented in Figure 1e is consistent with abundant (111) planes. Hence, it is reasonable to model the Cu/ZnO system by considering a small ZnO cluster supported on the Cu(111) facet.

This system has been investigated by Reichenbach et al.,^75^ with global minimum structures being obtained for a variety of small Zn*_x_*O*_y_* clusters supported on Cu(111). In the present work, we use one of these global minimum energy structures, Zn_7_O_9_@Cu(111), as an ideal starting point for our investigations; this system serves as a model to investigate the interaction between various adsorbates and intermediates and the different ZnO and ZnO–Cu interfacial sites present on the catalyst surface (Figure S16).

The calculated adsorption energies (Table 3) show that while typical physisorption energies for CO_2_ were obtained for both the ZnO and Cu sites (Figures S17 and S18), a marked difference was observed for chemisorption behavior. On the Cu interfacial site (Figure S19), a local minimum energy structure was obtained featuring a bent CO_2_ adsorbate but was determined to be metastable with respect to gas phase CO_2_. Previous work investigating unsupported Cu surfaces did not identify any such species for Cu(111), suggesting that the presence of the adjacent ZnO cluster plays a role in facilitating this process, albeit only to a very limited extent, since the adsorption energy for bent CO_2_ at the Cu interfacial site is still considerably more endothermic than that determined previously for CO_2_ on unsupported Cu(110) and Cu(100) surfaces.^76^ Optimization of a bent CO_2_ species placed above the ZnO cluster resulted in the emergence of a carbonate species, with the ZnO cluster undergoing significant rearrangement to accommodate the carbonate, corroborating the experimentally observed carbonate species on ZnO (Figure S20). Furthermore, it was found that the formation of this carbonate species is significantly exothermic, in line with extensive previous experimental work reporting CO_2_ activation by ZnO via carbonate formation.^77−79^ H_2_O adsorption is also moderately exothermic. The H_2_O adsorption energies are comparable for both the ZnO and Cu sites but slightly more so for the ZnO site (Figures S21 and S22).

**Table 3:** Calculated Adsorption Energies for a Variety of Species on Different Sites of the Model Zn_7_O_9_@Cu(111) Catalyst Surface

adsorption energy (eV)									
site	CH_4_	H_2_CO	CH_3_OH	H_2_	2H	H_2_O	CO_2_ (phys.)	CO_2_ (chem)	CO
Cu/ZnO interface	–0.241	–0.482	–0.744	–0.102	–0.414	–0.664	–0.353	+0.531	–1.076
ZnO	–0.195	–0.563	–0.792	–0.083	+0.700	–0.684	–0.253	–0.736 (carbonate)	–0.136

In contrast, for CO, while adsorption is exothermic both on the ZnO and Cu interfacial sites, adsorption is considerably more exothermic on the Cu interfacial site (Figures S23 and S24). Notably, the CO adsorption energy on the ZnO site is more exothermic than for any other species, suggesting that strongly bound CO may serve to poison the catalyst over time if it is not consumed by reactive processes (e.g., hydrogenation). It also explains the lower Faraday efficiency of CuZn/G toward CO, which once formed, strongly binds to the surface, where it is further converted.

The calculated adsorption energies for H_2_ (Figures S25 and S26), CH_4_ (Figures S27 and S28), and CH_3_OH (Figures S29 and S30) are consistent with physisorption, as can be seen from the calculated geometries in Table 3. The physisorption energy for CH_3_OH is comparatively more exothermic owing to the larger size of this adsorbate molecule, consequently resulting in greater Van der Waals interactions with the catalyst surface. CH_3_OH is more strongly adsorbed on both surface sites than CH_4_ but less strongly than the other reaction product CO, and this ensures that CH_4_ is released but also methanol. In each of these three cases, the physisorption energies are comparable for adsorption either on top of the ZnO cluster or on Cu sites at the Cu/ZnO interface.

For dissociated H_2_ (i.e., 2H), there is a clear difference between the Cu interfacial site and the ZnO site, with H_2_ dissociation over ZnO being moderately endothermic and conversely moderately exothermic at the Cu interfacial sites (Figures S31 and S32), which is consistent with the well-established role of Cu surfaces in facilitating H_2_ adsorption and dissociation,^80−83^ and suggests that while CO is strongly adsorbed at the interfacial Cu/ZnO site, atomic H formed on the adjacent Cu surface sites is available for further hydrogenation.

Adsorption of the intermediate H_2_CO is moderately exothermic on both the ZnO and Cu interfacial sites, slightly more so for the ZnO site (Figures S33 and S34). The optimized geometry for H_2_CO on the Cu interfacial site shows a nonplanar structure, in contrast to molecular formaldehyde, suggesting that H_2_CO binding on the Cu interfacial site is facilitated by filling of the C=O π* orbital by electrons originating from the substrate; this has been observed for formaldehyde adsorption on Cu(110) and Cu(100) surfaces.^76^ Unusually, the adsorption energy for formaldehyde adsorption on the ZnO cluster is slightly more exothermic, yet the planar geometry characteristic of molecular formaldehyde is retained. The adsorption interaction may be mediated by dipole–dipole interactions rather than by electronic processes; the dimensions of the H_2_CO molecule are commensurate with the dimensions of the ZnO cluster, allowing optimal dipole–dipole interactions between formaldehyde H and ZnO oxygen, and similarly between formaldehyde O and Zn atoms in the ZnO cluster, as can be seen from the calculated geometry.

Calculations were also performed to identify adsorption geometries for HCOO. Since HCOO is a bidentate intermediate, three distinct adsorption sites were investigated—the Zn–Zn site (Figure S35), the Cu–Zn site (Figure S36), and the Cu–Cu site (Figure S37). The Zn–Zn site was found to be significantly more stable than the other two, being more stable than the Cu–Cu site by 0.454 eV and the Cu–Zn site by 0.528 eV. As with the observed carbonate formation on the ZnO cluster from CO_2_, HCOO formation on the ZnO cluster resulted in a significant rearrangement of the structure of the ZnO cluster, with the bond formation between formate O and Zn being at the expense of bonding between Zn and O present in the ZnO cluster. In contrast, for the Cu–Zn and Cu–Cu adsorption sites, no significant changes in the structure of the ZnO cluster were induced. Additionally, OH adsorption was also considered, with OH being more strongly bound at the Cu site at the interface—adsorption at this site was determined to be more stable by 1.135 eV, a significant amount, suggesting that H_2_ formation must primarily occur over Cu rather than over ZnO (Figures S38 and S39).

#### 2.4.2 Case of the CuZn Alloy

The crystal structure and lattice parameters of the CuZn alloy with a 1:1 stoichometry^71^ were obtained using eqs 1 and 2 (see Section 4.3), whereby only the first half of each slab was allowed to relax for each termination. Table 4 shows the comparative surface energies and surface area of each termination, and the resulting energies were used to construct a Wulff nanoparticle for a truncated octahedron, in line with the Cu/Zn structure observed from the experimental SEM micrographs (Figure 1). Based on the Wulff construction shown in Figure S40 (see the Supporting Information), it was decided to focus on the adsorption energies of various partially oxidized hydrocarbon intermediates on the [001] and [110] surfaces only. Adsorption modes are depicted in Figures S41–S47 and Table S9 (Supporting Information). Table 5 shows the adsorption energies for each species as calculated by eq 3. The adsorption of formate was calculated with reference to the sum of one molecule of CO_2_ and half a molecule of H_2_ under vacuum.

**Table 4:** Relaxed Surface Energies (σ) and Surface Areas (a^2^) for Each Possible Termination of CuZn^a^

Zn_Top_^b^		Zn_Top_^c^		Cu_Top_^d^		Cu_TopTop_^e^		S^f^		S^g^		S^h^	
σ^b^	*a*^2 ^b	σ^c^	c*a*^2 ^c	σ^d^	σ^d^	σ^e^	*a*^2^ e	σ^f^	*a*^2 ^f	σ^g^	*a*^2 ^g	σ^h^	*a*^2 ^h
1.36	139	1.71	139	1.35	139	1.75	139	1.30	197	1.60	271	1.69	271

a All surface energies are given in J·m^–2^ with the corresponding areas shown in Å^2^.

b Pristine Zn terminated (001).

c reconstruction Zn terminated (001).

d Pristine Cu terminated (001).

e reconstruction Zn terminated (001).

f (011).

g Cu terminated (111).

h Cu terminated (111).

**Table 5:** Adsorption Energies for Important Species onto the Low-Index Surfaces of CuZn

Adsorption Energy/eV								
site	CH_4_	H_2_CO	HCOO	H_2_	*OH H*	H_2_O	CO_2_	CO
[001] Cu_top_^a^	–0.20	–0.65	–1.42	–0.26	–0.52	–0.37	–0.16	–1.10
[001] Cu_top_^b^	–0.32	–1.13	–1.42	–0.56	–0.49	–0.74	–0.34	–1.21
[001] Zn_top_^a^	–0.19	–0.70	–1.51	–0.75	0.08	–0.17	–0.67	–0.48
[001] Zn_top_^b^	–0.19	–1.53	–2.02	0.01	–0.18	–0.81	–0.86	–1.04
[110]	–0.18	–0.48	–1.08	–0.45	–0.57	–0.49	0.19	–1.00
[111] Cu_top_	–0.22	–0.62	–1.16	–0.10	–0.47	–0.58	0.20	–1.14
[111] Zn_top_	–0.18	–0.78	–0.74	–0.07	–0.26	–0.49	–0.06	–1.31

a [001] surfaces reconstructed to remove any perpendicular dipole.

b [001] unreconstructed surface.

In general, adsorption is highest on the unreconstructed [001] surfaces because chemical adsorption of most oxygen-containing species leads to major reconstruction of the uppermost layers and therefore these facets can be considered as metastable.

CH_4_ absorbs more exothermically on the Cu-terminated [001] surface than any other species under investigation. This means that on the most dominant surface of a potential nanoparticle, methane would likely not desorb, and indeed no facet would be predicted to desorb formate. From the calculated adsorption energies, we would expect that formaldehyde would be selectively desorbed by the (011) facet, while the reduction of CO_2_ would proceed through to formic acid on all other facets, except for the Cu-terminated (111) surface where some competitive desorption of CO is also possible. CO is very strongly adsorbed on both the (011) and (111) facets observed experimentally; however, these two surfaces exhibit very different activity toward water. Surface-mediated water dissociation is preferred on the (011) surface as opposed to the preference for molecular water adsorption seen on the (111) termination. The more exothermic binding of hydrogen atoms to the (011) surface could explain the importance of that surface in the further reduction of HCOO to HCOOH. Importantly, on both the experimentally observed facets, the driving force to formate is stronger than to either carbon monoxide or formaldehyde, which can explain the selectivity of formic acid as a solvent phase product. The formation energy of both surfaces, the methoxy radical (CH_3_O) and methanol, was calculated with reference to formaldehyde and hydrogen by the (111) facets. Formation energies of −2.45 and −1.88 eV were determined for the formation of the methoxy radical with the Cu- and Zn-terminated (111) surfaces, respectively, while the comparative formation energies for methanol were −2.12 and −2.14 eV. Therefore, the hydrogenation of formaldehyde is highly favored on both surfaces, with the Zn termination slightly promoting a second hydrogenation to methanol. Indeed, when taking into account the increase in entropy and desorption, it could be argued that both possible (111)-facets could promote the formation of CH_3_OH.

## 3 Conclusions

The combined soft and hard X-ray spectroscopic data have shown dynamics that are characteristic of a Cu-active state producing mainly methanol, methane, and carbon monoxide. Such an active state is facilitated by the addition of a second metal, with Zn enabling more stability of the active state if compared to Fe.

We focus on the CuZn/G system as it affords a clear picture of the electroactive state of Cu for CO_2_RR. If we consider the analysis of the Cu K-edge and Zn K-edge XANES and EXAFS under CO_2_RR conditions (−2 V vs Ag/AgCl), it is possible to conclude that Cu and Zn atoms are intimately dispersed, forming an alloy with no Zn metallic phase segregation.

Such a description of the electrode as predominantly metallic in bulk is complemented in this work by the surface-sensitive soft X-ray spectroscopic data at the Cu L-edges, O K-edge, and C K-edge, which reveal for the first time the Cu ligand species, which are relatable to the reduction products observed. In particular, we observe that under OCP, Cu(I)–O species such as those found in cuprous oxide are confirmed to be present at the interface and consumed only when the potential is lowered sufficiently enough to trigger CO_2_RR, indicating the important role of this site. These species are stabilized in electrocatalysts active for CO_2_RR.

We also show for the first time a Cu electrified interface populated by adsorbed species as indicated by the R3 resonance at 935.5–937 eV, described in the literature as −CO adsorbate and H-adsorbate, amongst others. DFT spectra simulations have allowed us to describe the nature of this Cu L-edge species as a Cu(I)–L species with the ligands carrying an electron–hole. The comparative analysis with the C K-edge allows us to postulate that such a species could be of the form M=C=O< = >M≡C–O^+^ (resonance at 286.7 eV), which would be susceptible to undergoing coupling reaction with adjacent adsorbates such as with H, OH, and e^–^, their relative abundance at the interface determining whether methanol or methane is produced. Indeed, previous work discussed the commonalities in the mechanistic pathways of these two reactions.^84^ We expect that the broad resonance also contains more reduced/hydrogenated chemisorbed species, consistent with the C K-edge spectra.

The explanation for this mechanism can be obtained from the computational study presented herein. Our results show that the alloy model would not lead to the formation of methane, and it is a better model to explain the formation of formate species. On the other hand, the ZnO/Cu model better matches the experimental findings, thus implying that the Cu/Zn(II) interfacial region is key for methanol or methane formation. Accordingly, we find that CO chemisorption is very strong at the Cu–Zn interfacial sites for both the Cu/Zn alloy and ZnO/Cu models, thus leading to further hydrogenation to methane rather than CO desorption, as observed on pure Cu. Such a model would also explain the in situ data showing that the CO is chemisorbed on a more electron-deficient Cu–Zn site compared to a Cu–Cu site, leading to the generation of an electron–hole in the ligand and thus the formation of a species highly reactive toward an electron/proton-coupled reaction, with H species more available at the neighboring Cu–Cu sites. Furthermore, the experimentally observed carbonate layer formation was mirrored by the DFT calculations performed for the ZnO/Cu model, while carbonate-like species were not observed in the calculations of the Cu/Zn alloy model, which implies that the Cu/Zn(II) phase, containing Cu(I) sites, is key to the formation of the carbonate layer, which can be interpreted as a means of initial CO_2_ activation prior to subsequent hydrogenation during the CO_2_RR reaction (the R3 resonance might be due to carbonate ions adsorbed on reduced Cu). Further experimental studies are required to confirm this.

The CuFe/G systems show similar dynamics, although the slightly different C and O speciation explains the different selectivity observed. The CuFe/G systems show differences at both the Cu L-edge (shift of the R3 resonance to higher energy) and the C K-edge (C–H resonance at lower energy) as well as the high formation of the *m*/*z* 29, which could be related to the higher ability of this element toward formic acid or C–C coupling. Nevertheless, the observation of the same dynamics confirms the general validity of the mechanism for the activation of CO_2_ and the importance of surface-sensitive, operando soft X-ray spectroscopy to unveil this aspect. More importantly, however, the tendency of Fe to segregate (thus its inability to form a stable Cu dispersed phase) makes this element unsuitable.

We thus clarify the role of the heteroatom as a promoter of Cu(I) species formation, which our results show is a key requirement for effective CO_2_ activation. We also show the importance of the topological characteristic of the electrode surface in which CO_2_ activation sites (e.g., Cu(I)) must coexist together with the exposed island of metallic Cu to provide the needed reserve of e^–^ and H^+^. This balance determines at any time the selectivity toward CO_2_ reduction products. In summary, we were able to identify new mechanistic insights into the formation of hydrogenated C products from CO_2_ over Cu-based electrodes, with dynamics involving ligated species on Cu(I) sites with electron–hole characteristics, which makes them susceptible to undergoing coupling reactions. DFT modeling has allowed us to describe the nature of the active site during these experiments as a dispersed, isolated, oxidized Zn species within highly dispersed Cu, where interfacial Cu–Zn species allow a stronger CO chemisorption, which is further hydrogenated by H species adsorbed on adjacent Cu–Cu sites. The relevance of this work stems from the fact that for the first time, we were also able to monitor and identify C and O species at the electrode/electrolyte interface, which correlate well with the product distribution observed and, for the first time, provided a comprehensive description of the reactive interface.

## 4 Experimental Methods

### 4.1 Preparation of the Electrodes

Copper was electrodeposited on the carbon paper (FUELCELL store) following the procedure previously reported.^47^ Accordingly, carbon paper cut into pieces of dimensions 1 cm × 2 cm was used. The electrodeposition was carried out using a three-electrode method and a H-shaped electrochemical cell. The potential of the carbon paper working electrode was controlled by a potentiostat (Gamry-Interface 1010E). A Pt wire (99.99%) and an Ag/AgCl (saturated KCl) were used as a counter and reference electrode (RE), respectively. During the experiment, only 1 cm × 1 cm area of the carbon paper (approximately 10 mg) was immersed in 5 mM CuSO_4_ aqueous electrolyte solution (prepared using anhydrous CuSO_4_ powder, 99.99%, Sigma-Aldrich), which was previously flashed with pure N_2_ gas. The deposition was carried under a potential of −0.7 V vs Ag/AgCl and in a N_2_ atmosphere for 300 s with a total charge exchanged corresponding to ca. 0.6% in weight of Cu. Subsequently, the electrode was transferred in a N_2_-saturated, 5 mM aqueous KCl solution (prepared using KCl powder, 99%, Sigma-Aldrich) and reduced under a potential of −0.7 V vs Ag/AgCl for 300 s. During the reduction, N_2_ was continuously bubbled into the electrolyte. Herein, this sample is referred to as Cu/G.

For the preparation of the bimetallic electrodes, Cu/G samples were placed in a crucible and impregnated dropwise with 1 mL of a 1 mM aqueous solution of either Fe or Zn sulfate (prepared from FeSO_4_ heptahydrate and ZnSO_4_ heptahydrate, respectively, Sigma-Aldrich), and the solvent was dried at 393 K on a hot plate. These electrodes are referred to as CuFe/G and CuZn/G, respectively.

The graphene deposition on the electrodes was performed following a modified version of the procedure described in ref (35). Chemical vapor deposition (CVD) growth commercial 10 mm × 10 mm monolayer graphene on a Cu substrate with poly(methyl methacrylate) (PMMA) was purchased from Graphenea. First, the as-received material was washed several times with acetone to remove the PMMA layer. Subsequently, it was floated on a (NH_4_)_2_S_2_O_8_ solution for Cu etching using a funnel equipped with a stopcock. Once the etching was completed, as observed by the visual disappearance of the Cu layer, the solution was gradually replaced with distilled water leaving the graphene layer floating on it. The graphene layer was then transferred onto the electrode via wet transfer.

### 4.2 Characterization Techniques

#### 4.2.1 In Situ Set up for Combined Electrochemical and Soft X-ray Spectroscopic Studies

**Figure 6:**
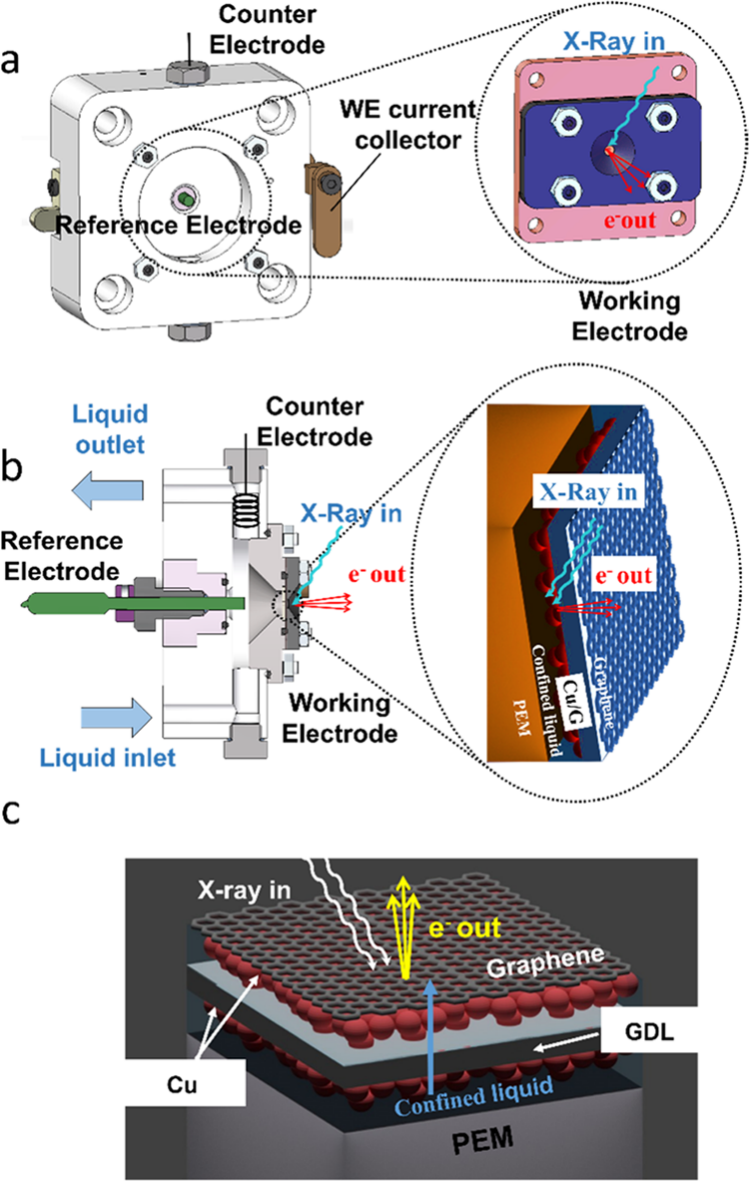
In situ electrochemical cell for soft X-ray spectroscopy: (a) front view of the two-plate lid hosting the working electrode (WE). (b) The side view of the three-electrode arrangement and the membrane electrode assembly (MEA). (c) The polymeric electrolyte membrane (PEM); the porous Cu/G electrode composed of a porous graphitic gas diffusion layer (GDL) on which Cu nanostructures are electrodeposited on both sides; confined liquid permeating through the pores of the GDL and trapped within this volume by the graphene top-layer. Note that volatile species in the electrolytes escape into the evacuated chamber through large holes in the graphene and are detected by online mass spectrometry.

A three-electrode electrochemical flow cell for in situ spectroscopy designed for the ambient pressure end station of the ISISS beamline at BESSY II/HZB was used (Figure 6a).^50^

The cell configuration consists of a Pt wire as the counter electrode (CE) and a Ag/AgCl (3M) as the reference electrode (RE) (DRIREF-2SH, World Precision Instruments), both immersed in the liquid electrolyte (CO_2_-saturated 0.05 M KHCO_3_) flowing continuously through the cell by means of a syringe pump. The CE and RE immersed into the electrolyte stream are separated from the evacuated XPS chamber of the end station by a sandwiched membrane electrode assembly (MEA) based on a reinforced-anionic polymeric electrolyte membrane (PEM) of type FAD-PET-75 membrane, FUMASEP FAD-PET-7 purchased from FUMATEK CH (FUMATECH BWT GmbH). Before use, the membrane was cut into small pieces to fit the in situ cell, thus was exchanged in 0.5 M NaCl at 298 K for 72 h and then left in 0.1 M KHCO_3_ solution until use. The WE is based on graphitic carbon paper, previously treated as described in the Section 4.1, placed between the PEM and the lid of the cell. No hot-pressing of the working electrode (WE) and the PEM was performed. The aqueous electrolyte diffuses through the PEM, ensuring ion conductivity. In order to keep the MEA under a realistic hydration state, a single layer of graphene was deposited on the WE, as described in the Section 4.1. This layer prevents the liquid electrolyte, diffused through the PEM and into the WE side, from being pumped down into the evacuated chamber. This cell configuration and WE with a graphene layer deposited on one side, as described in Figure 6b,6c, enable the wetting of the working electrode by a stagnant electrolyte solution in an enclosed environment. A potentiostat (SP-300, Bio-Logic Science Instruments SAS, France) is used to control the potential of the WE with respect to the RE and to measure the current between CE and WE. A quadrupole mass spectrometer (QMS) is used to monitor the gas composition online in the XPS chamber.

Using this cell, the samples are first subjected to several cyclic voltammetry (CV) cycles to facilitate the wetting of the electrode by the electrolyte (Figure S9a) and subsequently to the chronoamperometric-combined spectroscopic investigations at successively applied potentials. CVs of the Cu/G sample in Figure S9a show anodic peaks above −0.3 V vs Ag/AgCl, which are typical of Cu oxidation,^85^ confirming the well-functioning of the cell.

The spectroscopic signal as a total electron yield signal was collected through a Cu wire placed in close proximity to the WE using a current amplifier. To minimize beam damage, each spectrum was collected in a fresh position by moving the manipulator along one direction for the distance corresponding to the beam size. Measurement time for one spectrum is 1–2 min.

Additional technical information on the characterization techniques used in this study and electrocatalytic tests are reported in the Supporting Information.

### 4.3 Computational Methods

#### 4.3.1 Adsorption Modeling

Density functional theory (DFT) calculations were performed for two model systems to compare the adsorption and activity behavior of the Cu/Zn alloy and Cu/Zn oxide external overlayer phases observed experimentally, as detailed later in the corresponding section of the text, the latter being approximated by modeling the interfacial region between a small ZnO cluster adsorbed on a Cu(111) surface, hereafter referred to as ZnO@Cu(111). For the computational investigation of key adsorbed species over model Cu/Zn alloy and ZnO@Cu(111) systems, periodic plane-wave DFT as implemented in the VASP code^86−89^ was used to perform calculations to obtain optimized adsorption geometries for key reactants, intermediates, and products associated with the various mechanistic pathways for CO_2_ reduction, namely, CH_4_, CO_2_, CO, H_2_, H_2_O, H_2_CO, and CH_3_OH, as well as bound intermediates including HCOO and OH. Calculations were also performed to assess the relative surface energies of the various reconstructions of the CuZn alloy surface.

For the Cu/Zn alloy system, full optimization of the crystal structure coordinates of CuZn^71^ led to a reduction in lattice constant of >0.01 Å. Low-index facets were then created using the metadise (minimum energy techniques applied to dislocation interface and surface energies) code,^90^ and surface energies were determined via the following equations
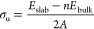
1
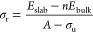
2

The energies for each unrelaxed surface are given by eq 1, whereby the energy of the slab (*E*_slab_) is subtracted from the bulk energy (*nE*_bulk_) over two times the surface area *A* (because two surfaces are created per facet). To obtain relaxed surface energies that can be used to construct a Wulff morphology,^73,91^ for a theoretical nanoparticle, eq 2 adds a term to denote the energy associated with relaxing one surface while freezing the bottom two layers to preserve the morphology of the bulk. For the ZnO@Cu(111) system, the model catalyst consists of a Zn_7_O_9_ cluster supported on a six-layer *c*(3 × 3) Cu(111) slab. This model is based on previous computational studies conducted by Reichenbach et al.,^75^ in which a genetic algorithm was employed to perform an unbiased Monte Carlo exploration of the phase space for Zn*_x_*O*_y_* clusters supported on Cu(111) surfaces in order to identify the most stable supported cluster structures. The *c*(3 × 3) Cu(111) slab support was chosen as it is sufficiently large to accommodate the ZnO cluster, ensuring that there is sufficient separation between ZnO clusters in adjacent periodic images, as well as providing surface regions that correspond to both ZnO/Cu interfacial regions and Cu-only regions. The model ZnO@Cu slabs were separated by an 18 Å vacuum gap, and a correction was applied to eliminate any spurious dipole moments across the slab. All ZnO and adsorbate atomic coordinates were relaxed during optimization, along with the top 4 layers of the Cu support, until atomic forces were converged to within 0.01 eV·Å^–1^.

All VASP DFT calculations were performed using the Perdew–Burke–Ernzerhof (PBE) functional,^75^ with a dispersion correction applied using the D2 scheme devised by Grimme,^92^ in order to account for the weak van der Waals interactions that are key to determining the physisorption behavior of relevant species such as CH_4_, CH_3_OH, and CO_2_. Inner electrons were replaced by projector-augmented waves (PAW),^93−95^ and the valence states were expanded in plane waves with a cutoff energy of 450 eV. The threshold for the electronic convergence of the self-consistency cycles (SCF) was set to 10^–5^ eV, with the convergence determined by the Blöchl smearing method.^94^ All adsorption geometry optimizations were performed with a force threshold of 0.01 eV·Å^–1^ for ionic relaxation. For the ZnO@Cu(111) system, sampling of the Brillouin zone was performed using a single Γ-centered *k*-point due to the large size of the slab supercell, while for the Cu/Zn alloy system, adsorption energies were determined with a 5 × 5 × 1 fine *k*-point mesh.

For both systems, the adsorption energies are calculated according to the following equation
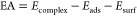
3whereby the adsorption energy (EA) is determined by subtracting neutral adsorbate(s) under vacuum (*E*_ads_) and the energy of the pristine surface (*E*_surf_) of each surface from the energies of the adsorbent molecule bound to each facet (*E*_complex_). The resulting values will determine the desorption enthalpies of the neutral species and allow an assessment of whether the selectivity is a desorption-driven phenomenon. While multiple binding sites were considered for each adsorption process, only the most energetically favorable ones are discussed here.

#### 4.3.2 Cu L_3,2_-Edge NEXAFS Simulation

For the simulation of Cu L_3_-edge, we used the plane-wave pseudopotential DFT method^94^ available within the CASTEP code, and a generalized-gradient approximation for the exchange–correlation energy was selected in the form of PBE functional^96^ and applied to supercells of the periodic cells of the selected materials. The dimensions of the supercells (3 × 3 × 3) of Cu_2_O and Cu(OH)_2_ were selected to prevent interactions between the periodically generated core holes. We introduced substitutional impurities of Zn or Fe in randomly selected Cu positions, relaxing the structures up to an energy change of 10^–6^ eV·atom^–1^. Self-consistent calculations were performed to a convergence value of 10^–7^ eV, and all ground-state simulations were converged with respect to the K-grid and cutoff energy for all explored materials. The simulations of the excited state have been performed with the same values of the K-grid and cutoff energy, following an evaluation that no noticeable changes were introduced upon selection of larger parameters. The simulations have been performed with a modest smearing factor (0.01 Ry) to help achieve convergence and account for the possible metallic nature of the sample surface explored with soft X-ray spectroscopy.

## Supporting Information Available

Experimental conditions and additional results data (PDF)
