# Correction to “Interfacial Chemistry in the Electrocatalytic Hydrogenation of CO_2_ over C-Supported Cu-Based Systems”

**DOI:** 10.1021/acscatal.4c04364

**Published:** 2024-08-09

**Authors:** Diego Gianolio, Michael D. Higham, Matthew G. Quesne, Matteo Aramini, Ruoyu Xu, Alex I. Large, Georg Held, Juan-Jesús Velasco-Vélez, Michael Haevecker, Axel Knop-Gericke, Chiara Genovese, Claudio Ampelli, Manfred Erwin Schuster, Siglinda Perathoner, Gabriele Centi, C. Richard A. Catlow, Rosa Arrigo

The corrected formula for eq 2 on page 5890 of the published Article is as follows:
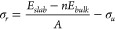
2

The description of the formula in the published article is correct.

All the numerical data reported in the published article are correct and used the correct form of the equation.

