# Supplementary material for: Interfacial Chemistry in the Electrocatalytic Hydrogenation of CO2 over C-Supported Cu-Based Systems
Source: ACS Catal. 2023 Apr 14;13(9):5876–95. doi: 10.1021/acscatal.3c01288 (PMC10167656; doi:10.1021/acscatal.3c01288)
Supplement: Supplementary file 1 — cs3c01288_si_001.pdf [file cs3c01288_si_001.pdf]

Supporting information

Interfacial chemistry in the electrocatalytic hydrogenation of CO_2 over C-supported Cu-based systems

Diego Gianolio,[1] Michael D. Higham,[2,3,4] Matthew G. Quesne,[2,3] Matteo Aramini,[1] Ruoyu Xu,[5] Alex I. Large,[1] Georg Held,[1] Juan-Jesús Velasco-Vélez,[6,7] Michael Haevecker,[6,7] Axel Knop-Gericke,[6,7] Chiara Genovese,[8] Claudio Ampelli,[8] Manfred Erwin Schuster,[9] Siglinda Perathoner,[8] Gabriele Centi,[8] C. Richard A. Catlow,[1,2,3,4] Rosa Arrigo[10,1].*

1 Diamond Light Source Ltd., Harwell Science & Innovation Campus, Didcot, Oxfordshire OX11 0DE, UK.

2 Cardiff Catalysis Institute, School of Chemistry, Cardiff University, Main Building, Park Place, Cardiff, CF10 3AT, Wales, UK.

3 UK Catalysis Hub, Research Complex at Harwell, Rutherford Appleton Laboratory, R92, Harwell, Oxfordshire OX11 0FA

4 Department of Chemistry, University College London, 20 Gordon Street, London WC1H 0AJ, UK

5 Department of Chemical Engineering, University College London, 20 Gordon Street, London WC1H 0AJ, UK

6 Max-Planck-Institut für Chemische Energiekonversion, Stiftstrasse 34-36, 45470 Mülheim an der Ruhr, Germany.

7 Department of Inorganic Chemistry, Fritz-Haber-Institut der Max-Planck Gesellschaft, Faradayweg 4-6, 14195 Berlin, Germany

8 University of Messina, Dept. ChiBioFarAm, ERIC aisbl and CASPE/INSTM, V. le F.Stagno D' Alcontres 31, 98166 Messina, Italy.

9 Johnson Matthey Technology Centre, Reading, RG4 9NH, UK.

10 University of Salford, School of Science, Engineering and Environment, Cockcroft building, Greater Manchester M5 4WT, UK

* Corresponding authors: r.arrigo@salford.ac.uk

KEYWORDS: Operando spectroscopy, CO_2RR; Cu, Zn, Fe, electrocatalysts, DFT, XAFS

This supplementary Information file includes:

1. Experimental part:
   1.1. Characterization methods
      1.1.1. XPS and NEXAFS measurements
      1.1.2. XAFS measurements and Electrochemical cell for operando study
      1.1.3. SEM
   1.2. Procedure for the electrochemical reduction of CO_2 in liquid phase

2. Results
   2.1. Ex-situ Characterization of as prepared electrocatalysts
      - Table S1: XPS elemental analysis
      - Figure S1: Raman Spectroscopy on Cu/G
      - Figure S2: Scanning electron micrographs
      - Figure S3: Cu L_3-edge and O K-edge NEXAFS: Comparative analysis.
      - Figure S4 XPS and NEXAFS characterization of the fresh samples

   2.2. Evaluation of the electrocatalytic performances in flow electrocatalytic cells

- Figure S5: (a) Faradaic efficiency and specific current density towards CO_2 reduction products

2.3. Hard X-ray operando study
   2.3.1. Cu K and Zn K edges XAFS of Cu/G and CuZn/G in 0.1 M $KHCO_3$ at open circuit potential
- Figure S6: Fluorescent yield Cu K edge XANES for Cu/G.
- Table S2: Results of Linear combination fits on XANES and EXAFS data for Cu/G and CuZn/G in 0.1 M $KHCO_3$

   2.3.2. K-edge operando spectroscopy study of CuZn/G
   - Table S3: Evolution of Cu species during CV shown by results of LCF fits on XANES and EXAFS signal for CuZn/G (Cu K-edge).
   - Table S4: Results of Linear combination fits on XANES and EXAFS data for CuZn/G at Cu and Zn K-edges at constant voltages

   2.3.3. K-edge operando spectroscopy study of Cu/G
   - Figure S7: Operando Cu K edge XANES for Cu/G under potentiostatic control at different potential applied sequentially as indicated.
   - Table S5: Results of Linear combination fits on XANES and EXAFS data for Cu/G at constant voltages
   - Figure S8 SEM after operando Study

2.4. Soft X-ray operando spectroscopy
   2.4.1. Electrocatalytic performance determined during operando studies
   - Table S6: Average currents exchanged during the chronoamperometry under steady state at each voltage applied
   - Figure S9: (a) Cyclic voltammograms recorded in the in situ cell, preceding the in situ spectroscopic measurements for the electrocatalysts; mass spectrum during operando study for: (b) Cu/G; CuZn/G (c) and (CO_2RR active state) CuFe/G-1 (d).
   - Figure S10: Mass spectrum recorded during the chronoamperometry study for sample CuFe/G under HER selective state (a), and relative Cu L3-edge spectrum (b).

   2.4.2. Cu L- edges and O and C K edges NEXAFS operando studies
   - Figure S11: Soft X-ray in situ NEXAFS data under a stagnant $KHCO_3$ electrolyte at different voltages as indicated: Cu L_3-edge a), O K-edge b) and C K-edge c) spectra for Cu/G.
   - Figure S12 SEM after operando Study

2.5. Cu L-edges NEXAFS simulation
   - Figure S13: Comparative plot of experimental and simulated Cu L_3 Absorption edges for reference materials a) Cu metal, b) Cu_2O and c) CuO. Simulated spectra have been energy-shifted to overlap with experimental ones
   - Figure S14: Simulated Cu L_3 XANES intensity at the 2p-3d transitions, showing the decrease in intensity of the feature due to the lowest unoccupied d- and s-type electronic states
   - Figure S15: (left panel) Simulated Cu L_3 XANES intensity while increasing the Mulliken charge on Cu as a consequence of presence of electron-hole on

neighbouring oxygen. (right panel) Corresponding Cu Density of states for the values of Mulliken charge of 0.35 eV (continuous line) and 0.79 eV (dashed line)

- Table S7: Simulated Mulliken population following the introduction of either Zn or Fe (upper and lower panel, respectively) in supercell of Cu_2O and relaxation of the geometry
- Table S8: Simulated Mulliken population following the introduction of either Zn or Fe (upper and lower panel, respectively) in supercell of $Cu(OH)_2$ and relaxation of the geometry of the unoccupied orbitals, and consequently the transition probability and spectral intensity

2.6. Computational study

2.6.1. ZnO/Cu model

- Figure S16- S39

2.6.2. CuZn alloy model

- Figure S40-47
- Table S9

1. EXPERIMENTAL PART

1.1. Characterization methods

1.1.1.XPS and NEXAFS measurements

X-ray photoelectron spectroscopy (XPS) and ambient pressure near edge X-ray absorption fine structure (NEXAFS) measurements in the soft X-ray regime were carried out at the ISISS beamline at Helmholtz-Zentrum Berlin (HZB). The freshly prepared samples were directly exposed to vacuum (10^{-7} mbar) in the XPS chamber. XPS measurements were performed applying a suitable excitation energy corresponding to a kinetic energy (KE) of the photo-emitted electrons of 450 eV for the core levels Cu_{2p}, Zn_{2p}, Fe_{2p}, $O_{1s,}$ C_{1s}, andS_{2p}. The energy pass Ep was normally set to 20 eV. The beam-line setting was exit slit (ES) 111μm and fix focus constant (cff) 2.25 (cff=cosα/cosβ).

When fitting of the core levels envelopes was performed, Casa XPS software was used after subtraction of a Shirley background. Quantification of the elemental composition was carried out according to homogeneous model distribution. For quantification the spectra have been normalized to the impinging photon flux.

NEXAFS spectra in total electron yield (TEY) were performed using a Faraday cup placed in close proximity to the sample in the APXPS chamber.

The beam-line setting was exit slit (ES) 60 μm and fix focus constant (cff) 2.25 (cff=cosα/cosβ). The

exit slit value chosen enables an optimal compromise between high photon intensity and good spectral resolution.

The Cu L-edges and O K-edge spectra were processed by subtracting a linear background fitting the pre-edge absorption (between 527 and 530 for Cu L edges and between 525 and 529 eV) and normalizing by the edge height (mean value between 540 and 570 eV for the O K and between 970-980 eV for the Cu L-edges). The C K-edge spectra were processed by subtracting a linear background fitting the pre-edge absorption region (between 283 and 284 eV) and normalizing by the C K-edge height at the peak at 285.7 eV on the assumption that this component is the C=C bond resonance of the graphitic peak of the support, which remains unchanged during the measurements. The energy scale of for each spectrum was calibrated using features of the refocusing mirror drain current recorded simultaneously to the spectrum. No further calibration was performed using references spectra. The Cu L-edges NEXAFS spectra were fitted with a linear combination analysis of reference spectra for CuO, Cu_2O and Cu using Igor Software.

1.1.2.XAFS measurements and Electrochemical cell for operando study

X-ray absorption experiments (EXAFS and XANES) were performed at the B18 Core EXAFS beamline of Diamond Light Source. The measurements were carried out using the Pt-coated branch of collimating and focusing mirrors, and a Si(111) double-crystal monochromator. A couple of Pt-coated harmonic rejection mirrors were inserted before the first ion chamber and used to filter out photons with higher energy. The size of the beam at the sample position was ca. 1 mm (h) × 1 mm (v). The data were collected in fluorescence mode, by means of a 36-element solid state germanium detector (K_{max}=14), the ion chamber before the sample has been used for measurement of incoming photons (I0 filled with a mixture of 30 mbar of Ar and 1080 mbar of He to optimize sensitivity at 20% efficiency).

Samples were measured both in static and operando conditions. The design of the operando XAFS cell used is this work was reported earlier.[1] The cell is filled with 0.1 M $KHCO_3$ electrolyte and CO_2 is continuously supplied through an inlet placed though a leak-tight orifice in the lid of the cell and immersed into the liquid electrolyte.

Then, Cu K-edge spectra were measured first while running CV scans and then at fixed constant potentials successively applied in the order indicated for each cases.

A principal component analysis (PCA) done on a series of spectra collected at different potentials for both samples suggests that there are at least 3 components involved during the reaction for each catalyst, the target transform assigns these components to Cu metal, Cu_2O and $CuCO_3$. Note that Cu, Cu_2O, CuO, $Cu(OH)_2$, $CuSO_4$, CuS, $CuCO_3$ reference spectra for Cu and Zn, ZnO, $Zn(OH)_2$, $ZnCO_3$, $ZnSO_4$, $Zn(CH_3CO_2)_2$ reference spectra for Zn were used to fit the XANES spectra. $CuCO_3$ provided the best fit for the Cu(II) phase.

Data were normalized using the Athena[2] program with a linear pre-edge and polynomial post-edge background subtracted from the raw data. All XANES data were fitted with linear combination analysis using relevant spectra as reference. A linear combination fit (LCF) was performed on both Cu K XANES and EXAFS regions of the spectra measured during the voltage sweep experiments as well as the experiments at constant voltages. Fits were performed with Athena in the -20 to +80 eV range using relevant recorded spectra as reference, to describe variation in sample composition. Linear combination analysis on EXAFS data was performed in the range (2.5 to 10 $Å^{-1}$).

EXAFS fits were performed using ARTEMIS software.[2] Moreover, the interatomic distances and Debye-Waller factors were optimized by fitting the experimental data.

1.1.3. SEM

Scanning electron microscopy (SEM) and energy dispersive X-ray (EDX) analyses were performed on a Zeiss Ultra SEM operating at acceleration voltages of 1.6 and 15 kV.

1.2. Procedure for the electrochemical reduction of CO_2 in liquid phase

A compact-design electrochemical cell was used for the electrochemical reduction of CO_2 in liquid phase.[3] The cell has a three-electrode configuration: the working electrode (about 0.64 cm^2) was located at the cathode side, at a small distance from a saturated Ag/AgCl reference electrode to reduce the solution resistance. The electric contact with the working electrode was assured by a Pt wire. A commercial Pt rod (Amel) immersed in the anode compartment was used as the counter-electrode. The anode and cathode compartments were physically separated using a proton-conducting membrane (Nafion® 117, supplied by Ion Power). A 0.1 M $KHCO_3$ aqueous solution was used as the electrolyte both in cathode and anode compartments. To assure a uniform distribution of CO_2 in the cathode compartment, the electrolyte solution was introduced into an external reservoir and saturated with a continuous flow of pure CO_2 (10 mL min^{-1}). A peristaltic pump was used to continuously circulate the CO_2-saturated electrolyte solution through the cathode compartment and the external reservoir. A potentiostat/galvanostat (Amel mod. 2049A) was employed to supply a constant bias between the electrodes. The experiments were carried out at different voltages (-0.5/-2V vs Ag/AgCl range), checking stability of the behaviour for at least 30 min at each voltage applied. Sampling from the external container was carried out and used for the determination of the liquid products by Gas Chromatography-Mass Spectrometer (GC-MS, Thermo Trace 8000A EVO, Triple Quadrupole MS, column Stabilwax) and Ion Chromatography (IC Metrohm 940 with conductivity and amperometry professional detector Vario). The gas products were analysed at the outlet gaseous stream by Gas-chromatography (GC-TCD, Agilent 7890A, column 5A Plot). Before starting chronoamperometric experiments, cyclic voltammetry (CV) measurements were conducted on the electrocatalysts in the potential interval 0/- 2V (vs. Ag/AgCl) at a scan rate of 10 mVs^{-1}.

Sample were pre-treated before the experiments to eliminate impurities in the electrode and tests to check that the products detected derive from the electroreduction of CO_2 were made, as earlier described.[3] These preliminary experiments include tests with labelled CO_2. The experiments were carried out at different voltages (-0.5/-2V vs Ag/AgCl range). We find that the optimal behaviour in terms of higher production rates of products from CO_2 conversion was at the applied potential of -1.38 V vs RHE that was then used in the following tests. As earlier commented, we also checked the use of alternative analytical methods such as by NMR (nuclear magnetic resonance) finding preferable the analytical method described above.

2. RESULTS

2.1. Ex-situ Characterization of as prepared electrocatalysts

Table S1: XPS atomic composition of the fresh samples. [a]

Sample	C	Cu	S	O	M
Cu/G	54.1	25.8	0.5	19.6	
CuZn/G	48.5	4.9	3.3	26.8	16.4
CuFe/G	74.1	1.5	1	21.8	1.6

3. [a] Elemental composition in at% determined by applying an homogeneous distribution of the elements model. (M= Zn(II) or Fe(III)).

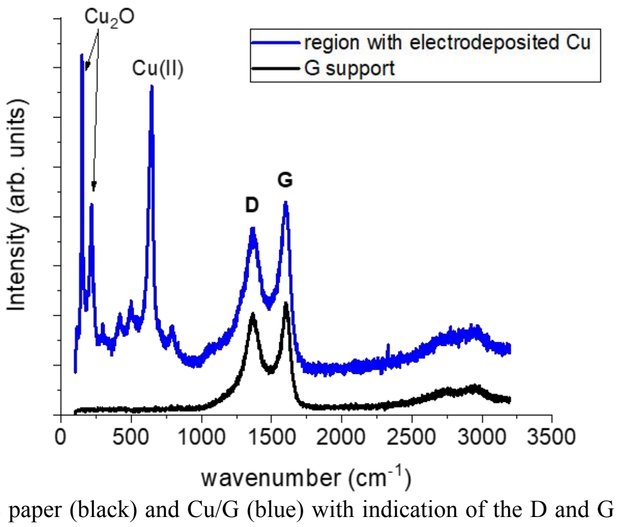

Figure S1: Raman spectra of C paper (black) and Cu/G (blue) with indication of the D and G bands of graphite as well as bands assigned to Cu oxides.

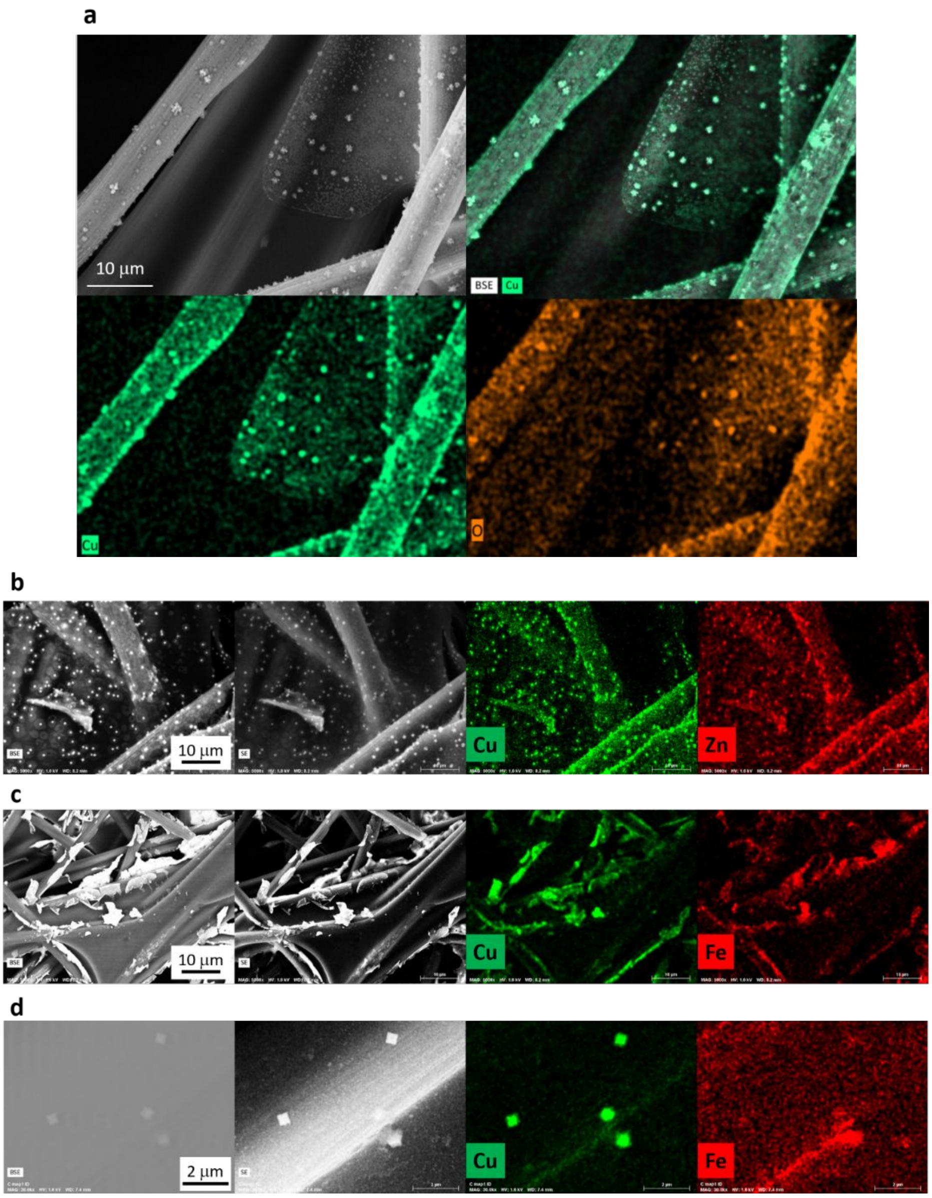

Figure S2: SE-SEM micrograph measured at 15 kV and corresponding EDX elemental mapping of Cu/G (a); CuZn/G (b) and CuFe/G (c and d).

XPS spectra in Fig. S3a and b indicate that Cl is the main impurity in Cu/G, whereas S is present more abundantly in CuZn/G and to a lesser extent in CuFe/G. Fig. S3c compare the surface sensitive Cu2p XPS spectrum of Cu/G to the one of CuZn/G. A marked difference is visible in terms of Cu abundance but also chemical nature. Particularly a component at 934 eV shows on CuZn/G indicates Cu(II) species as Cu sulphates,[4] consistent with S 2p data (Figure S3b). The quantitative surface elemental composition determined by XPS is reported in Table S1 of the supporting information. Accordingly, the surface of Cu/G is composed of approximately 25.8% of Cu atoms on C. The addition of another metal by wet impregnation and the formation of larger particles due to this treatment produces a pronounced decrease of the Cu content within the volume probed by this technique.

The analysis of the Zn L-edges and Fe L-edges in Figure S3d-e and, provides further insights into the

chemical speciation of the elements in these samples. We show that Zn in CuZn/G is present as Zn(II) species (sharp pre-edge feature at 1024 eV in the Zn L_3-edge NEXAFS spectrum). Interestingly, the O K-edge NEXAFS spectrum of CuZn/G in Figure S4b shows several resonances denoted in Figure S4b as A, B and C. Whilst the resonance C is typical for Zn oxides, the resonances at 530 eV (resonance A) and at 532 eV (resonance B) are assigned to Cu(II) species and Cu(I) species, respectively[5, 6] and were reported for Cu-doping of ZnO thin films.[7] Consistently, the pre edge feature of the Cu(II) shifts (Fig. S3d) to lower energy indicating an intimate chemical interaction between the Cu(II) and the Zn(II) cations in the starting electrocatalyst. It is possible to infer that despite the Zn being impregnated on the surface of the Cu oxide nanostructures, the Cu-Zn chemical affinity allows for the Cu to be dispersed in the external Zn(II)-overlayer during the thermal annealing step. A closer inspection of the Zn L edges spectrum for CuZn/G enables to identify a high edge jump at 1037 eV, which was also reported for ZnS, consistent with S 2p spectrum (Fig. S3b).[8] It can be therefore assumed that the external overlayer is as a mixture of a Cu-Zn oxide, sulphate and sulphide phases.

In CuFe/G, Fe is present predominantly as a mixture of Fe(II) and Fe(III) species (Figure S3e). Here we observe an almost quantitative reduction of Cu(II) sites (Fig. S4a) suggesting for a predominant Fe-containing overlayer on the cuprous oxide nanoparticles in the form of an oxide-sulphate mixed phase. As shown in the SEM images in Figure S2 c and d, this sample contains a portion of particles detached from the carbon support, which will not be available for electrocatalysis, due to the low contact with the conductive substrate. The electronic effect on the Cu exerted by the heteroatom are presented in in Figure S4a. Interestingly, the Cu(II) and Cu(I) transitions are found at a slightly different excitation energy for the three samples. This can be better evaluated by considering the energy difference between the two transitions, which is circa 2.46 eV for Cu/G and 2.75 eV for CuZn/G. This is due to the different bonding environment of Cu in the three samples.

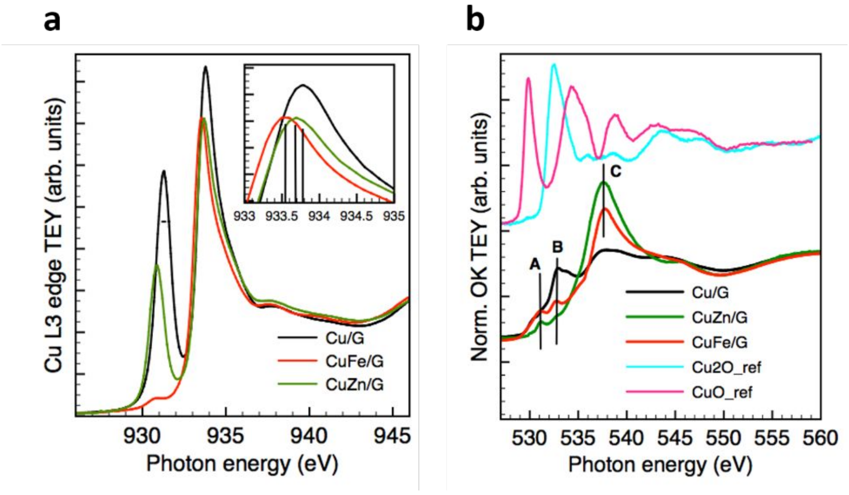

Figure S3: Comparison of the Cu L_3-edge TEY-NEXAFS spectra of the fresh samples in UHV: (a) Cu L-edges and (b) O K-edge.

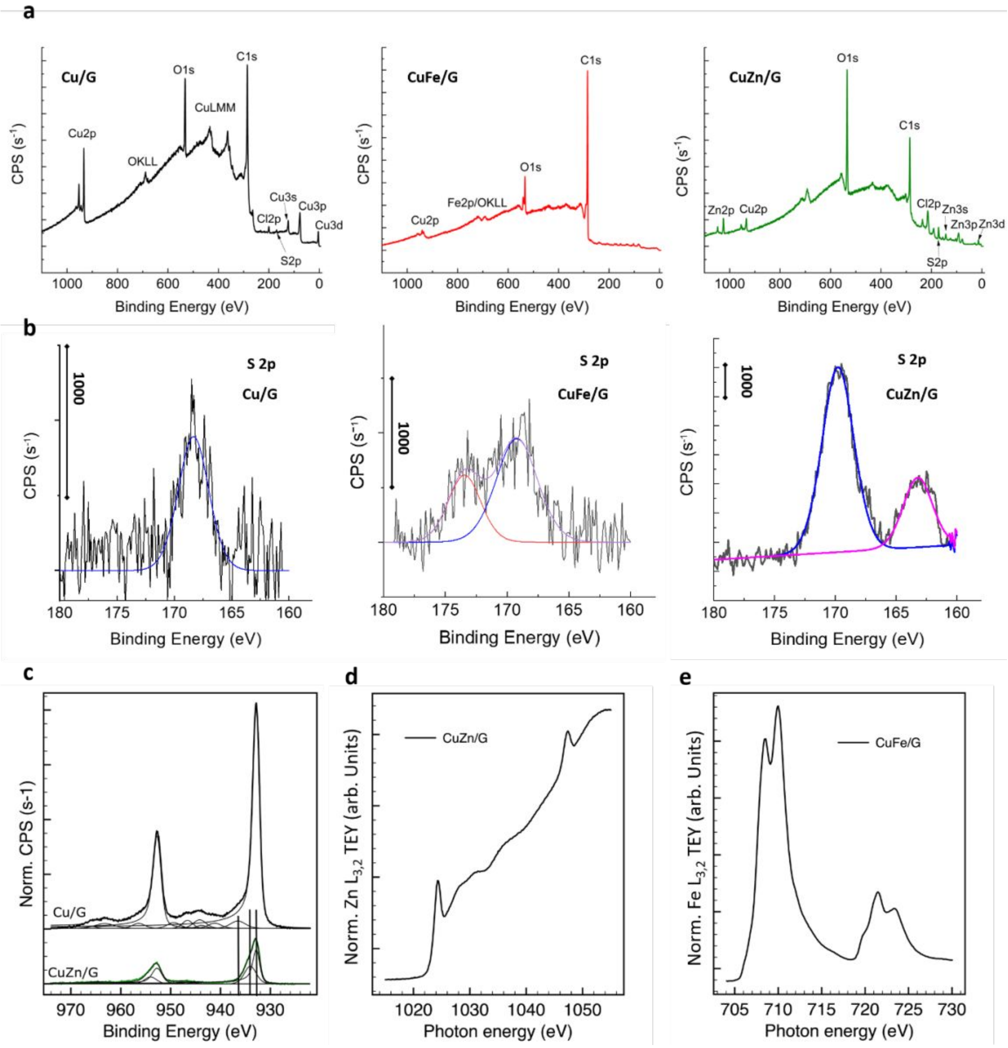

Figure S4: (a) XPS Survey of fresh samples as indicated; (b) S 2p XPS spectrum measured at hυ= 610 eV for fresh samples as indicated; (c) Fitted Cu 2p XPS spectrum measured at hυ= 1385 eV using fitting model reported in ref. 4; (d) TEY Zn L-edges NEXAFS spectrum of CuZn/G; (e) TEY Fe L-edges NEXAFS spectrum of CuFe/G.

3.1. Evaluation of the electrocatalytic performances in flow electrocatalytic cells

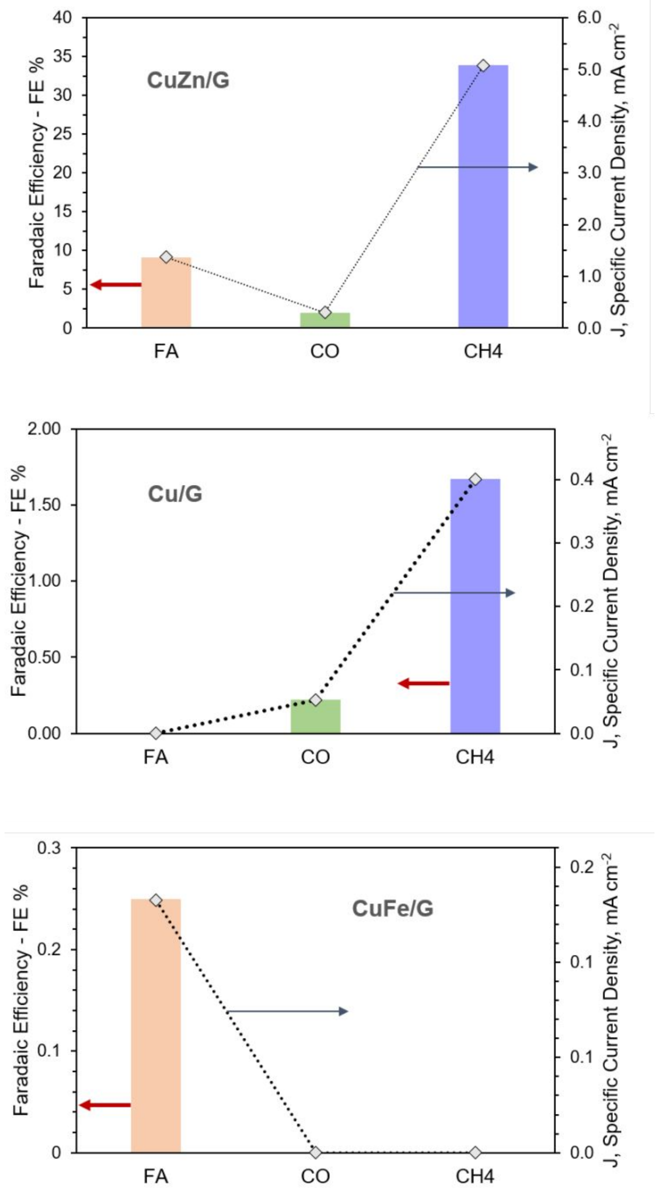

Figure S5: Faraday efficiency and specific current density towards main CO_2RR products.

2.3 Hard X-ray operando spectroscopy during CO_2 reduction

2.3.1 Cu K and Zn K edges XAFS of Cu/G and CuZn/G in 0.1 M $KHCO_3$ at open circuit potential

We compare the bulk electronic structure of the Cu/G and CuZn/G samples investigated by Cu K-edge X-ray absorption spectroscopy (XAS) under operando conditions in a CO_2-saturated 0.1 M solution of $KHCO_3$ (Fig. S6). Cu(0) and Cu(I) have no hole in the 3d states, whereas Cu(II) is a d^9 and therefore a weak quadrupole-allowed pre-edge peak is observed (peak A in Fig. S6a).[9]

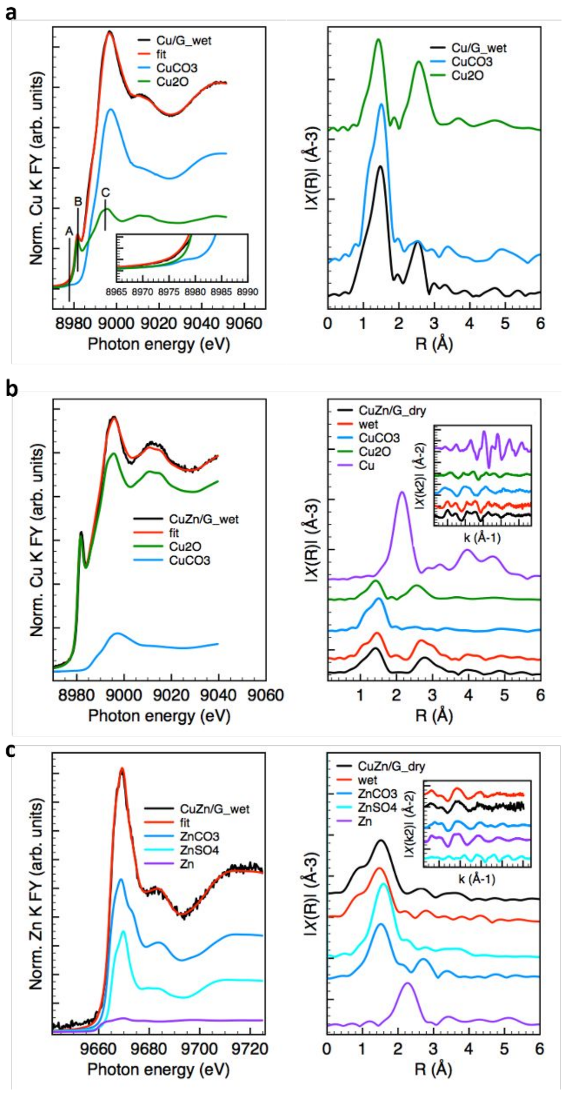

Figure S6*:* Fluorescent yield Cu K edge XANES for Cu/G (a) and corresponding k^2-weighted FT EXAFS. Fluorescent yield XANES for CuZn/G at the Cu K edge (b) and Zn K edge (c) and corresponding k^2-weighted Fourier transform EXAFS. XANES spectra of Cu and Zn standards are included weighted by the percentage found by LCF. Note that the measurements were taken upon immersion of the electrocatalysts in the liquid electrolyte solution and before any electrocatalytic tests were performed.

The Cu(I) is characterized by a dipole allowed 1s → 4p transition, which due to ligand field splits into two resonances B and C.[9] The EXAFS spectra and the linear combination fits (LCF) of the XANES for these samples using three components obtained from reference spectra (Cu metal, Cu_2O and Cu(II) species in $CuCO_3$) show consistently that the samples differ largely in terms of phase composition. $CuCO_3$ (blue component) is the prevalent phase for Cu/G (67% ca.), with the remaining fraction being Cu_2O (green component); in contrast CuZn/G is composed mainly of a Cu_2O phase (ca. 90%) and in minimal part of $CuCO_3$ (Table S2). No metallic component was identified in the bulk of the sample under equilibrium condition with the $KHCO_3$ solution. We compare the bulk electronic structure of the Cu/G and CuZn/G samples investigated by Cu K-edge X-ray absorption spectroscopy (XAS) under operando conditions in a CO_2-saturated 0.1 M solution of $KHCO_3$ (Fig. S6).

Cu(0) and Cu(I) have no hole in the 3d states, whereas Cu(II) is a d^9 and therefore a weak quadrupole-allowed pre-edge peak is observed (peak A in Fig. S6a).[9] The Cu(I) is characterized by a dipole allowed 1s → 4p transition, which due to ligand field splits into two resonances B and C.[9] The EXAFS spectra and the linear combination fits (LCF) of the XANES for these samples using three components obtained from reference spectra (Cu metal, Cu_2O and Cu(II) species in $CuCO_3$) show consistently that the samples differ largely in terms of phase composition. $CuCO_3$ (blue component) is the prevalent phase for Cu/G (67% ca.), with the remaining fraction being Cu_2O (green component); in contrast CuZn/G is composed mainly of a Cu_2O phase (ca. 90%) and in minimal part of $CuCO_3$ (Table S2). No metallic component was identified in the bulk of the sample under equilibrium condition with the $KHCO_3$ solution.

Table S2: Results of Linear combination fits on XANES and EXAFS data for Cu/G and CuZn/G in 0.1 M $KHCO_3$.

	XANES (-20/+80eV)				EXAFS (2.5-10 Å^{-1})			
Sample	Cu metal	Cu_2O	$CuCO_3$	Reduced c^2	Cu metal	Cu_2O	$CuCO_3$	Reduced c^2
Cu/G	0 (2)	34.9 (9)	65.1 (4)	0.00013	0 (4)	30 (4)	70 (2)	0.01198
CuZn/G (Cu K-edge)	0 (2)	86.7 (6)	13.3 (6)	0.00028	0 (4)	97 (3)	3 (3)	0.01876
	Zn metal	Zn carbonate	Zn sulfate	Reduced c^2	Zn metal	Zn carbonate	Zn sulfate	Reduced c^2
CuZn/G (Zn K-edge)	0 (3)	67 (2)	33 (3)	0.00083	0 (12)	75 (9)	28 (7)	0.11797

Note: Cu metal, Cu_2O and $CuCO_3$ were used as Cu standards, Zn metal, Zn carbonate and Zn sulphate were used as Zn standards. Reduced c^2 is an indicator of goodness of the fit independent by the number of components used (changes are strongly depending by experimental signal to noise). Errors reported in bracket.

The LCF is also used for the analysis of the Zn K-edge XAS. Accordingly, the spectrum resembles a mixture of ca. 33% of Zn sulphate distinguishable by an intense white line at 9670 eV and ca. 67% of Zn carbonate characterized by a pre-edge shoulder at 9666 eV and post edge feature at 9674 eV. The former one is a residue of the Zn precursor used, whereas the latter one is formed upon interaction with the bicarbonate solution. We can therefore conclude that in a $KHCO_3$ solution, under open circuit conditions, the electrodeposited Cu phase undergoes a surface carbonatation reaction, whose extent depends on the exposed surface area. In the case of CuZn/G, we assume that the larger cubo-octahedrons allows a lower Cu exposure and as a consequence the Cu_2O phase remains mostly unaltered. Moreover, we observe that the Zn(II) sulphates species are converted to carbonates. The EXAFS analysis provides additional information on changes in bond lengths upon immobilization of Zn(II) species. The first shell in $\chi(R)$ Fourier transformed data (Figure S6b), shows an evident shift of the peak position from 1.43 to 1.51 Å (phase uncorrected), corresponding to Cu-O bond lengths in Cu(I) and Cu(II) species. The second shell, related to metal-metal distances, has a lower intensity but shows an interesting splitting

of the peak in the Cu-K edge spectra upon Zn addition: The Cu-Cu contribution in Cu oxide is accompanied by a shoulder at higher distance that could be assigned to the Cu-Zn interaction. This is confirmed by the FT of the Zn K-edge spectrum for the same sample (Figure S6c) where a similar feature is present at the same interatomic distance, and can be assigned to the Zn-Cu contribution. The observation of an interaction between Cu and Zn atoms confirms the homogeneous distribution of both metals and excludes that the two elements are segregated in isolated phases. The electronic effect of Zn on Cu in the fresh electrocatalyst was also observed in the Cu L edges NEXAFS (Fig. S4a) and OK NEXAFS spectra Fig. S4b. A linear combination fit of the EXAFS k^2-weighted $\chi(k)$ data (Table S2) confirms the results obtained by LCF of XANES spectra with similar percentages of the different fractions for the two samples analysed.

2.3.2 K-edge operando spectroscopy study of CuZn/G

Table S3: Evolution of Cu species during CV[a] shown by results of LCF fits on XANES and EXAFS signal for CuZn/G (Cu K-edge).

	XANES				EXAFS		
CuZn/G (Cu K-edge)	Cu	Cu_2O	$CuCO_3$		Cu	Cu_2O	$CuCO_3$
OCP1	0	86,7	13,3		0	95,0	5,0
OCP to -0.81V	0	84,7	15,3		0	95,3	4,7
-1V	48,6	42,8	8,6		29,1	66,7	4,2
-1,2V	77,9	18,7	3,4		79,5	14,0	6,5
-1,4V	81,3	15,7	3,0		80,0	11,2	8,8
-1,56V	84,5	13,0	2,5		81,6	8,9	9,4
-1,65V	90,0	8,3	1,7		86,6	5,7	7,7
-1,43V	83,0	14,8	2,2		85,3	6,1	8,6
-1,28 to OCP	85,9	12,1	2,0		86,2	7,9	5,9
OCP2	74,4	17,0	8,6		77,1	10,5	12,3
+0,07V	58,9	29,4	11,6		63,7	23,6	12,8
+0,25V	59,1	26,7	14,2		60,0	28,5	11,4
+ 0,43V	60,7	20,7	18,6		57,2	25,5	17,4
+0,6V	55,3	22,3	22,3		53,9	18,7	27,5
+0,4V	55,9	19,9	24,1		56,4	22,5	21,1
+0,23 +0,04V	54,4	21,9	23,7		52,7	25,1	22,2
-0,15V	56,1	27,0	16,9		54,3	30,3	15,4
-0,37V	54,2	33,2	12,6		58,7	29,3	12,0

-0,50V	58,3	30,5	11,2		60,6	22,8	16,6
-0,68V	59,7	29,4	10,9		64,0	26,3	9,7
-0,85V	67,0	23,5	9,6		73,3	15,5	11,2
-1,00V	77,3	15,5	7,2		82,6	7,5	9,9
-1,2 to -1,65V	80,6	13,8	5,6		85,6	3,3	11,1

[a] the spectra were recorded continuously while changing the potential and similar spectra were merged together and herein reported as a voltage range.

Table S 4: Results of Linear combination fits on XANES and EXAFS data for CuZn/G at Cu and Zn K-edges at constant voltages. Results are in percentage.

	XANES (-20/+80eV)				EXAFS (2.5-10 Å^{-1})			
CuZn/G (Cu K-edge)	Cu metal	Cu_2O	$CuCO_3$	Reduced c^2	Cu metal	Cu_2O	$CuCO_3$	Reduced c^2
OCP1	0 (2)	86.7 (6)	13.3 (6)	0.00028	0 (4)	97 (3)	3 (3)	0.01876
+0,5V	67.8 (7)	13 (1)	19.2(3)	0.00007	66 (1)	11 (3)	23 (3)	0.02253
-0,5V	84.4 (7)	11 (1)	4.6 (3)	0.00007	83 (1)	11 (3)	6 (3)	0.01833
-1,0V	89.0 (7)	10 (1)	1.0 (3)	0.00007	87 (1)	11 (3)	2 (3)	0.02000
-2,0V	88.5 (9)	11 (1)	0.5 (4)	0.00014	92 (1)	6 (4)	2 (4)	0.03653

	XANES (-20/+80eV)				EXAFS (2.5-10 Å^{-1})			
CuZn/G (Zn K-edge)	Zn metal	Zn carbonate	Zn sulfate	Reduced c^2	Zn metal	Zn carbonate	Zn sulfate	Reduced c^2
OCP1	0 (3)	67 (2)	33 (3)	0.00083	0 (12)	75 (9)	28 (7)	0.11797
+0,5	14 (5)	64 (4)	22 (3)	0.00159	6 (15)	76 (12)	18 (9)	0.24433
-0,5	12 (5)	72 (4)	16 (3)	0.00203	14 (17)	65 (14)	21 (11)	0.32765
-1,0	28 (5)	67 (4)	5 (3)	0.00167	24 (6)	76 (6)	0	0.27398
-2,0	86 (5)	14 (4)	0 (3)	0.00166	70 (6)	30 (6)	0	0.27212

Note: Cu metal, Cu_2O and $CuCO_3$ were used as Cu standards, Zn metal, Zn carbonate and Zn sulphate were used as Zn standards. Reduced c^2 is an indicator of goodness of the fit independent by the number of components used (changes are strongly depending by experimental signal to noise). Errors reported in bracket. OCP1 is measured before the CV cycles in table S3.

A linear combination fit (LCF) was performed on both Cu K XANES and EXAFS regions of the spectra measured during the voltage sweep experiments as well as the experiments at constant voltages. The results of the LCF analysis during the preceding CV are reported in Table S3. We observe redox

dynamic involving the Cu with the highest abundance of Cu(I) formed at -0.3 V vs Ag/AgCl whereas the maximum reduction of Cu(I) → Cu0 occurs at potential below -0.84 V vs Ag/AgCl.

The fitted Cu K and Zn K edges XANES and EXAFS spectra measured at constant voltages are reported in Figure 4 of the main text. The quantitative analysis is summarized in Table S4. Consistent trends are observed in both the voltage sweep experiments and the constant potentials. We note that the composition of the electrocatalyst at low voltages depends on the preceding voltage applied and the time so the proportion of Cu(I) and Cu(0) changes in different runs at the same potential (Table S3). This is a direct manifestation of the changes in particles morphology or/and size within the electrodes with time.

2.3.3 K-edge operando spectroscopy study of Cu/G

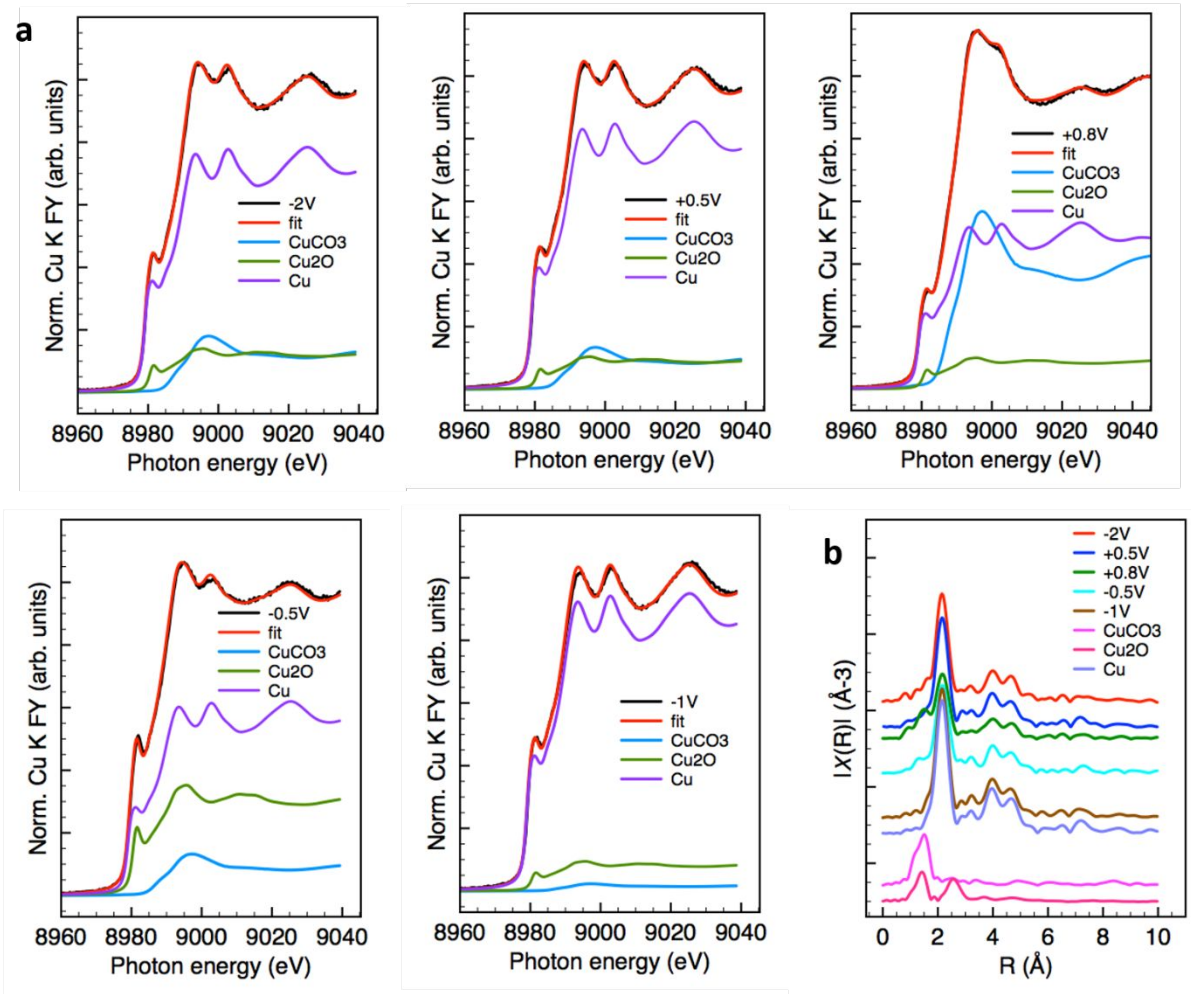

Figure S7: a) Operando Cu K edge XANES for Cu/G under potentiostatic control at different potential applied sequentially as indicated (-2 V, +0.5 V, + 0.8 V, -0.5 V -1 V); b) corresponding k^2-weighted FT EXAFS.

The quantitative analysis of the spectra in Figure S7 is reported in Table S5. The main phase in Cu/G -2V vs AG/AgCl is a metallic Cu phase, similar to CuZn/G; however, part of the electrocatalyst is still

present as Cu_2O and Cu carbonate. The application of an anodic potential (+ 0.8 V vs Ag/AgCl) induces the oxidation of the metallic Cu to carbonate which is then reduced to Cu_2O at - 0.5 V vs Ag/AgCl, whereas Cu^0 is the dominant phase at -1 V vs Ag/AgCl.

Table S5: Results of Linear combination fits on XANES and EXAFS data for Cu/G at constant voltages.

	XANES (-20/+80eV)				EXAFS (2.5-10 $Å^{-1}$)			
Cu/G	Cu metal	Cu_2O	$CuCO_3$	Reduced c^2	Cu metal	Cu_2O	$CuCO_3$	Reduced c^2
OCP1	0 (2)	34.9 (9)	65.1 (4)	0.00013	0 (4)	30 (4)	70 (2)	0.01198
-2,0V	73.2 (6)	13.6 (6)	13.1 (9)	0.00006	77(1)	8 (3)	15 (1)	0.01817
+0,5V	79.2 (6)	11.0 (6)	9.8 (8)	0.00006	79 (3)	12 (2)	9 (2)	0.01171
+0,8V	47.6 (7)	11.1 (7)	41.2 (9)	0.00007	49 (3)	3 (2)	48 (2)	0.01163
-0,5V	57.8 (7)	32.5 (7)	9.7 (9)	0.00007	64 (3)	33 (2)	3 (2)	0.01174
-1,0V	88.2 (7)	10.2 (7)	1.6 (9)	0.00007	92 (1)	0 (4)	8 (3)	0.01535

Note: Cu metal, Cu_2O and $CuCO_3$ were used as Cu standards, Zn metal, Zn carbonate and Zn sulphate were used as Zn standards. Reduced c^2 is an indicator of goodness of the fit independent by the number of components used (changes are strongly depending by experimental signal to noise). Errors reported in bracket. OCP1 is measured before the CV cycles.

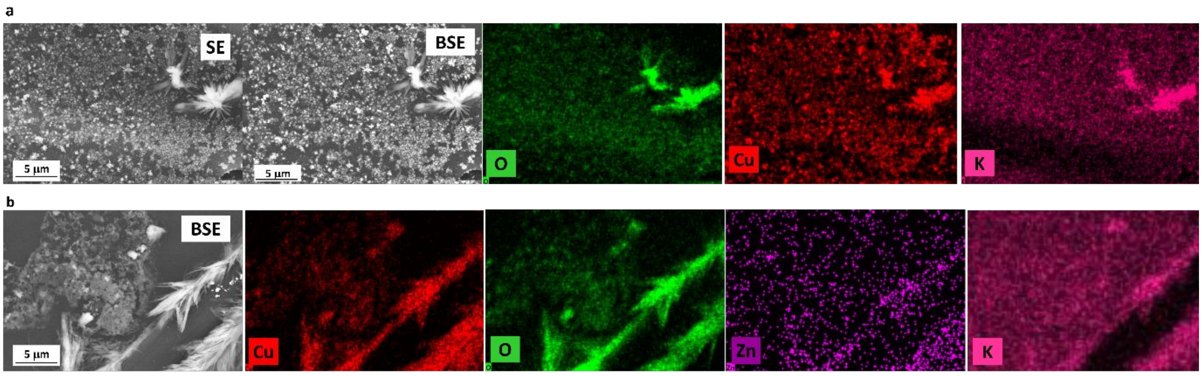

Figure S8 – SEM after the operando hard X-ray spectroscopy study for (a) Cu/G; b) CuZn/G.

2.4 Soft X-ray operando spectroscopy

2.4.1 Electrocatalytic performances during in situ Soft X-ray absorption spectroscopy

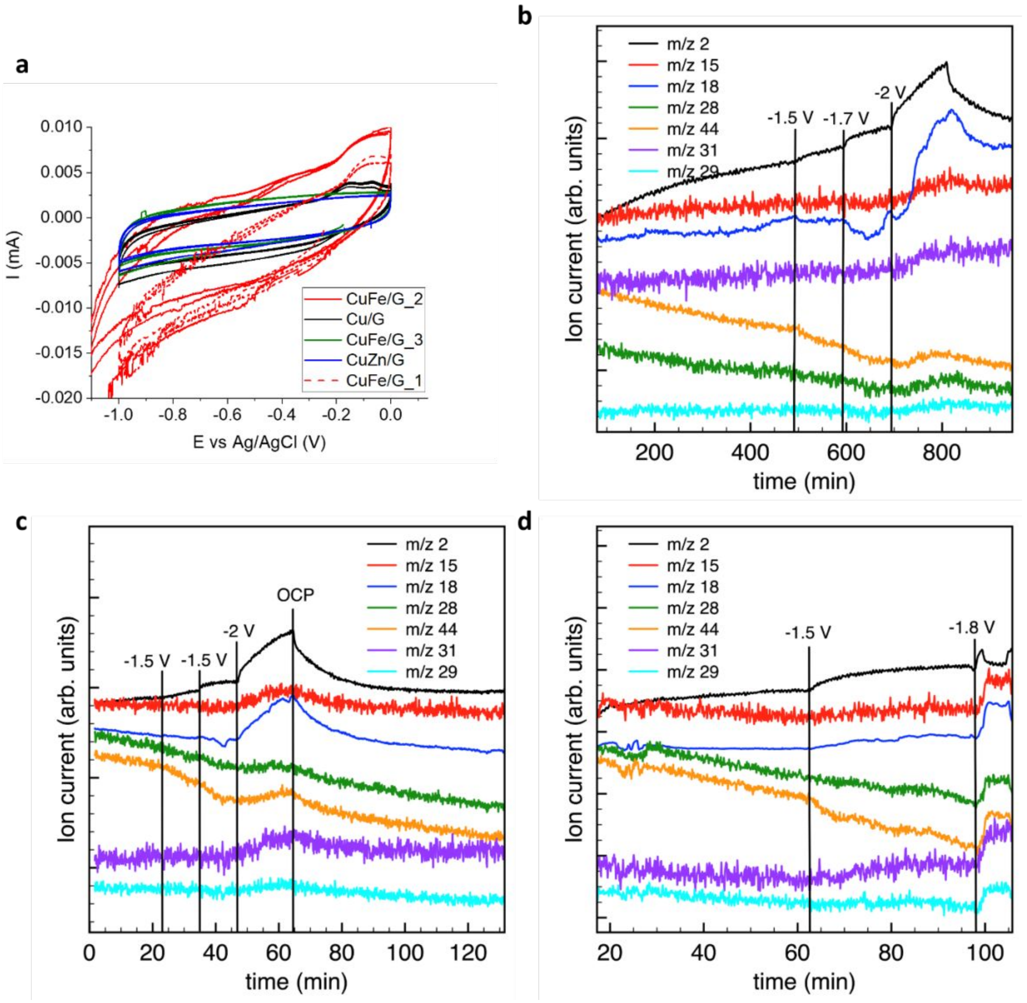

Figure S9: (a) Cyclic voltammograms recorded in the in situ cell, preceding the in situ spectroscopic measurements for the electrocatalysts as indicated; (b) Mass spectrum recorded during the chronoamperometry study for Cu/G in Figure S11a; (c) Mass spectrum recorded during the chronoamperometry study for sample CuZn/G in Figure 5a-c; (d) Mass spectrum recorded during the chronoamperometry study for sample CuFe/G in Figure 5d-f.

Table S6: Average currents exchanged during the chronoamperometry under steady state at each voltage applied.

<table>
<tr><td></td><td colspan="4">CuFe/G _1 MS in Figure S9d</td><td colspan="4">Cu/G MS in Figure S9b</td></tr>
<tr><td rowspan="2">Current (mA)</td><td colspan="2">-1.5 V</td><td colspan="2">-1.8 V</td><td>-0.5 V</td><td>-1.5 V</td><td>-1.7 V</td><td>-2V</td></tr>
<tr><td colspan="2">-0.56±0.04</td><td colspan="2">-2.08 ±0.28</td><td>-1E-03±0.0005</td><td>-1.74E-01 ±0.02</td><td>-4.68E-01 ±0.05</td><td>-1.28 ±0.06</td></tr>
<tr><td></td><td colspan="4">CuZn/G MS in Figure S9c</td><td colspan="4">CuFe/G _2 MS in Figure S10a</td></tr>
<tr><td></td><td>-0.5 V</td><td>-1.5 V</td><td>1.7 V</td><td>-2V</td><td>-0.5 V</td><td>-1.5 V</td><td>-1.7 V</td><td>-2V</td></tr>
<tr><td></td><td>-5.5E-04 ±0.00042</td><td>-1.5E-01 ±0.025</td><td>-4.5E-01 ±0.1</td><td>-1.35E-00 ±0.135</td><td>-1E-03±0.0005</td><td>-1E-01±0.03</td><td>-4E-01±0.01</td><td>-1.2-±0.03</td></tr>
</table>

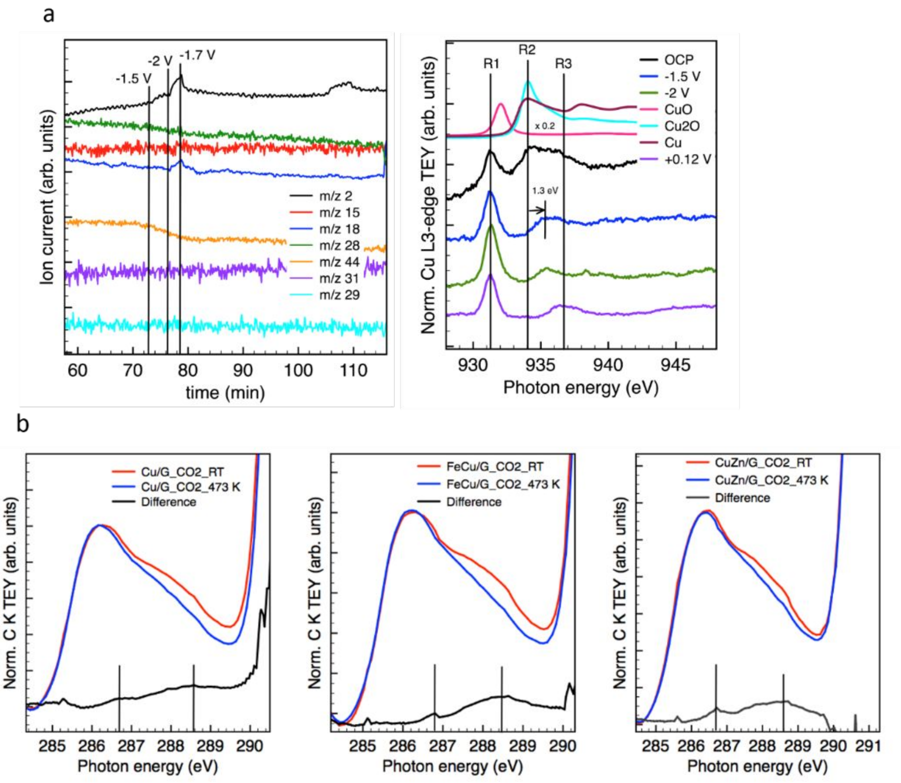

Figure S10: (a) Mass spectrum recorded during the chronoamperometry study for sample CuFe/G and relative Cu L_3-edge spectrum; (b) C K-edge NEXAFS measured for the as prepared samples as indicated under 0.1 mbar CO_2 at room temperature and 473 K. The lines in the difference spectrum indicate resonances related to desorbed species at 473 K.

2.4.2. L-edges operando spectroscopy study of Cu/G

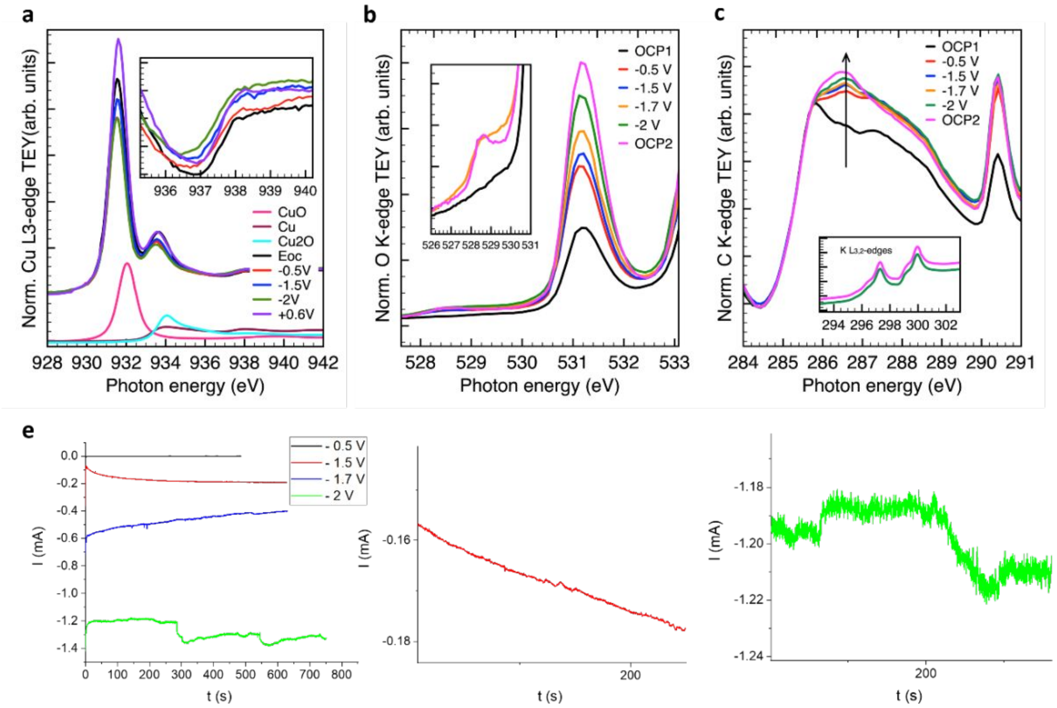

Figure S 11: Soft X-ray in situ NEXAFS data under a stagnant $KHCO_3$ electrolyte at different voltages as indicated: Cu L_3-edge a), O K-edge b) and C K-edge c) spectra for Cu/G. e) current measured for Cu/G at the different potentials vs Ag/AgCl as indicated and details of the current fluctuations.

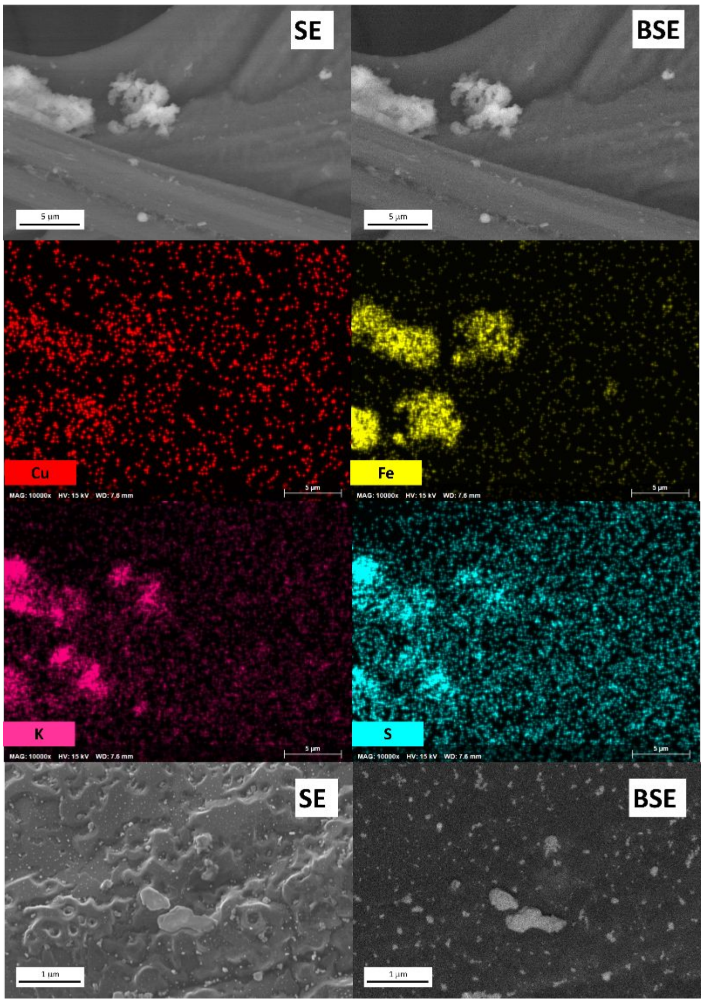

Figure S12 – SEM after the operando soft X-ray spectroscopy study for CuFe/G-1 under observed CO_2RR selective performances.

2.5 Simulation of the Cu L edge NEXAFS

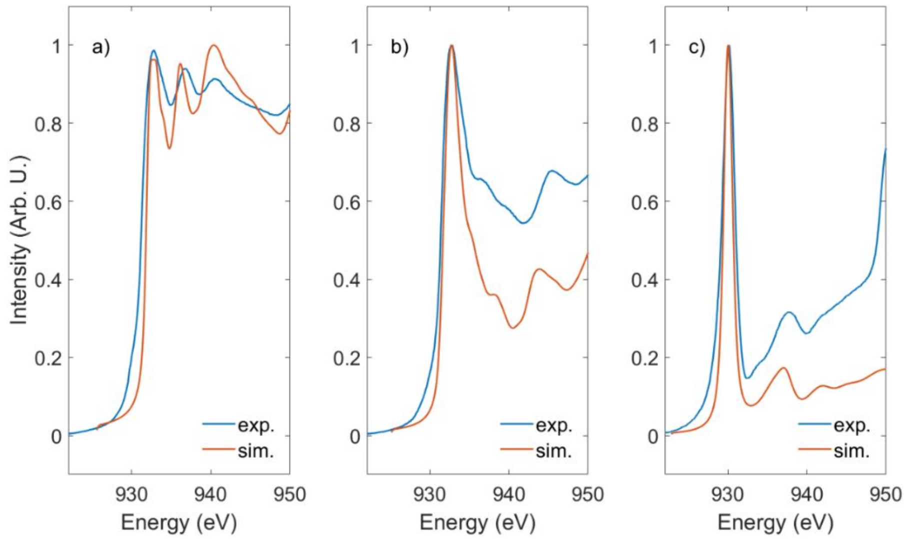

Figure S 13: Comparative plot of experimental and simulated Cu L_3 Absorption edges for reference materials a) Cu metal, b) Cu_2O and c) CuO. Simulated spectra have been energy-shifted to overlap with experimental ones.

Table S 7: Simulated Mulliken population following the introduction of either Zn or Fe (upper and lower panel, respectively) in supercell of Cu_2O and relaxation of the geometry.

Zn Substitution %	“s”	“p”	“d”	Total	Net Charge
0	0.62	0.39	9.65	10.65	0.35
12	0.69	0.49	9.65	10.83	0.17
25	0.72	0.51	9.64	10.87	0.12
37	0.72	0.54	9.64	10.9	0.09
50	0.75	0.59	9.65	10.99	0.002

Fe Substitution %	“s”	“p”	“d”	Total	Net Charge
0	0.62	0.39	9.65	10.65	0.35
12	0.61	0.46	9.64	10.71	0.29
25	0.60	0.47	9.65	10.73	0.26
37	0.60	0.52	9.66	10.79	0.21
50	0.64	0.55	9.68	10.88	0.12

Table S 8: Simulated Mulliken population following the introduction of either Zn or Fe (upper and lower panel, respectively) in supercell of $Cu(OH)_2$ and relaxation of the geometry of the unoccupied orbitals, and consequently the transition probability and spectral intensity.

Zn Substitution %	“s”	“p”	“d”	Total	Net Charge
0	0.47	0.23	9.44	10.14	0.86
12	0.50	0.26	9.45	10.21	0.78
25	0.52	0.30	9.45	10.27	0.72
37	0.53	0.32	9.45	10.30	0.70
50	0.54	0.32	9.45	10.32	0.68

Fe Substitution %	“s”	“p”	“d”	Total	Net Charge
0	0.47	0.23	9.44	10.14	0.86
12	0.47	0.23	9.48	10.18	0.81
25	0.47	0.23	9.52	10.23	0.77
37	0.46	0.24	9.56	10.26	0.73
50	0.46	0.23	9.61	10.31	0.69

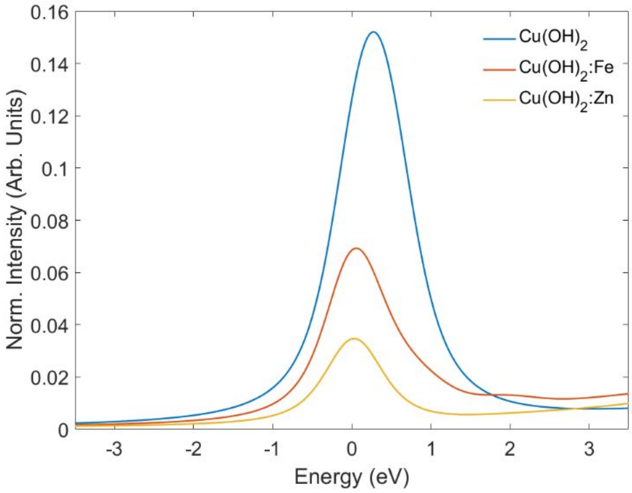

Figure S 14: Simulated Cu L_3 XANES intensity at the 2p-3d transitions, showing the decrease in intensity of the feature due to the lowest unoccupied d- and s-type electronic states.

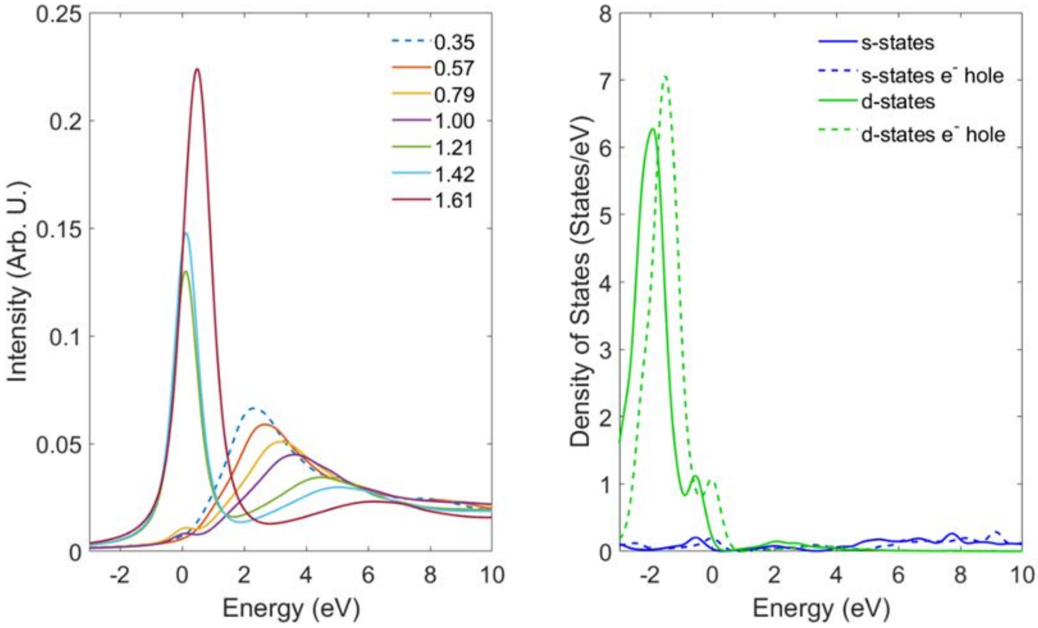

Figure S 15: (left panel) Simulated Cu L3 XANES intensity while increasing the Mulliken charge on Cu as a consequence of presence of electron-hole on neighbouring oxygen. (right panel) Corresponding Cu Density of states for the values of Mulliken charge of 0.35 eV (continuous line) and 0.79 eV (dashed line).

2.6 Computational Study

2.6.1 ZnO/Cu model

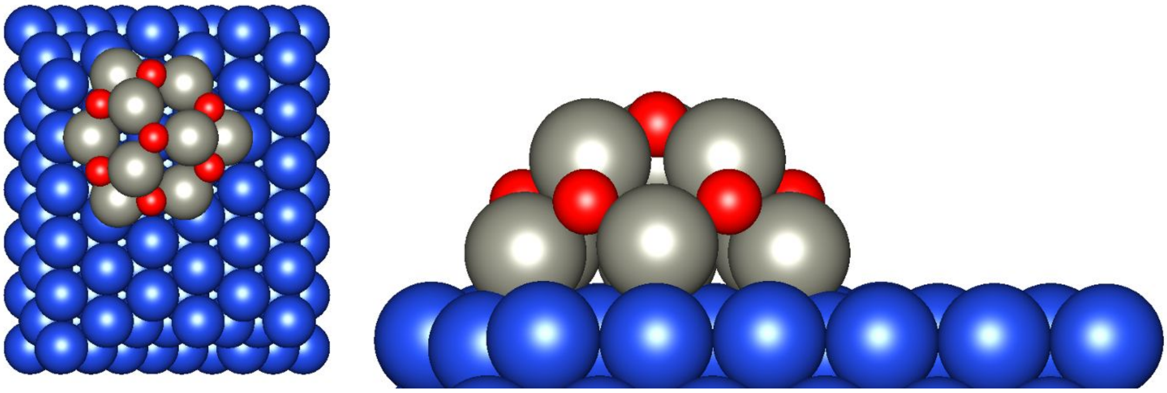

Figure S 16: Graphic depicting the model Cu/ZnO system, from the top (left) and side (right).

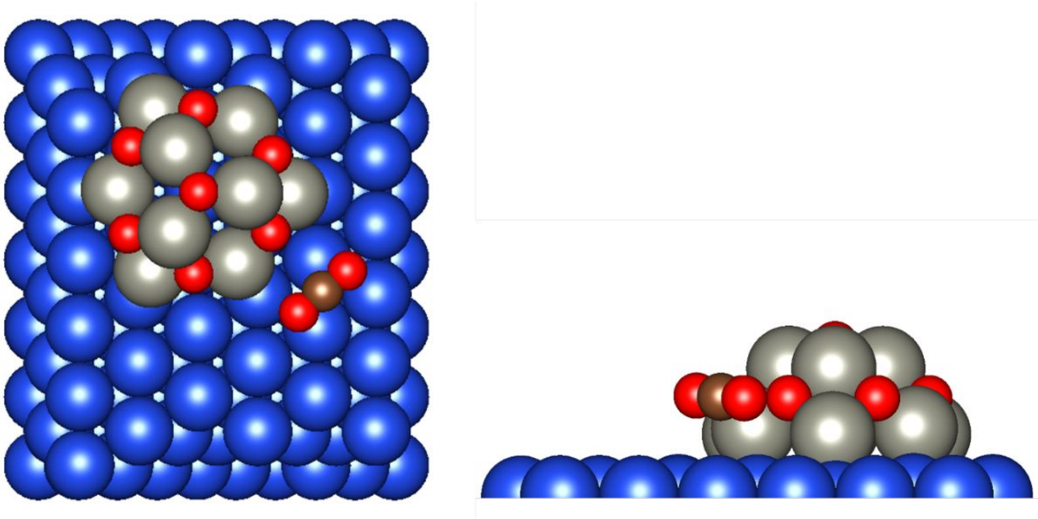

Figure S 17: Graphic depicting CO_2 physisorption on the Cu site of the model Cu/ZnO system, from the top (left) and side (right).

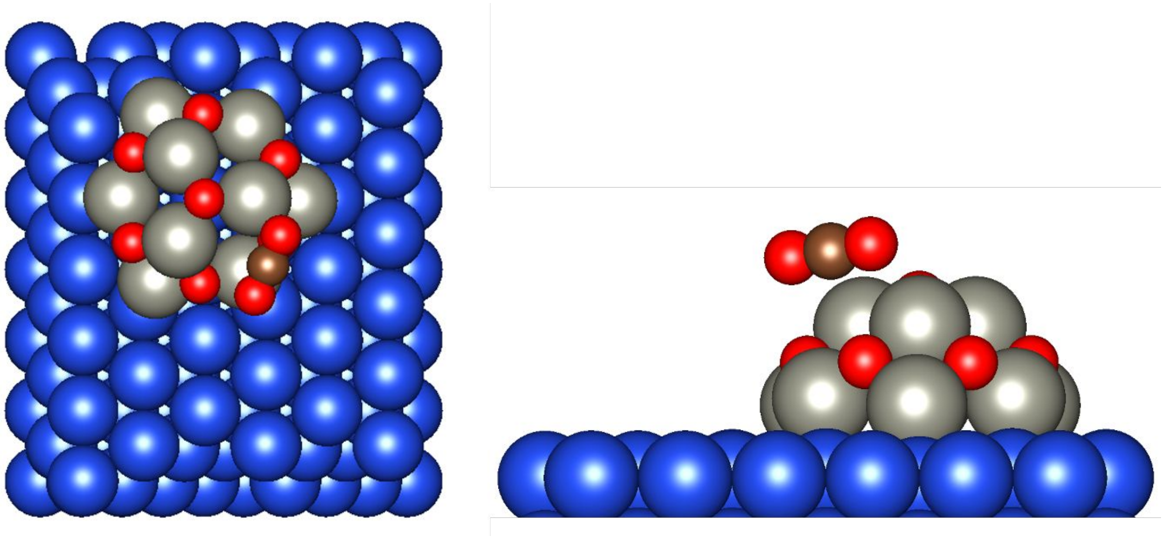

Figure S 18: Graphic depicting CO_2 physisorption on the ZnO site of the model Cu/ZnO system, from the top (left) and side (right).

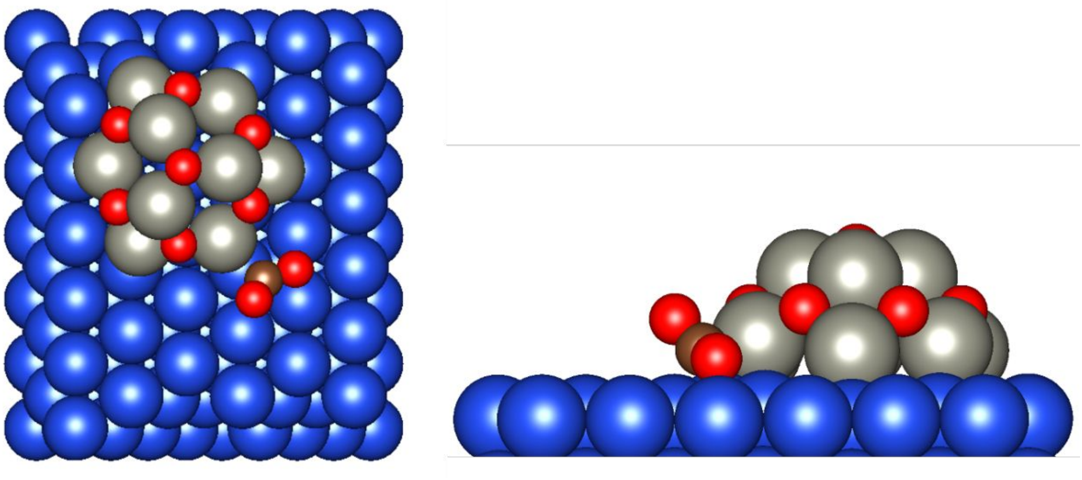

Figure S 19: Graphic depicting the metastable bent CO_2 on the Cu site of the model Cu/ZnO system, from the top (left) and side (right).

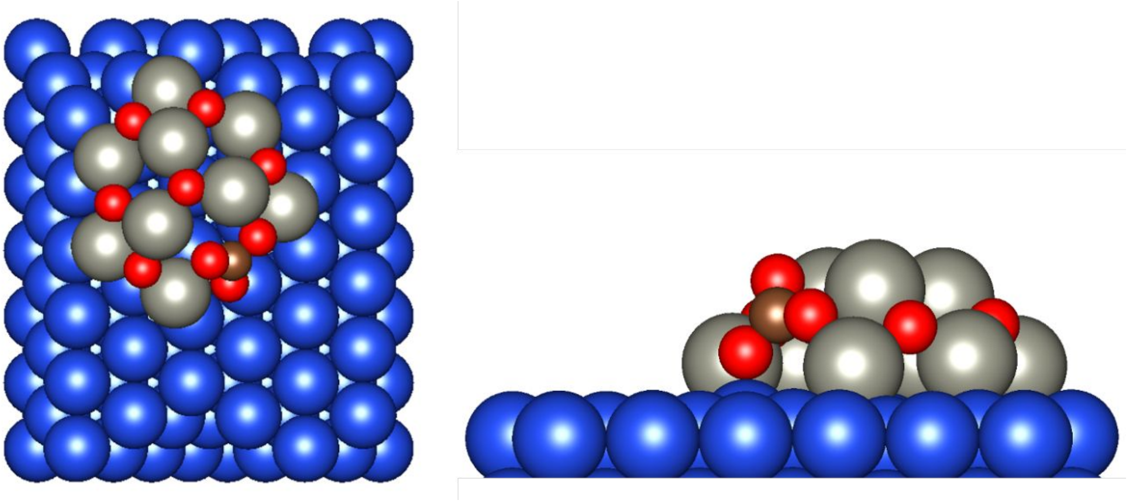

Figure S 20: Graphic depicting the carbonate-like species on the Cu/ZnO interfacial site of the model Cu/ZnO system, from the top (left) and side (right).

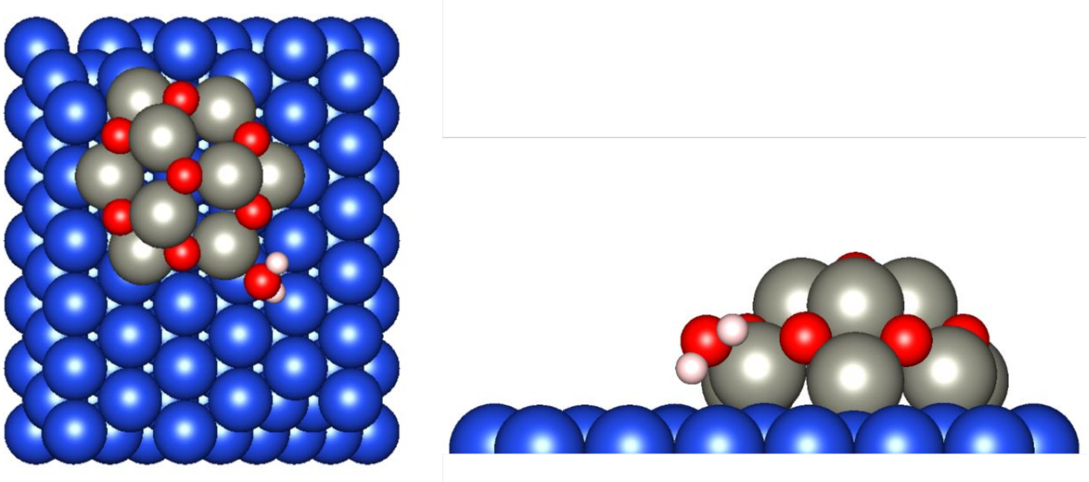

Figure S 21: Graphic depicting H_2O adsorption on the Cu site of the model Cu/ZnO system, from the top (left) and side (right).

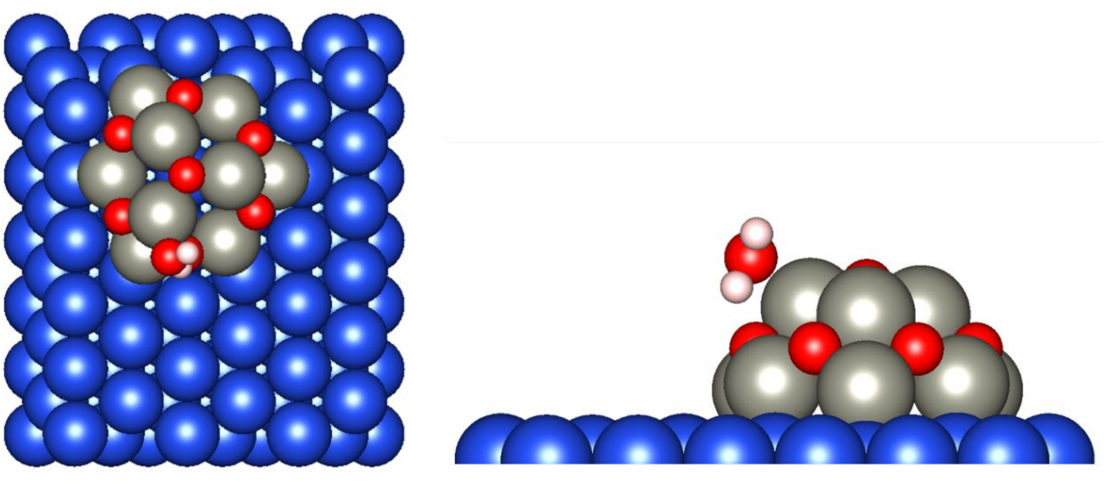

Figure S 22: Graphic depicting H_2O adsorption on the ZnO site of the model Cu/ZnO system, from the top (left) and side (right).

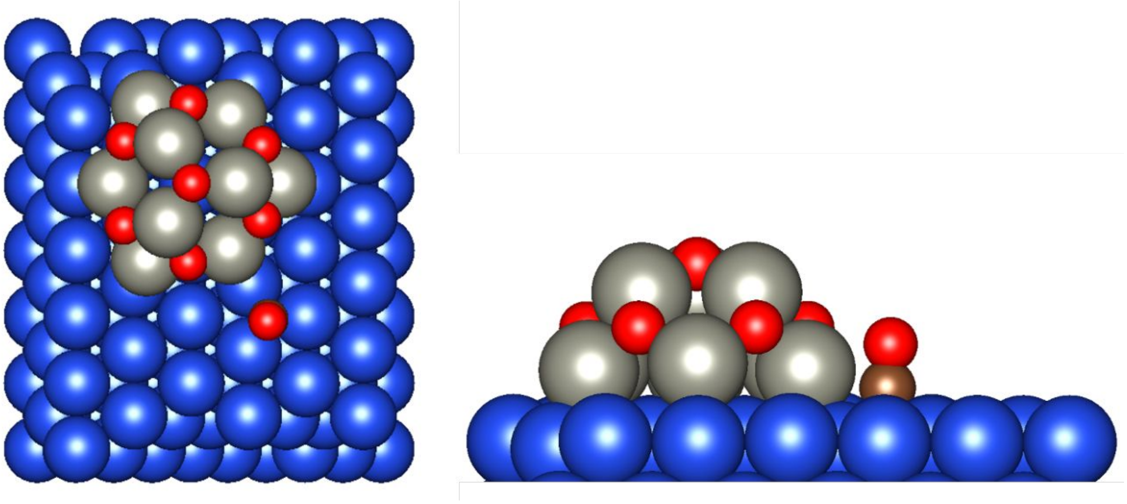

Figure S 23: Graphic depicting CO adsorption on the Cu site of the model Cu/ZnO system, from the top (left) and side (right).

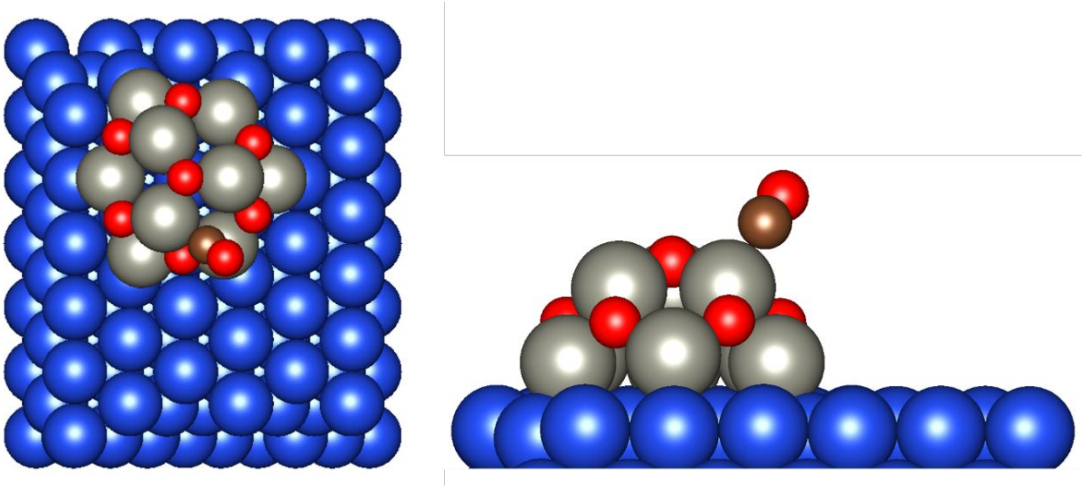

Figure S 24: Graphic depicting CO adsorption on the ZnO site of the model Cu/ZnO system, from the top (left) and side (right).

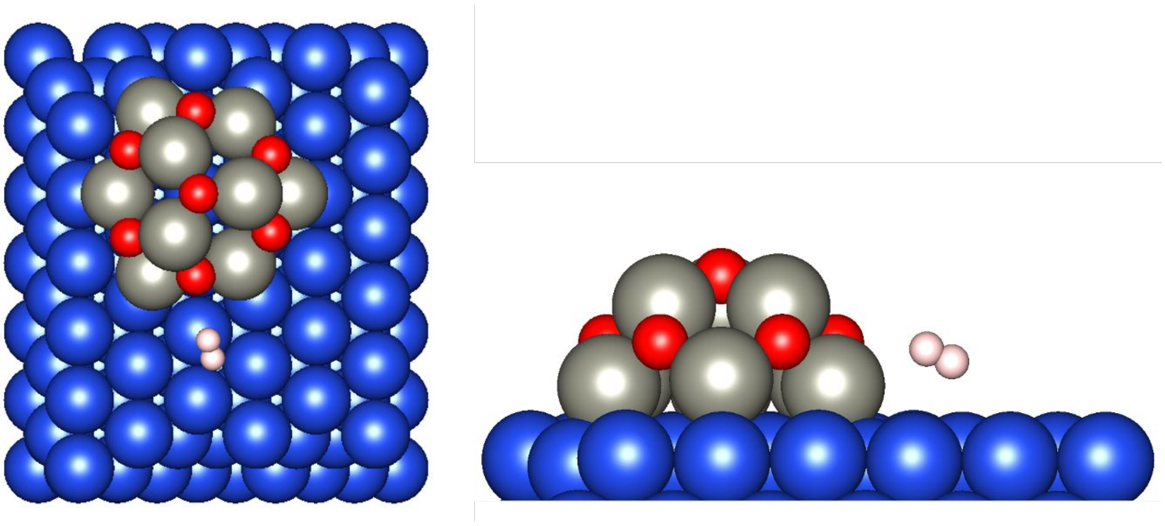

Figure S 25: Graphic depicting H_2 physisorption on the Cu site of the model Cu/ZnO system, from the top (left) and side (right).

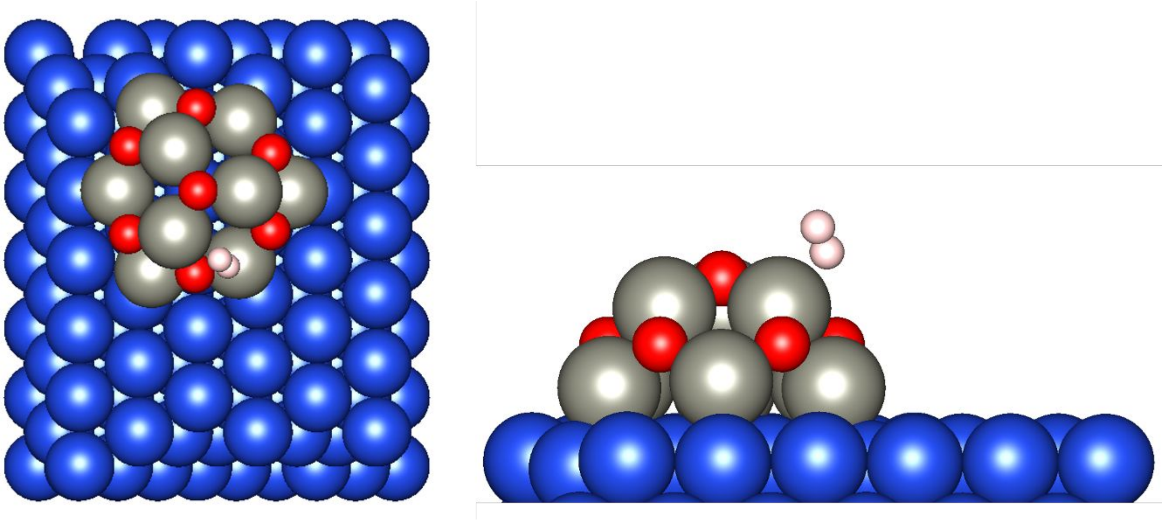

Figure S 26: Graphic depicting H_2 physisorption on the ZnO site of the model Cu/ZnO system, from the top (left) and side (right).

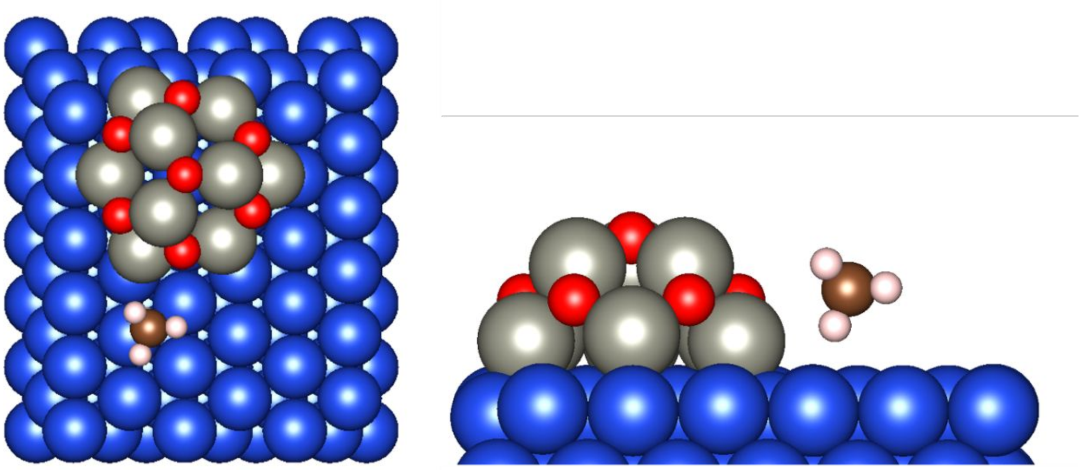

Figure S 27: Graphic depicting CH_4 physisorption on the Cu site of the model Cu/ZnO system, from the top (left) and side (right).

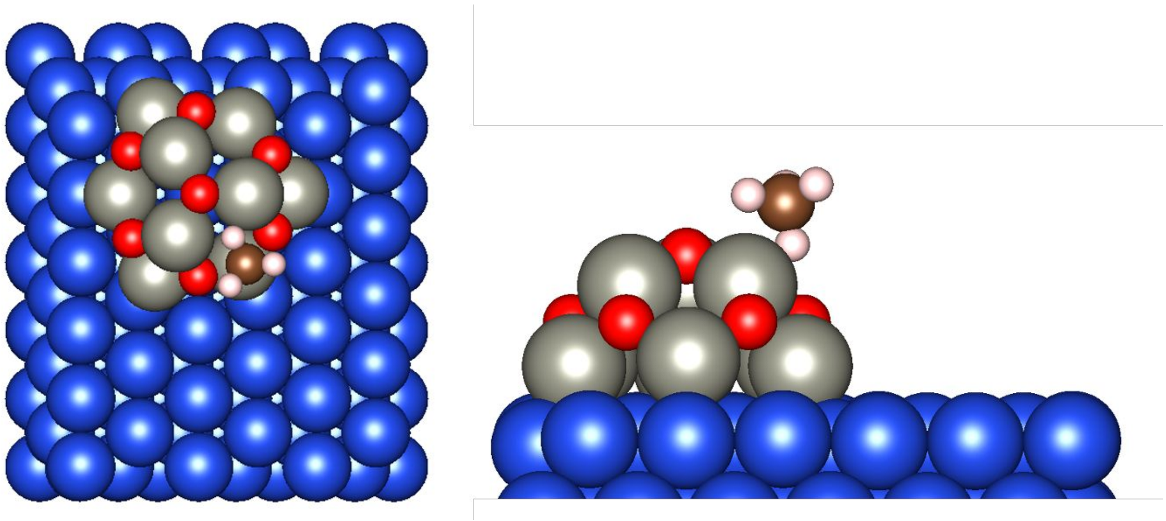

Figure S 28: Graphic depicting CH_4 physisorption on the ZnO site of the model Cu/ZnO system, from the top (left) and side (right).

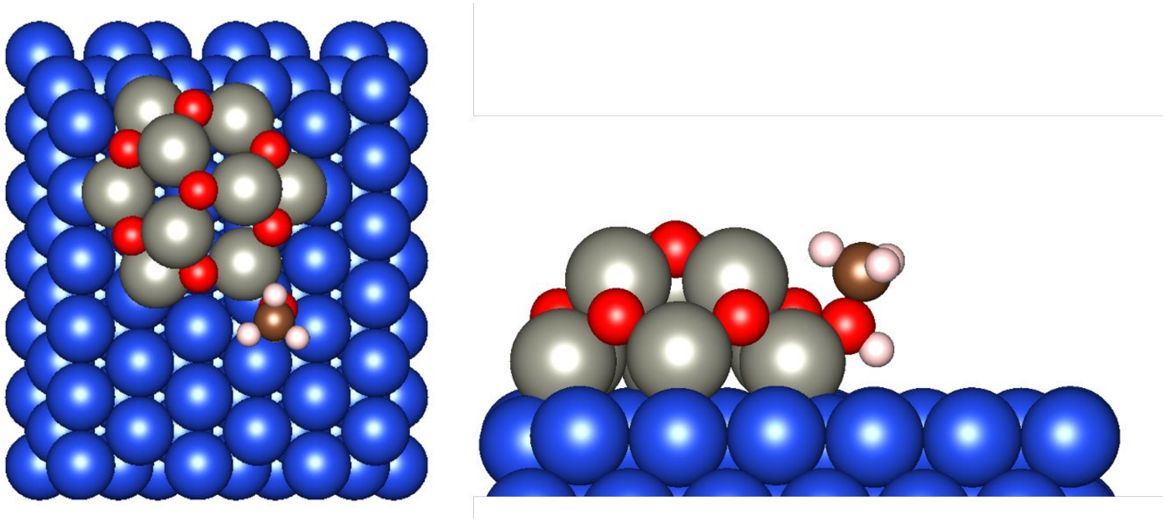

Figure S 29: Graphic depicting MeOH physisorption on the Cu site of the model Cu/ZnO system, from the top (left) and side (right).

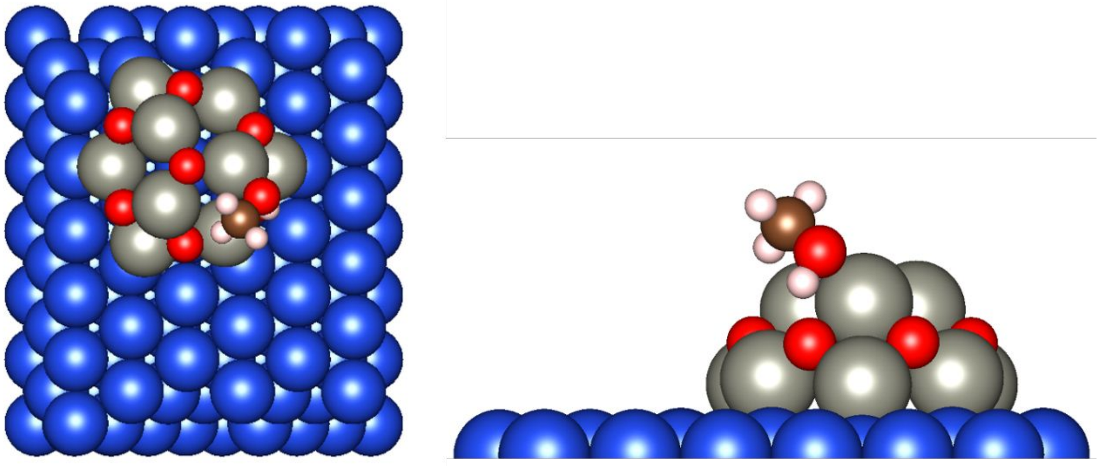

Figure S 30: Graphic depicting MeOH physisorption on the ZnO site of the model Cu/ZnO system, from the top (left) and side (right).

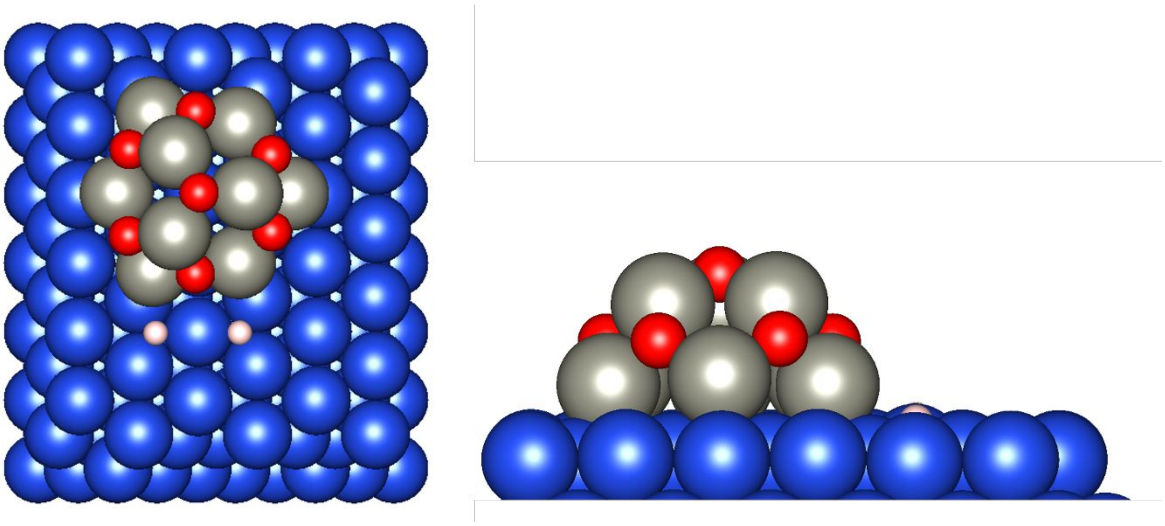

Figure S 31: Graphic depicting H_2 dissociation on the Cu site of the model Cu/ZnO system, from the top (left) and side (right).

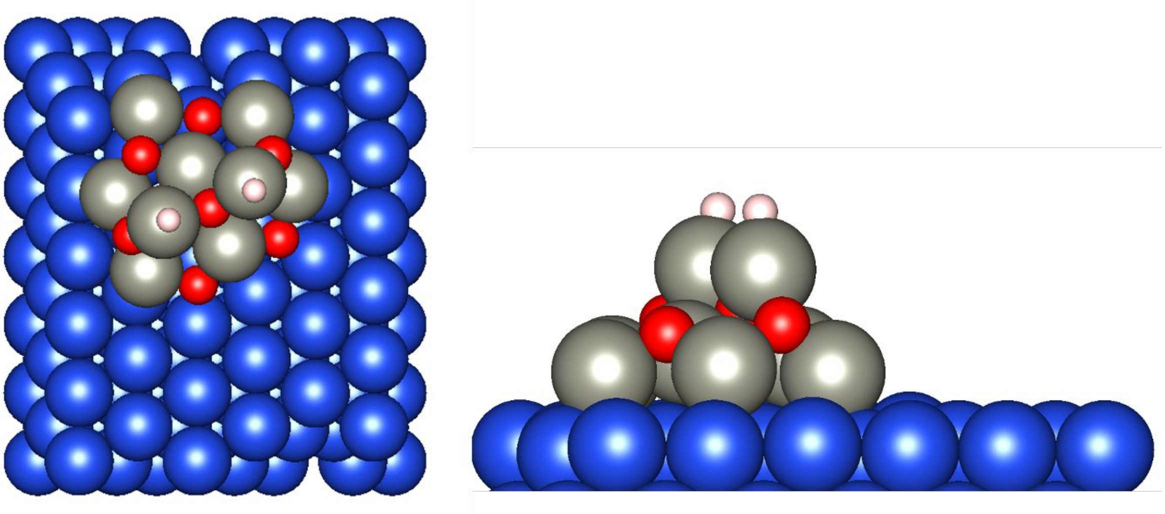

Figure S 32: Graphic depicting H_2 dissociation on the ZnO site of the model Cu/ZnO system, from the top (left) and side (right).

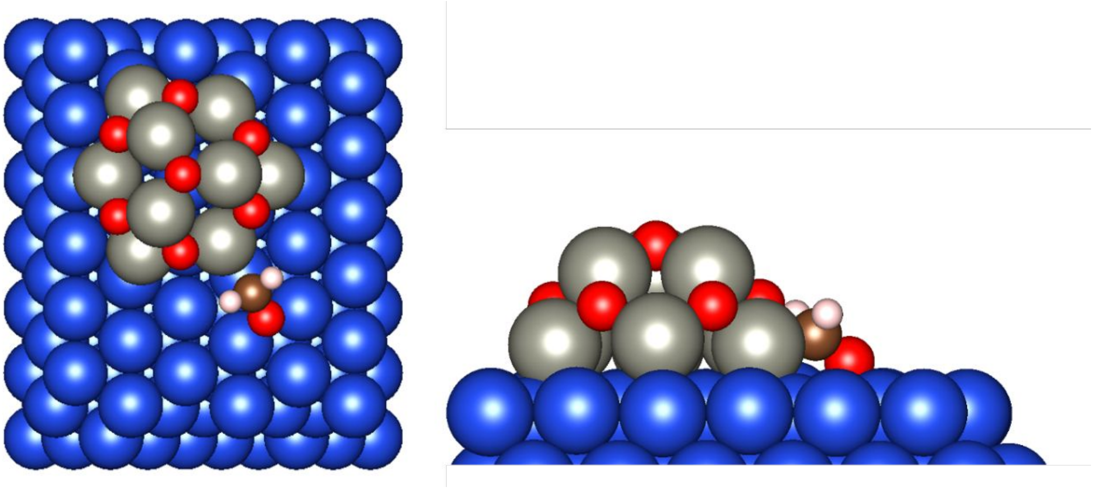

Figure S 33: Graphic depicting formaldehyde adsorption on the Cu site of the model Cu/ZnO system, from the top (left) and side (right).

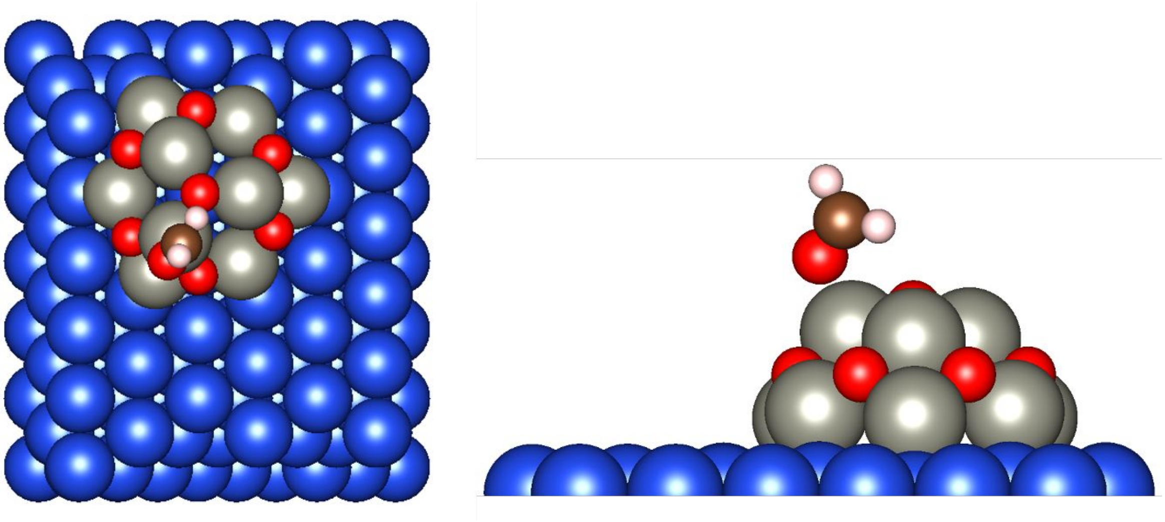

Figure S 34: Graphic depicting formaldehyde adsorption on the ZnO site of the model Cu/ZnO system, from the top (left) and side (right).

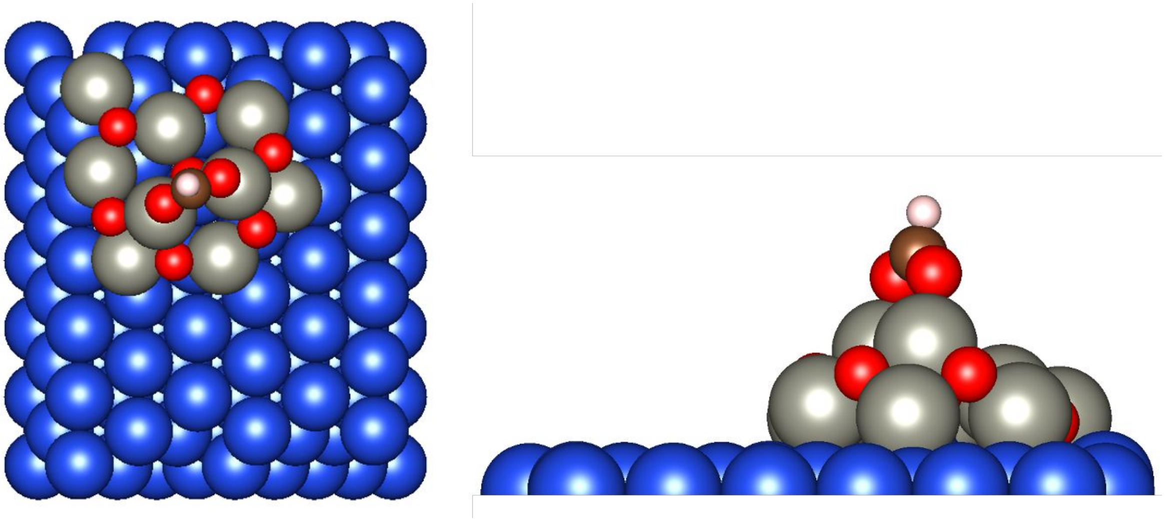

Figure S 35: Graphic depicting the bidentate formate species on the Zn-Zn site of the model Cu/ZnO system, from the top (left) and side (right).

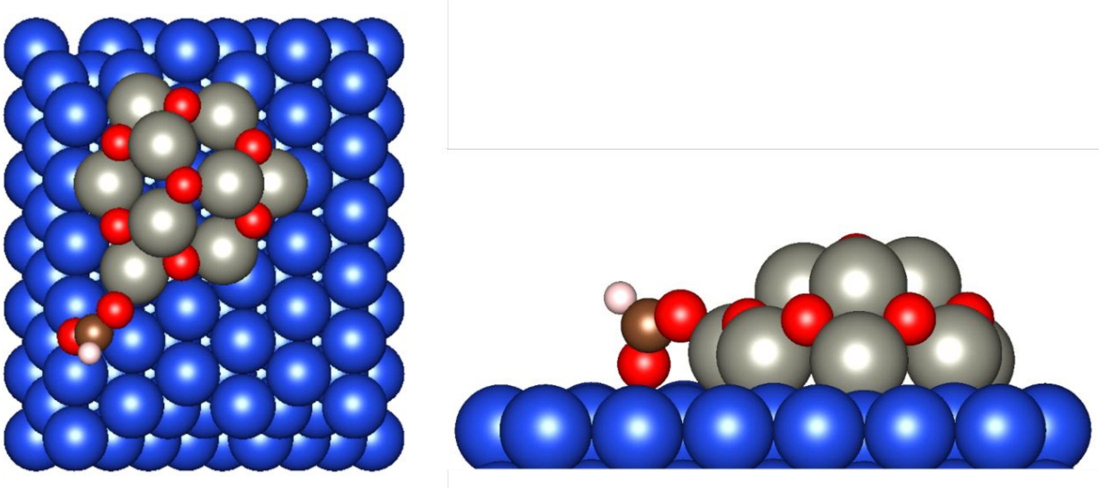

Figure S 36: Graphic depicting the bidentate formate species on the Cu-Zn site of the model Cu/ZnO system, from the top (left) and side (right).

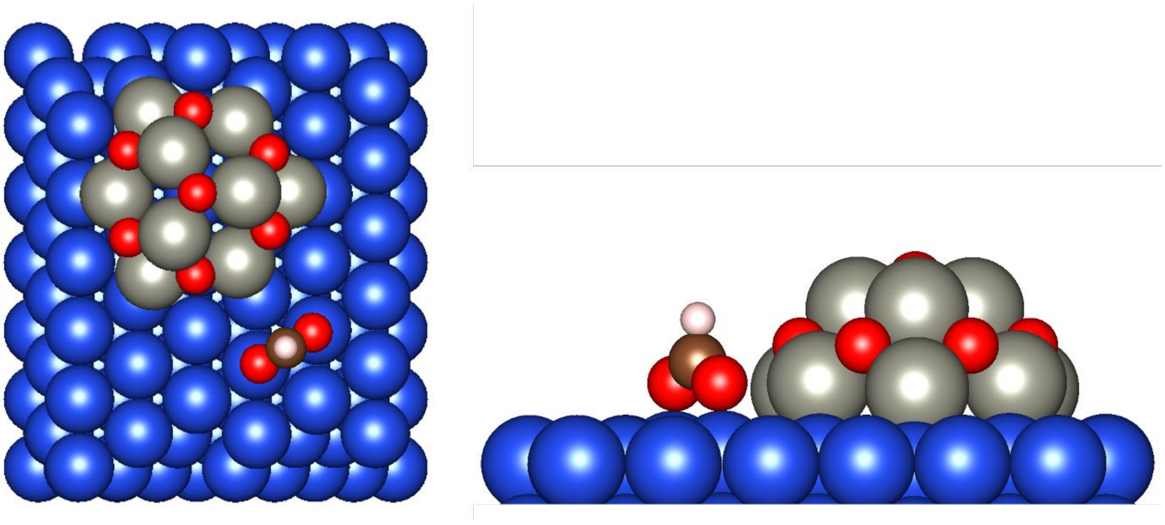

Figure S 37: Graphic depicting the bidentate formate species on the Cu-Cu site of the model Cu/ZnO system, from the top (left) and side (right).

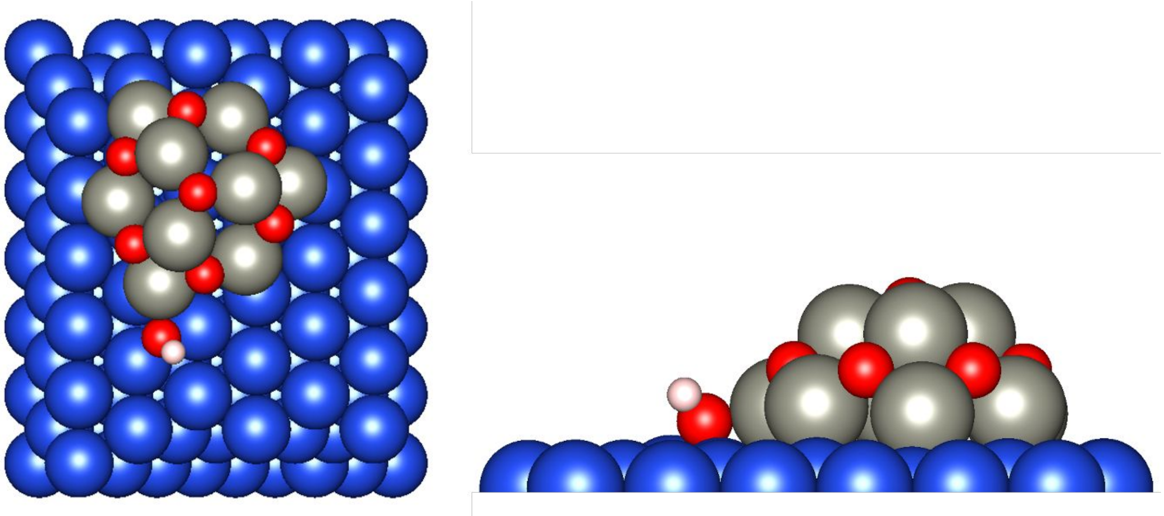

Figure S 38: Graphic depicting OH adsorption on the Cu site of the model Cu/ZnO system, from the top (left) and side (right).

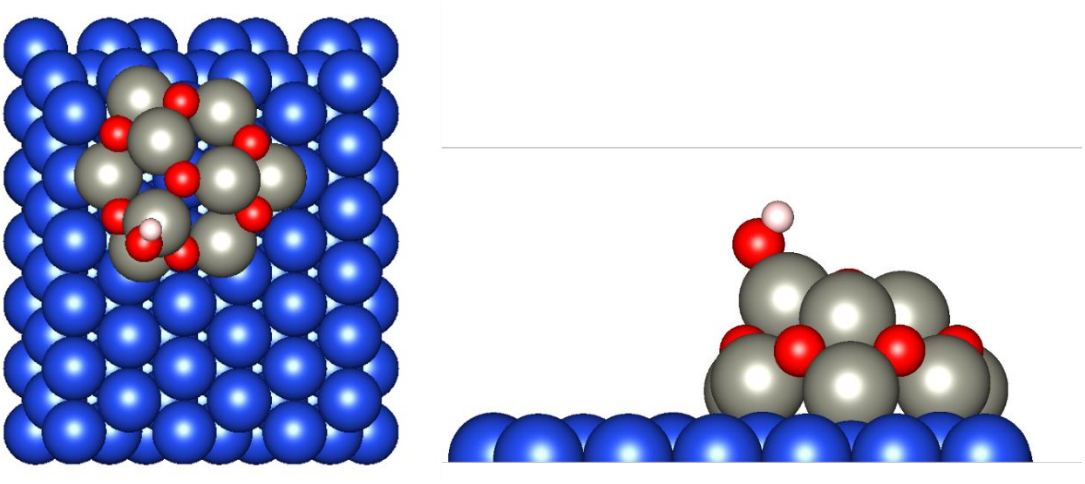

Figure S 39: Graphic depicting OH adsorption on the ZnO site of the model Cu/ZnO system, from the top (left) and side (right).

2.6.2 ZnCu alloy model

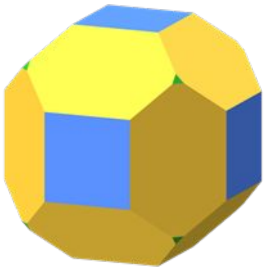

Figure S 40: Wulff construction of a theoretical nanoparticle with 75.8 percent fraction of the {011} facet (yellow), a 24 percent fraction of the {001} facet (blue) and 0.4 percent fraction of the {111} surface (green).

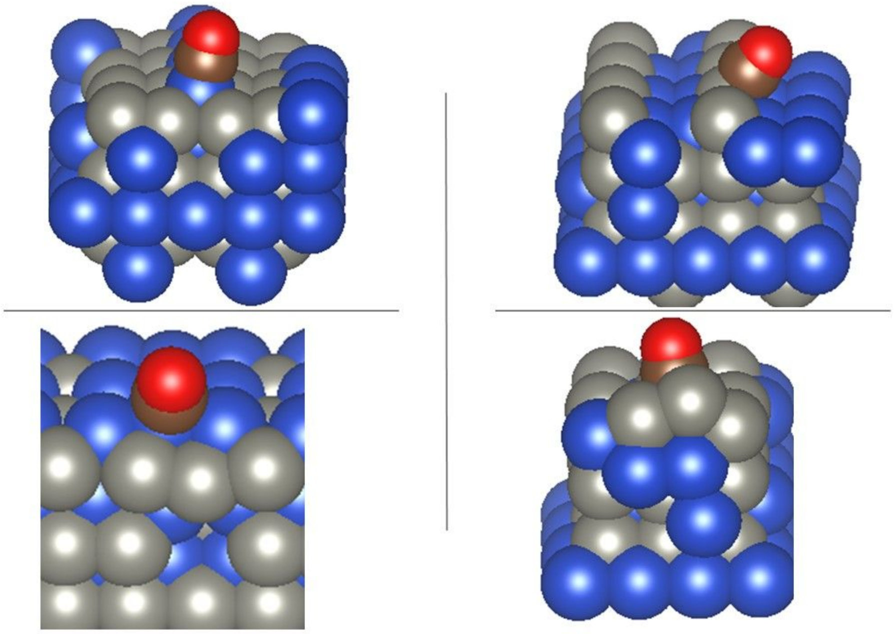

Figure S 41: CO adsorption on four (001) surfaces of CuZn: (top left) reconstructed Cu_{top}, (top right) reconstructed Zn_{top}, (bottom left) unreconstructed Cu_{top}, (bottom right) unreconstructed Zn_{top}.

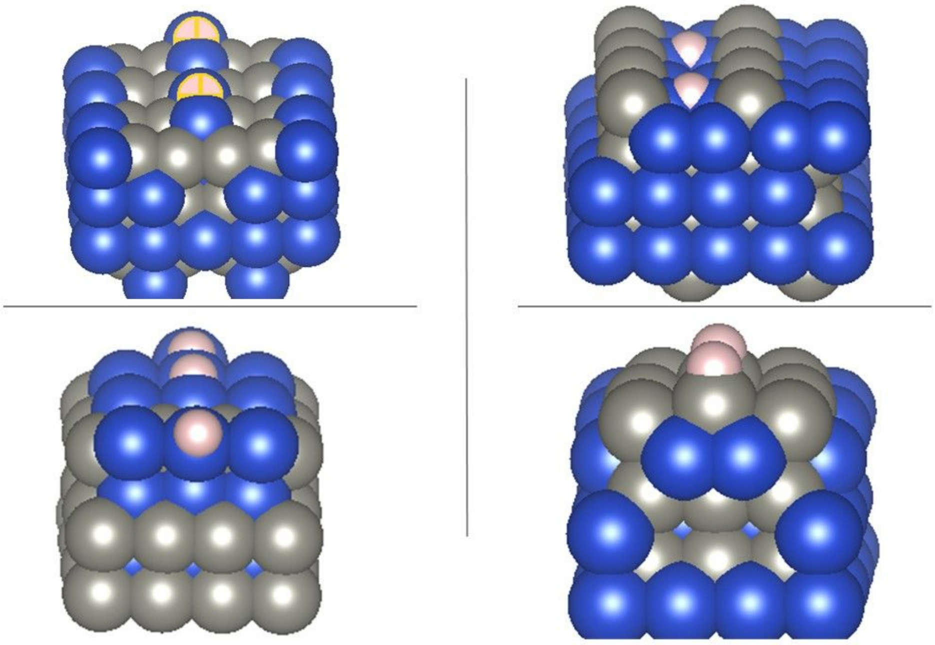

Figure S 42: H_2 adsorption on four (001) surfaces of CuZn: (top left) reconstructed Cu_{top}, (top right) reconstructed Zn_{top}, (bottom left) unreconstructed Cu_{top}, (bottom right) reconstructed Zn_{top}.

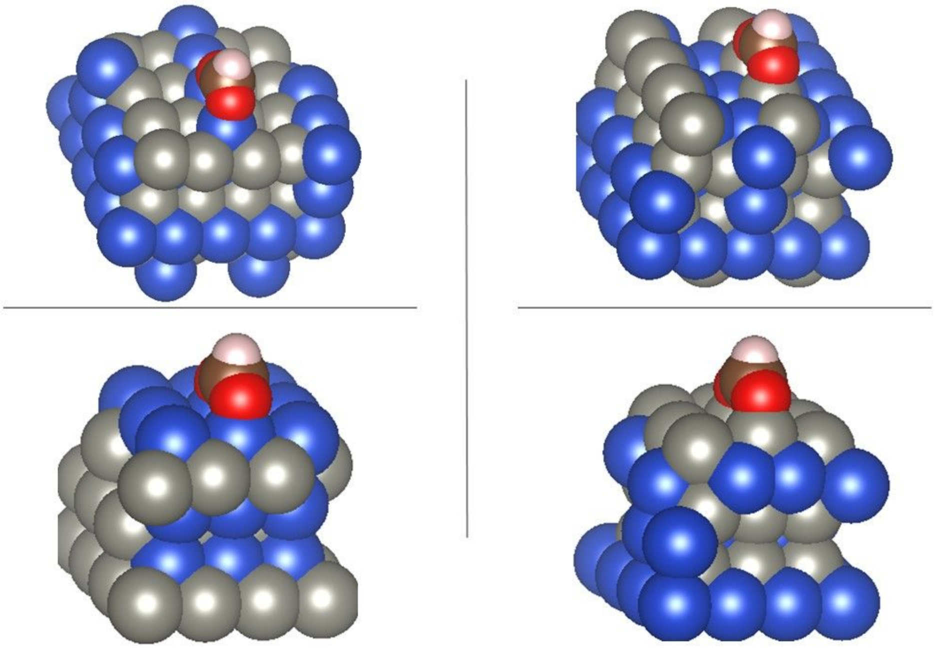

Figure S 43: Formate adsorption on four (001) surfaces of CuZn: (top left) reconstructed Cu_{top}, (top right) reconstructed Zn_{top}, (bottom left) unreconstructed Cu_{top}, (bottom right) reconstructed Zn_{top}.

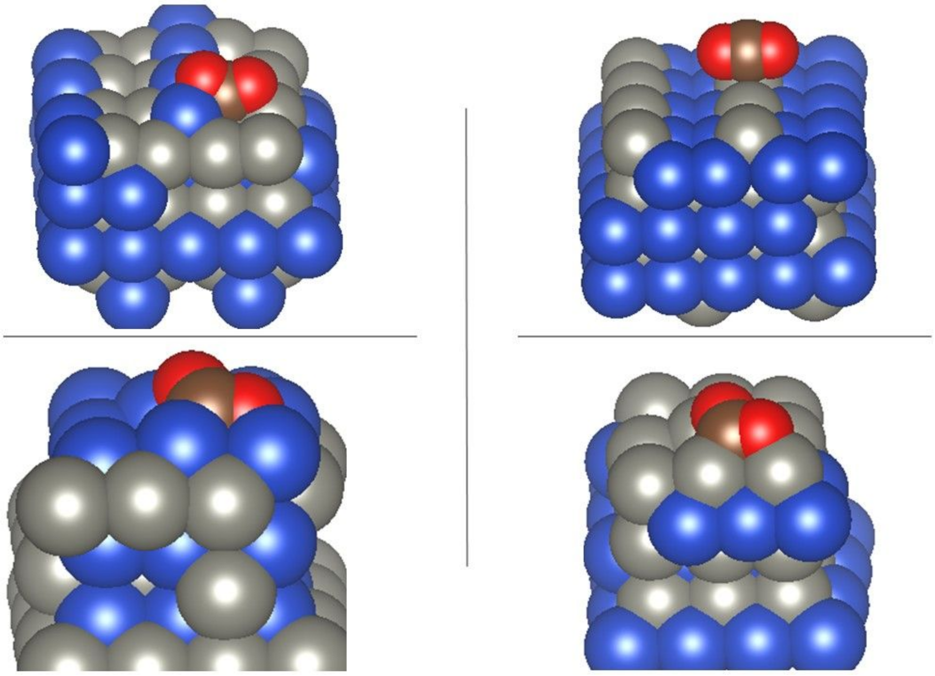

Figure S 44: CO_2 adsorption on four (001) surfaces of CuZn: (top left) reconstructed Cu_{top}, (top right) reconstructed Zn_{top}, (bottom left) unreconstructed Cu_{top}, (bottom right) reconstructed Zn_{top}.

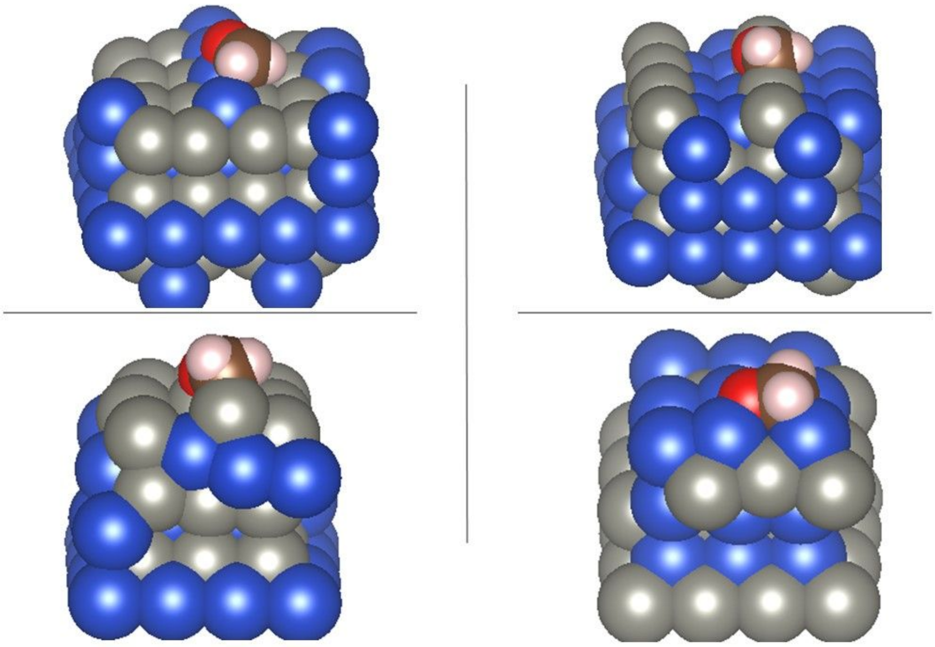

Figure S 45: Formaldehyde adsorption on four (001) surfaces of CuZn: (top left) reconstructed Cu_{top}, (top right) reconstructed Zn_{top}, (bottom left) unreconstructed Cu_{top}, (bottom right) reconstructed Zn_{top}.

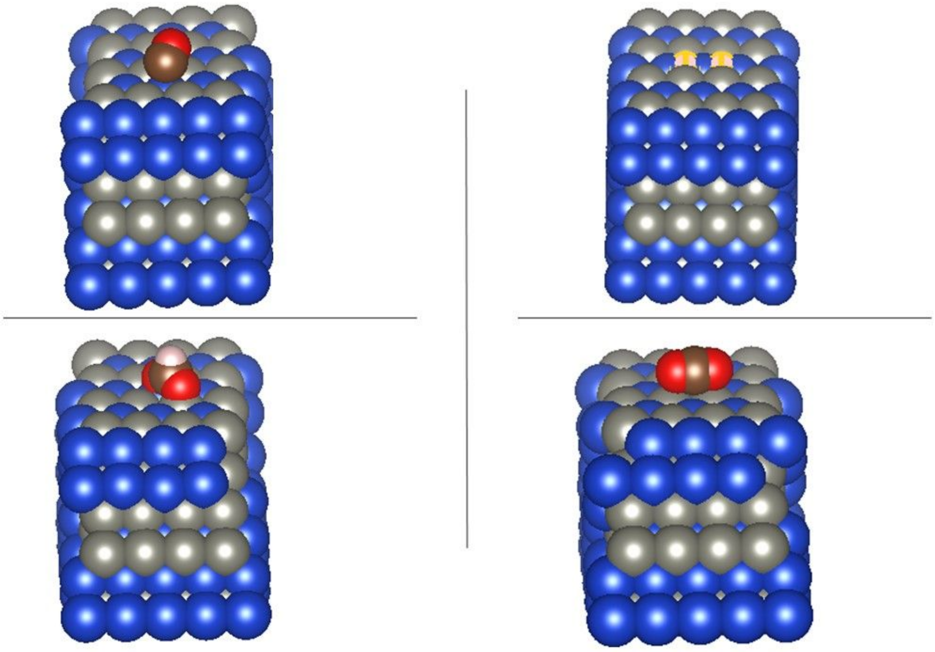

Figure S 46: Adsorption modes of: CO (top left), H_2 (top right), formate (bottom left) and CO_2 (bottom left) on the 110 facet of CuZn.

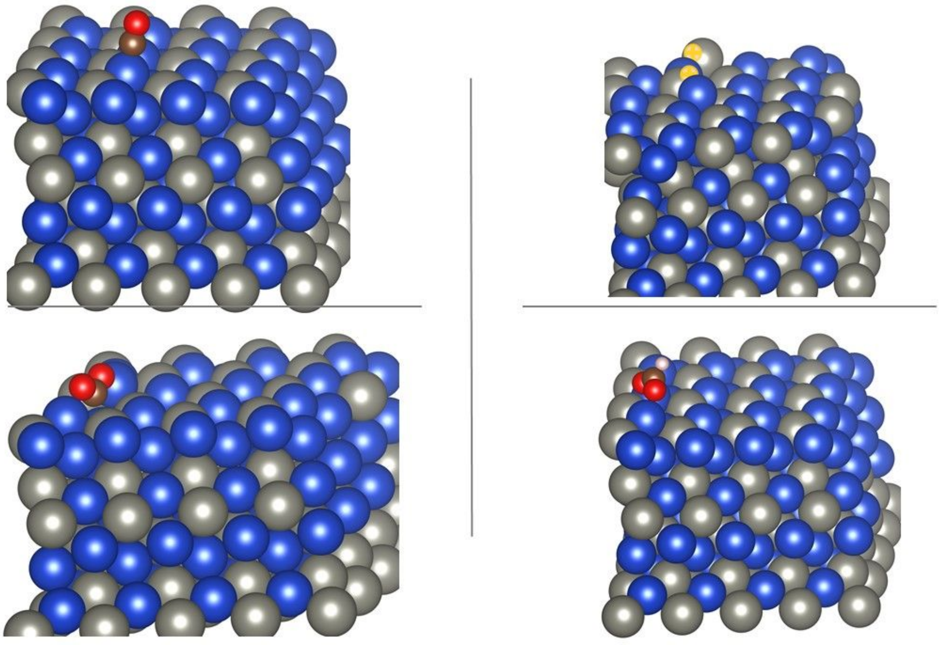

Figure S 47: Adsorption modes of: CO (top left), H_2 (top right), CO_2 (bottom left) and Formate (bottom left) on the 110 facet of CuZn.

Table S9: Adsorption energies for important species onto the low index surfaces of CuZn

	[001] Cu_{top}^{a}	[001] Cu_{top}^{b}	[001] Zn_{top}^{a}	[001] Zn_{top}^{b}	[110]	[111] Cu_{top}	[111] Zn_{op}
**CO_2**	-0.16	-0.34	-0.67	-0.86	0.19	0.20	-0.06
**H_2**	-0.26	-0.56	-0.75	0.01	-0.45	-0.10	-0.07
**H_2CO**	-0.65	-1.13	-0.70	-1.53	-5.28	-0.62	-0.78
**CO**	-1.10	-1.21	-0.48	-1.04	-1.00	-1.14	-1.31
**HCOO**	-1.42	-1.42	-1.51	-2.02	-1.08	-1.16	-0.74
**CH_4**	-0.20	-0.32	-0.19	-0.19	-0.18	-0.22	-0.18
**H_2O**	-0.37	-0.74	-0.17	-0.81	-0.49	-0.58	-0.49
OH H	-0.52	-0.49	0.08	-0.18	-0.57	-0.47	-0.26

[a][001] surfaces reconstructed to remove any perpendicular dipole, [b][001] unreconstructed surface.

REFERENCES

(1) C. Genovese, M. E. Schuster, E. K. Gibson, D. Gianolio, V. Posligua, R. Grau-Crespo, G. Cibin, P. P. Wells, D. Garai, V. Solokha, S. Krick Calderon, J. Velasco Velez, C. Ampelli, S. Perathoner, G. Held, G. Centi, and R. Arrigo, Operando spectroscopy study of the carbon dioxide electro-reduction by iron species on nitrogen-doped carbon. *Nature Commun*. **2018**, 9, 935.

(2) B. Ravel, and M. Newville, ATHENA, ARTEMIS, HEPHAESTUS: data analysis for X-ray absorption spectroscopy using IFEFFIT, *J. Synchrotron Radiation* **2005**, 12, 537–541.

(3) R. Arrigo, R. Blume, V. Streibel, C. Genovese, A. Roldan, M. E. Schuster, C. Ampelli, S. Perathoner, J.-J. Velasco-Vélez, M. Hävecker, A. Knop-Gericke, R. Schlögl, and G. Centi, Dynamics at Polarized, Carbon Dioxide/Iron Oxyhydroxide Interfaces Unveil the Origin of Multicarbon Product Formation, *ACS Catal*. **2021**, 12, 411-430.

(4) M. C. Biesinger, Advanced analysis of copper X-ray photoelectron spectra*, Surf. Interface Anal.* **2017**, 49, 1325–1334.

(5) R. Arrigo, R. Blume, A. Large, J.-J. Velesco-Velez, M. Haevecker, A. Knop-Gericke, and G. Held, Dynamics over a Cu-graphite electrode during the gas-phase CO2 reduction investigated by APXPS, Faraday Discuss., 2022, Accepted Manuscript. DOI: 10.1039/D1FD00121C

(6) M. T. Greiner, T. E. Jones, B. E. Johnson, T. C. R. Rocha, Z. J. Wang, M. Armbrüster, M. Willinger, A. Knop-Gericke and R. Schlögl, The oxidation of copper catalysts during ethylene epoxidation, *Phys. Chem. Chem. Phys*., **2015**,17, 25073-25089

(7) P. Thakur, V. Bisogni, J. C. Cezar, N. B. Brookes, G. Ghiringhelli, S. Gautam, K. H. Chae, M. Subramanian, R. Jayavel and K. Asokan, Electronic structure of Cu-doped ZnO thin films by x-ray absorption, magnetic circular dichroism, and resonant inelastic x-ray scattering, *J. Appl. Phys.* **2010**, 107, 103915.

(8) F. Zhao, M. Kasrai, T.-K. Sham, and Z. Bai, Characterization of Tribofilms Generated from Serpentine and Commercial Oil Using X-ray Absorption Spectroscopy. *Tribology Lett.* **2013**, 50, 287-297.

(9) T. K. Sham, A. Hiraya, and M. Watanabe, Electronic structure of Cu-Au alloys from the Cu perspective: A Cu $L_{3,2}$-edge study, *Phys. Rev. B*, **1997**, 55, 7585.
